# The Ephrin tyrosine kinase a3 (EphA3) is a novel mediator of RAGE-prompted motility of breast cancer cells

**DOI:** 10.1186/s13046-023-02747-5

**Published:** 2023-07-12

**Authors:** Marianna Talia, Francesca Cirillo, Asia Spinelli, Azzurra Zicarelli, Domenica Scordamaglia, Lucia Muglia, Salvatore De Rosis, Damiano Cosimo Rigiracciolo, Gianfranco Filippelli, Ida Daniela Perrotta, Mariano Davoli, Rosanna De Rosa, Rachele Macirella, Elvira Brunelli, Anna Maria Miglietta, Bruno Nardo, Daniela Tosoni, Salvatore Pece, Ernestina Marianna De Francesco, Antonino Belfiore, Marcello Maggiolini, Rosamaria Lappano

**Affiliations:** 1 Department of Pharmacy, Health and Nutritional Sciences, University of Calabria, 87036 Rende, Italy; 2 European Institute of Oncology IRCCS, Via Ripamonti 435, 20141 Milan, Italy; 3 Oncology Department, Hospital of Paola, 87100 Cosenza, Italy; 4 Department of Biology, Ecology and Earth Science, University of Calabria, 87036 Rende, Italy; 5 Breast and General Surgery Unit, Regional Hospital Cosenza, 87100 Cosenza, Italy; 6 Department of Oncology and Hemato-Oncology, Università Degli Studi Di Milano, 20142 Milan, Italy; 7 Endocrinology Unit, Department of Clinical and Experimental Medicine, University of Catania, Garibaldi-Nesima Hospital, Catania, 95122 Italy

**Keywords:** Breast cancer, Receptor for advanced glycation end-products (RAGE), EphA3, Cancer-associated fibroblast (CAFs)

## Abstract

**Background:**

The receptor for advanced glycation-end products (RAGE) and its ligands have been implicated in obesity and associated inflammatory processes as well as in metabolic alterations like diabetes. In addition, RAGE-mediated signaling has been reported to contribute to the metastatic progression of breast cancer (BC), although mechanistic insights are still required. Here, we provide novel findings regarding the transcriptomic landscape and the molecular events through which RAGE may prompt aggressive features in estrogen receptor (ER)-positive BC.

**Methods:**

MCF7 and T47D BC cells stably overexpressing human RAGE were used as a model system to evaluate important changes like cell protrusions, migration, invasion and colony formation both in vitro through scanning electron microscopy, clonogenic, migration and invasion assays and in vivo through zebrafish xenografts experiments. The whole transcriptome of RAGE-overexpressing BC cells was screened by high-throughput RNA sequencing. Thereafter, Gene Ontology (GO) and Kyoto Encyclopedia of Genes and Genomes (KEGG) pathway enrichment analyses allowed the prediction of potential functions of differentially expressed genes (DEGs). Flow cytometry, real time-PCR, chromatin immunoprecipitation, immunofluorescence and western blot assays were performed to investigate the molecular network involved in the regulation of a novel RAGE target gene namely EphA3. The clinical significance of EphA3 was explored in the TCGA cohort of patients through the survivALL package, whereas the pro-migratory role of EphA3 signaling was ascertained in both BC cells and cancer-associated fibroblasts (CAFs). Statistical analysis was performed by t-tests.

**Results:**

RNA-seq findings and GSEA analysis revealed that RAGE overexpression leads to a motility-related gene signature in ER-positive BC cells. Accordingly, we found that RAGE-overexpressing BC cells exhibit long filopodia-like membrane protrusions as well as an enhanced dissemination potential, as determined by the diverse experimental assays. Mechanistically, we established for the first time that EphA3 signaling may act as a physical mediator of BC cells and CAFs motility through both homotypic and heterotypic interactions.

**Conclusions:**

Our data demonstrate that RAGE up-regulation leads to migratory ability in ER-positive BC cells. Noteworthy, our findings suggest that EphA3 may be considered as a novel RAGE target gene facilitating BC invasion and scattering from the primary tumor mass. Overall, the current results may provide useful insights for more comprehensive therapeutic approaches in BC, particularly in obese and diabetic patients that are characterized by high RAGE levels.

**Supplementary Information:**

The online version contains supplementary material available at 10.1186/s13046-023-02747-5.

## Background

Breast cancer (BC) is the most common malignancy worldwide among women. The incidence of BC has risen over the past decades and is expected to further increase by more than 46% by 2040 [1, 2]. The efforts seeking to identify the risk factors of BC and to decrease their exposure are warranted to curb the future burden of this malignancy. Certain metabolic abnormalities correlated with obesity and type 2 diabetes (T2DM), such as hyperglycemia and hyperinsulinemia, may contribute to the progression of many types of tumors, including BC [3–5]. In this regard, it has been reported that obesity and T2DM are associated with a high risk of developing BC along with increased mortality rates [6–10].

Diabetic and obese patients display augmented circulating levels of advanced glycation end-products (AGEs), which have been shown both to accumulate in cancer tissues and to associate with diverse diseases beyond cancer like diabetes, arthritis, cardiovascular and neurodegenerative disorders [11–16]. The receptor for AGEs (RAGE) is a member of the immunoglobulin superfamily [17] and a multiligand recognition receptor implicated in physiological homeostasis, development, inflammation and several illnesses [18–21]. Other than AGEs, RAGE can bind to different ligands including high-mobility group protein (B)1 (HMGB1), S-100 calcium-binding protein, amyloid-β-protein, Mac-1 and phosphatidylserine [22]. RAGE and many of its ligands are overexpressed in diabetes, chronic inflammation and various malignancies [23, 24]. As it concerns BC, the high expression of RAGE and certain ligands, such as HMGB1, S100A8, S100A9, S100A11, S100P, S100A7, correlates with poor clinical outcomes [25–28]. RAGE-mediated signaling activates several transduction pathways and transcription factors [28, 29], which in turn stimulate oxidative stress and inflammation leading to the growth and metastasis of BC and other types of tumors [17, 30, 31]. For instance, the RAGE-induced activation of MAPK, PI3K/Akt, JAK/STAT and NF-κB transduction pathways may trigger the production of inflammatory cytokines and growth factors that promote cancer cell survival, proliferation, migration, invasion and angiogenesis [29, 32]. In accordance with these findings, it has been shown that RAGE silencing impairs tumor growth and angiogenesis, prevents the recruitment of inflammatory cells and reduces lung and liver metastases in estrogen receptor (ER)-negative BC cells [30]. In addition, it has been demonstrated that RAGE-mediated signaling mediates the crosstalk between malignant cells and the surrounding tumor microenvironment (TME), thus sustaining cancer growth, angiogenesis, autophagy, epigenetic changes, stemness properties and metastatic dissemination [28, 30, 32, 33].

The spread of cancer cells into the surrounding tissues and the vasculature is the first step of the metastatic process, which represents the most life-threatening aspect of the disease [34]. In BC, cell invasion is a multi-factorial and transcriptional-coordinated process that triggers cytoplasmic protrusions, cytoskeletal rearrangements and recruitment of matrix-degrading enzymes [34]. In addition to the classical cell adhesion molecules, like cadherins and integrins, the tyrosine kinase Eph (Erythropoietin-producing hepatocellular) receptors and their ligands, namely ephrins, have been involved in cancer cell invasiveness [35–38]. Both Eph receptors and ephrins are membrane tethered and activated by cell–cell contact, leading to downstream signaling pathways that regulate actin cytoskeleton changes and then cell motility, adhesion and polarity [39]. Various Eph/ephrin family members have been found to be increased in tumor tissues and to promote cancer growth and invasion [40–42]. Moreover, a role for the Eph-dependent signaling has been recently assessed in repulsive homotypic contact inhibition of locomotion (CIL) among cancer cells and in unimpeded migration of metastatic tumor cells towards fibroblasts [43].

In the present study, we have characterized the RAGE-driven gene signature providing novel molecular mechanisms involved in its pro-tumorigenic action in ER-positive BC, which accounts for approximately 70% of all breast tumors [44]. In this vein, we have first established stable RAGE-overexpressing clones of human ER-positive BC cells and then we have assessed the role of RAGE-related gene signature in BC cell migration and invasion in vitro as well as in zebrafish embryos in vivo. Worthy, we have discovered that EphA3, as a main RAGE target gene, facilitates the motile phenotype of BC cells. Next, we have determined that the activation of the EphA3-mediated signaling increases the invasive potential of both BC cells and cancer-associated fibroblasts (CAFs) derived from BC patients, thus contributing to the cooperation between tumor and stromal cells in BC progression.

## Methods

### Cell lines and establishment of RAGE-overexpressing stable clones

MCF7 and T47D BC cells were obtained from ATCC (Manassas, VA, USA), used less than 6 months after resuscitation, routinely tested and authenticated according to the ATCC suggestions. MCF7 cells were maintained in DMEM/F-12 with phenol red, supplemented with 10% fetal bovine serum (FBS) and 100 μg/ml penicillin/ streptomycin. T47D cells were maintained in RPMI 1640 with phenol red supplemented with 10% FBS, 0.2 Units/ml bovine insulin and 100 μg/ml penicillin/streptomycin. For stable transfection, MCF7 and T47D cells were seeded at 2 × 10^6^ cells/well in a six-well plate and transfected with a pcDNA3-RAGE plasmid (a gift from Henri Huttunen, Addgene plasmid # 71,435; http://n2t.net/addgene:71435; RRID:Addgene 71435) [45] or empty vector pcDNA3 using TurboFect™ Transfection Reagent (Thermo Fisher Scientific, Life Technologies Italia, Monza, Italy) according to the manufacturer’s instructions. Stable clones were selected by continuously screening the above transfected cells with 500 μg/ml neomycin G418 (Merck Life Science, Milano, Italy). Afterwards, clones were maintained in the presence of 50 ng/ml neomycin to avoid loss of plasmids. RAGE overexpression in MCF7 and T47D cells was ascertained by immunoblotting experiments. All cell lines were grown in a 37 °C incubator with 5% CO_2_.

CAFs. CAFs were isolated, cultured and characterized as previously described [46] from 10 invasive mammary ductal carcinomas and pooled for the subsequent studies. Briefly, specimens were cut into 1–2 mm diameter pieces, placed in a digestion solution (400 IU collagenase, 100 IU hyaluronidase, 10% FBS, antibiotics and antimycotics) (Thermo Fisher Scientific) and incubated overnight at 37 °C. After centrifugation at 90 × g for 2 min, the super-natant containing fibroblasts was centrifuged at 485 × g for 8 min; the pellet obtained was suspended in Medium 199 and Ham’s F12 mixed 1:1 (supplemented with 10% FBS and 100 μg/ml penicillin/streptomycin). CAFs were then expanded into 10-cm Petri dishes and stored as cells passaged for three population doublings within a total 7 to 10 days after tissue dissociation. Primary cell cultures of fibroblasts were characterized by immunofluorescence with human anti-vimentin (V9) and human anti-cytokeratin 14 (LL001) (Santa Cruz Biotechnology, DBA, Milan, Italy). FAPα antibody (H-56; Santa Cruz Biotechnology) was used to assess fibroblast activation (data not shown). We used CAFs passaged for up to 10 population doublings for the experiments to minimize clonal selection and culture stress, which could occur during extended tissue culture. CAFs were grown in a 37◦C incubator with 5% CO2 and switched to a medium without serum and phenol red the day before treatments to be processed for experiments.

### Reagents

The EphA3 inhibitor AWL-II-38.3 was obtained from MedChem Express (DBA), mithramycin A (MMA) from Abcam (Euroclone S.p.A., Milan, Italy), CellTracker™ CM-DiI Dye from Thermo Fisher Scientific. All compounds were dissolved in dimethyl sulfoxide (DMSO).

### RNA-Seq Pipeline

Total RNA from MCF7/wt and MCF7/RAGE cells was extracted using RNeasy mini kit according to manufacturer's instructions (Qiagen, Bioset s.r.l., Catanzaro, Italy). RNA integrity for library preparation was determined by analysis of extracted total RNA using a 2100 Bioanalyzer (Agilent Technologies) with RNA 6000 NanoChip. RNA concentrations were measured using Qubit RNA Assay Kit. Libraries were prepared from total RNA according to manufacturer instructions with Illumina Stranded mRNA Prep kit. Libraries quality was evaluated by size analysis on 2100 Bioanlyzer (Chip DNA HS) and concentrations were determined using Qubit DNA HS assay kit (Thermo Fisher Scientific). Sequencing was performed on Illumina Novaseq 6000 in the 100PE format. Reads preprocessing was performed by using fastp v0.20.0 [47], applying specific parameters in order to remove residual adapter sequences and to keep only high-quality data (qualified_quality_phred = 20, unqualified_percent_limit = 30, average_qual = 25, low_complexity_filter = True, complexity_threshold = 30). The percentage of uniquely mapped reads resulted high with the mean value of 84% (mean value for sample: 60 million total reads, unmapped reads 7%, quality base > q30 94%). Then, passing filter reads were mapped to the human genome reference (version GRCh38) using STAR v2.7.0 [48] with standard parameters, except for sjdbOverhang option set on read length. Genome and transcripts annotation provided as input were downloaded from v99 of Ensembl repository. Alignments were then elaborated by RSEM v1.3.3 [49], to estimate transcript abundances. Subsequently, the sample-specific gene-level abundances were merged into a single raw expression matrix applying a dedicated RSEM command (rsem-generate-data-matrix). Genes with at least 10 counts in 50% of samples were then selected. Differential expression (pairwise comparisons) was computed by edgeR [50] from raw counts in each comparison. Re-annotation of previously differentially expressed genes (DEGs) was performed using the bioMart package [51] into R 3.6, querying available Ensembl Gene IDs and retrieving Gene Names and Entrez gene IDs.

### Data source and acquisition

In silico studies were performed analyzing the publicly available dataset TCGA (The Cancer Genome Atlas). The mRNA expression data (RNA Seq V2 RSEM) and the patient information of the TCGA Invasive Breast Cancer Cohort were retrieved from UCSC Xena (https://xenabrowser.net/) on the 22th December 2022. Samples (n. 1247) were filtered for missing values and by “sample type” in order to separate tumor tissues (n. 1104) from the adjacent normal tissues (n. 113). Furthermore, BC patients were classified on the basis of the presence or absence of the estrogen receptor (ER) detected by immunohistochemistry or according to the intrinsic molecular subtypes.

### Survival analysis

The survival analyses were performed using the TCGA gene expression data along with the disease specific survival (DSS),overall survival (OS) or progression free interval (PFI) information. The survivALL package was employed in R Studio (version 4.1.3) to examine Cox proportional hazards for all possible points of separation (low–high cut-points), selecting the cut-point with the lowest *p*-value [52]. Therefore, patients were divided according to high and low expression levels of specified genes. The Kaplan Meier survival curves were generated using the survival and the survminer packages in R Studio.

### Enrichment and correlation analyses

In order to investigate the biological significance of the DEGs arising from the RNA-seq analysis, which display a log2FC ≥ 0.5 and *p*-value < 0.05, gene ontology (GO) analysis was performed employing the *gseGO()* function of the topGO package [53] in R Studio. *p*-value ≤ 0.01 was considered as a significant threshold. The Pearson correlation coefficients (*r*-values) between the expression levels of EphA3 and the other genes of the TCGA (n. 20,530) dataset were assessed in R Studio using the *cor.test()* function and setting the method as “Pearson”. The statistical significance of the correlation coefficients was calculated by the t-test, *p* < 0.001 was considered as a cut-off criterion. The first 1000 positively correlated genes were selected for the next evaluations. In particular, aiming to cluster these genes in pathways and GO terms, we uploaded our list on the Database for Annotation, Visualization and Integrated Discovery (DAVID) functional annotation analysis website (https://david.ncifcrf.gov/). We grouped the genes in pathways by the option Kyoto Encyclopedia of Genes and Genomes (KEGG); furthermore, we clustered the genes by the Gene Ontology tool and the options GOTERM_BP, GOTERM_MF, GOTERM_CC. In both analyses the official gene symbol as “select identifier” and gene list as “list type” were chosen in the options of the upload and a limit species of “Homo sapiens” in the background was selected.

### Gene silencing experiments

Silencing of EphA3 and RAGE was performed by transiently transfecting the cells for 36 h using Lipofectamine RNAiMAX (Thermo Fisher Scientific). Cells were seeded in six-well multi-dishes and transiently transfected after 24 h with a pool of three unique 27mer siRNA duplexes targeting-sequences (siEPHA3 or siAGER) or a non-targeting scramble control (OriGene Technologies, DBA, Milan, Italy).

### Gene expression studies

Total RNA was extracted, and cDNA was synthesized by reverse transcription as previously described [46]. The expression of selected genes was quantified by real-time PCR using platform Quant Studio7 Flex Real-Time PCR System (Thermo Fisher Scientific). Gene-specific primers were designed using Primer Express version 2.0 software (Applied Biosystems) and are as follows: 5′-CAGCGGCAGTAGCAATTATTCTC-3′ (EPHA3 forward) and 5′-CCACAGAACCTCCCAATCAAA-3′ (EPHA3 reverse); 5′-GTGAGAAGGGACTCCGTGTG-3′ (EFNB2 forward) and 5′-TAGACCCCAGAGGTTAGGGC-3′ (EFNB2 reverse); 5′-AAGCCACCCCACTTCTCTCTAA-3′ (ACTB forward) and 5′-CACCTCCCCTGTGTGGACTT-3′ (ACTB reverse). Assays were performed in triplicate and the results were normalized for ACTB expression and then calculated as fold induction of RNA expression.

### Chromatin Immunoprecipitation (ChIP) assay

Cells were grown in 10-cm dishes, then cross-linked with 1% formaldehyde and sonicated. Supernatants were immuno-cleared with salmon DNA/protein A-agarose (Merck Life Science) and immunoprecipitated with anti-Sp1 primary antibody (1C6, Santa Cruz Biotechnology) or nonspecific IgG. Pellets were washed, eluted with a buffer consisting of 1% SDS and 0.1 mol/L NaHCO3, and digested with proteinase K. DNA was obtained by phenol/chloroform extractions and precipitated with ethanol. The yield of target region DNA in each sample after ChIP was analyzed by real-time PCR. The primers used to amplify a region containing a Sp1 site located into the EphA3 promoter sequence were: 5′-CAAACTTGACATCAGCCTGCG-3′ (forward) and 5′-TCTCCATGAAGCATGCCACT-3′ (reverse). Data were normalized to the input for the immunoprecipitation and the results were reported as fold changes with respect to nonspecific IgG. The Sp1 sites within the EphA3 promoter were identified using the program “AliBaba2.1”, which is a specific tool for predicting binding sites of transcription factors in an unknown DNA sequence by constructing matrices on the fly from TRANSFAC 4.0 sites.

### Flow cytometry analysis

Cells (2 × 10^6^) were fixed in ice-cold methanol for 10 min, permeabilized in 0.1% Triton X-100 in PBS for 15 min and incubated with primary antibody anti-RAGE (MAB1145, R&D Systems) for 1 h at 4 °C. Alexa Fluor 488-conjugated secondary antibody (Thermo Fisher Scientific) was subsequently applied for another 30 min in the dark. Samples were then analyzed with CytoFLEX flow cytometry (Beckman-Coulter, Milan, Italy).

### Western blot analysis

Cells were grown in 10-cm dishes, exposed to treatments where required and then lysed as previously described [54]. Equal amounts of whole-protein extract were resolved on a 8–10% SDS–polyacrylamide gel and transferred to a nitrocellulose membrane (Amersham Biosciences, Merck Life Science), which was probed with primary antibodies against RAGE (MAB1145, R&D Systems, 1:1000), EphA3 (12,480–1-AP, Proteintech, 1:500), SP1 (1C6, Santa Cruz Biotechnology, 1:1000) and β-actin (AC-15, Santa Cruz Biotechnology, 1:4000) and then revealed using the Clarity™ Western ECL Substrate (Bio-Rad).

### Immunofluorescence microscopy

Cells were grown on a cover slip, next were fixed in 4% paraformaldehyde in PBS, permeabilized with 0.2% Triton X-100, washed 3 times with PBS and incubated at 4 °C overnight with primary antibodies against RAGE (MAB1145, R&D Systems, 1:500) or Ephrin A3 (12,480–1-AP, Proteintech, 1:100. After incubation, the slides were extensively washed with PBS, probed with Alexa Fluor 488 goat anti-mouse IgG or Alexa Fluor 555 goat anti-rabbit IgG (Thermo Fisher Scientific, 1:250) and 4′,6-diamidino-2-phenylindole dihydrochloride (DAPI) (Merck Life Science, 1:1000). Images were obtained using the EVOS™ M7000 Imaging System (Thermo Fisher Scientific) and analyzed using ImageJ software by selecting one cell at a time in each picture and measuring the area, integrated density and mean gray value. Thereafter, the corrected total cell fluorescence (CTCF) = integrated density—(area of selected cell × mean fluorescence of background readings) was calculated. Box plots and statistical analysis (t-tests) were performed using R Studio.

### Scanning electron microscopy

Cells were fixed in 3% glutaraldehyde in 0.1 M phosphate buffer (pH 7.2) for 2 h at 4 °C, washed three times with 0.1 M phosphate buffer, pH 7.2, and post-fixed in 1% osmium tetroxide in the same buffer. After 2 h, the samples were dehydrated using increasing concentrations of ethanol and dried with hexamethyldisilazane. Finally, glass slides were mounted on standard aluminum stubs with double-sided conductivity tape, coated with carbon, and observed at various magnifications using the Ultra High Resolution SEM (UHR-SEM) – ZEISS CrossBeam 350.

### Colony formation assay

Cells were cultured in regular growth medium to 90% confluence. Cells were then trypsinized, counted and seeded (3 × 10^3^) in six-well plates in medium containing 2.5% charcoal-stripped FBS. Media were renewed every 2 days. After 10 days, cells were washed with PBS, fixed in acetone:methanol (1:1) for 3 min at room temperature and then stained with Giemsa for 10 min. A total of 10 pictures for each condition was detected using a digital camera and colony number (more than 40 cells) was measured by ImageJ program.

### Migration and invasion assays

Transwell 8 μm polycarbonate membranes (Costar, Merck Life Science) were used to evaluate in vitro cell migration and invasion. 5 × 10^4^ cells (previously transfected where required) in 300 μL serum-free medium were seeded in the upper chamber coated with (invasion assay) or without (migration assay) Corning® Matrigel® Growth Factor Reduced (GFR) Basement Membrane Matrix (Merck Life Science) (diluted with serum-free medium at a ratio of 1:3). Medium containing 2.5% FBS and treatments was added to the bottom chambers. 18 h after seeding, cells on the upper surface of the membrane were then removed by wiping with Q-tip, and migrated or invaded cells were fixed with 100% methanol, stained with Giemsa (Merck Life Science), photographed using an inverted phase contrast microscope and counted using the WCIF ImageJ software.

### Co-culture Matrigel drops evasion assay

The Matrigel drop assay was performed as previously described [55, 56]. 5 × 10^4^/drop of MCF7/wt or MCF7/RAGE cells and CAFs (ratio 2:1) were gently mixed with 10 μl of Corning® Matrigel® Growth Factor Reduced (GFR) Basement Membrane Matrix (Merck Life Science). The cell/matrigel suspension was layered onto the surface of 24-well plate to form a well-defined drop and placed at 37 °C to solidify. Medium containing vehicle or 10 μM AWL-II-38.3 was then placed over the drop. Cells were observed at specified time points and drops were photographed using the EVOS™ M7000 Imaging System (Thermo Fisher Scientific). The migrating edge outside the Matrigel drop was measured with ImageJ software.

### Zebrafish maintenance and egg collection

Wild-type adult zebrafish (6–8 months) of both sexes were purchased from a local store and maintained in 100 L aquaria filled with aged tap water (temperature 28 ± 0.5 °C, pH 7.3, conductivity 300 L/cm, dissolved oxygen 8 ± 1 mg/L, hardness 180 mg, 14 h light/10 h dark photoperiod). Embryos obtained from the spontaneous spawning of adult fish were collected within 2 h and staged as previously reported [57]. Fertilized healthy embryos were maintained in Petri dishes containing aged tap water in a 28.5 °C incubator.

### Zebrafish microinjections

At 48 h post-fertilization (hpf), dechorionated larvae were anesthetized with tricaine methanesulphonate (MS-222) and positioned on a modified Petri dish coated with 1.5% w/v agarose. Before injection, cells were labeled in vitro with CellTracker™ CM-DiI Dye (Thermo Fisher Scientific). Approximately 250 cells were resuspended in a complete medium, and 33.51 nL of cell solution were injected using commercially available ready-to-use tip needles (15 μm inner diameter, Eppendorf, Germany) into the yolk sac. An Eppendorf’s semi-automated microinjection system equipped with the micromanipulator InjectMan 4 connected to FemtoJet 4 × was used for the injections, with an injection pressure of 100 hPa, 0.2 s injection time and 20 hPa of compensation pressure. After injections, the larvae were immediately transferred into housing-keeping water. Injected larvae were kept at 28 ± 0.5 °C and examined every other day for monitoring tumor growth and invasion using the EVOS™ M7000 Imaging System (Thermo Fisher Scientific).

### Statistical analysis

All bioinformatics analyses were carried out using R Studio. The volcano plots were performed with the tidyverse package. Box plots were performed with the tidyverse package and the related statistical analysis was performed using the Wilcoxon test. Statistical analysis was performed using t-tests.

## Results

### RAGE-dependent invasive gene expression signature of BC cells

**Fig.1:**
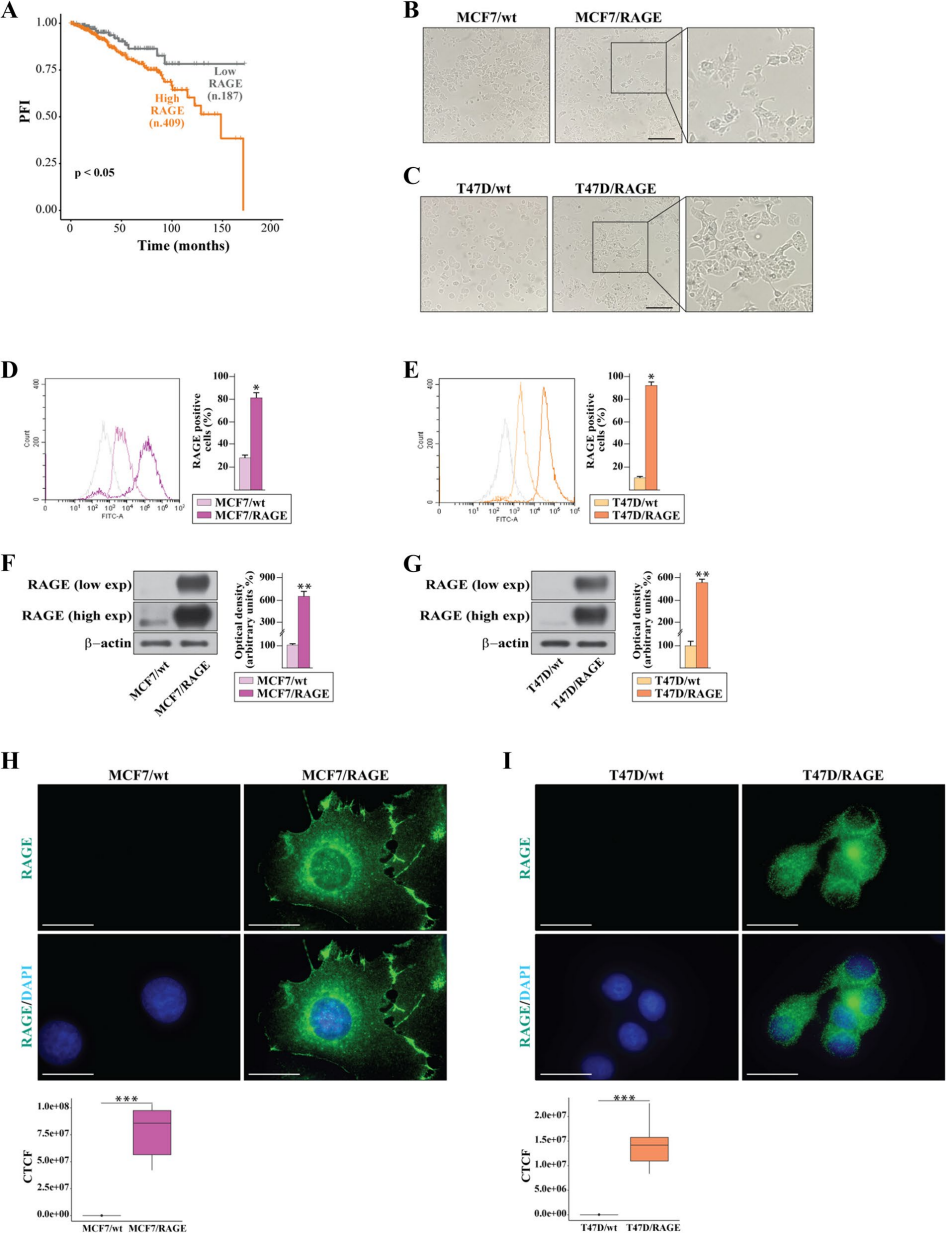
Validation of RAGE-overexpressing BC cells. **A** Kaplan–Meier plot showing the association of RAGE mRNA expression with the progression free interval (PFI) of the TCGA ER-positive BC patients. Samples were divided into RAGE high and low groups using the optimum cut-off. **B**-**C** Morphological appearance of wild type (MCF7/wt and T47D/wt) and RAGE-overexpressing (MCF7/RAGE and T47D/RAGE) cells in phase-contrast microscopy; scale bar: 250 μm. Enlarged details are shown in the separate boxes. Flow cytometric histograms in RAGE-overexpressing compared with wild type MCF7 (**D**) and T47D (**E**) cells. FITC, fluorescein isothiocyanate. Side panels show the percentage of RAGE-positive cells. **F**-**G** Immunoblots of RAGE in wild type (MCF7/wt and T47D/wt) and RAGE-overexpressing (MCF7/RAGE and T47D/RAGE) cells. Side panels show densitometric analysis of the blots normalized to β-actin, which was used as a loading control. Values represent the mean ± SD of three independent experiments performed in triplicate. **H**-**I** Evaluation of RAGE protein expression (green signal) by immunofluorescence experiment in wild type (MCF7/wt and T47D/wt) and RAGE-overexpressing (MCF7/RAGE and T47D/RAGE) cells; nuclei were stained by DAPI (blue signal). The images shown represent 10 random fields from three independent experiments. Scale bar: 25 μm. Side panels represent corrected total cell fluorescence (CTCF), which was calculated on at least 10 pictures from each sample. (*) indicates *p* < 0.05; (**) indicates *p* < 0.01; (**) indicates *p* < 0.001

**Fig.2:**
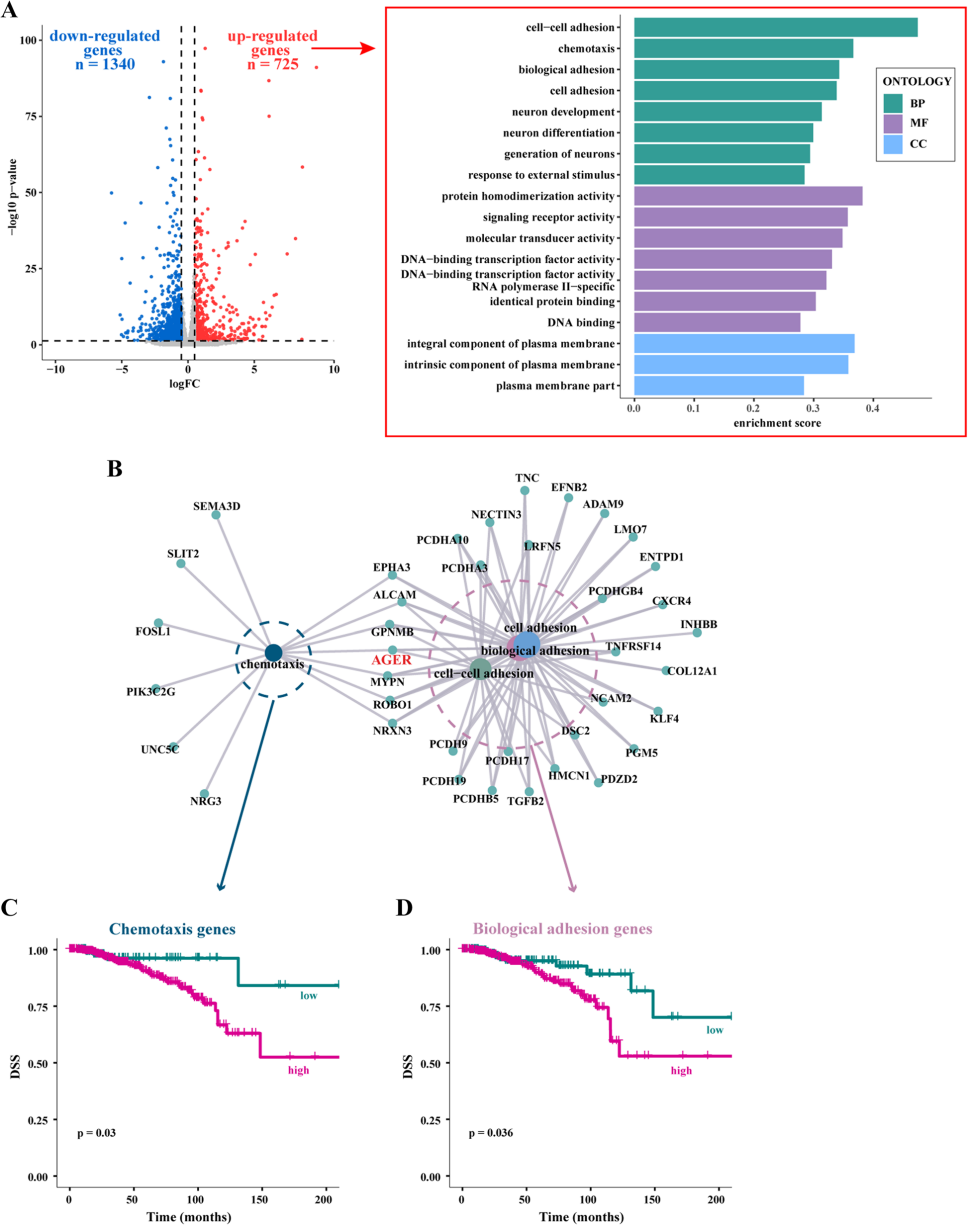
Comprehensive transcriptome analysis by RNA-seq of wild type and RAGE-overexpressing MCF7 cells. **A** Volcano plot evidencing the differentially expressed genes (DEGs) in RAGE-overexpressing (MCF7/RAGE) respect to wild type (MCF7/wt) MCF7 cells, as ascertained by RNA-seq analysis. p-value < 0.05 was considered as a significant threshold; significantly down-regulated genes (log2FC ≤ 0.5 and *p* < 0.05) are shown in blue (*n* = 1340), significantly up-regulated genes (log2FC ≥ 0.5 and *p* < 0.05) are shown in red (*n* = 725), non-significant genes are shown in grey (*p* > 0.05). Side box shows enrichment scores for gene ontology (GO) terms of the up-regulated DEGs in MCF7/RAGE versus MCF7/wt cells, involved in biological process (BP), molecular function (MF), cellular component (CC), according to *p* < 0.05 and log2FC > 0.5. **B** Interrelation analysis of “cell–cell adhesion”, “chemotaxis”, “biological adhesion” and “cell adhesion” BP and relative genes. Survival analysis showing the association of high expression of the genes belonging to the “chemotaxis” (**C**) and “biological adhesion” (**D**) BP with a worse disease specific survival (DSS) in ER-positive BC patients of the TCGA cohort

On the basis of previous evidence showing the involvement of RAGE in BC progression [25, 30], we began our study exploring its clinical significance in the cohort of BC patients of the TCGA database. High expression levels of RAGE were found to correlate with a worse progression free interval (PFI) in ER-positive (Fig. 1A) but not in ER-negative (data not shown) BC patients. Therefore, in order to provide novel insights on the role of RAGE in ER-positive BC, we engineered MCF7 and T47D BC cells to stably overexpress RAGE (MCF7/RAGE and T47D/RAGE cells), as detailed in the methods section. Microscopic evaluation showed that both MCF7/RAGE and T47D/RAGE cells exhibit an evident spindle-shaped phenotype with membrane protrusions compared to MCF7 and T47D wild type (MCF7/wt and T47D/wt) cells (Fig. 1B-C). Flow cytometry analyses (Fig. 1D-E), immunoblotting experiments (Fig. 1F-G) and immunofluorescent staining (F ig. 1H-I) revealed increased RAGE protein levels in MCF7/RAGE and T47D/RAGE cells. Therefore, we performed whole transcriptome RNA sequencing (RNA-seq) analysis of MCF7/wt and MCF7/RAGE cells to appreciate the global gene expression networks regulated by RAGE. Figure 2 (panel A) shows the volcano plot portraying the differentially expressed genes (DEGs) in MCF7/RAGE respect to MCF7/wt cells. In particular, 725 genes were found up-regulated (log2FC ≥ 0.5, *p* < 0.05) (Additional File 1) and 1340 genes were found down-regulated (log2FC ≤ -0.5, *p* < 0.05) (Additional File 2) in MCF7/RAGE respect to MCF7/wt cells. Aiming to assess the biological significance of the up-regulated DEGs in RAGE-overexpressing BC cells, we performed gene ontology (GO) analysis clustering these genes according to biological process (BP), molecular function (MF) and cellular component (CC) terms. The most significantly enriched BP terms were “cell–cell adhesion”, “chemotaxis”, “biological adhesion” and “cell adhesion” (Fig. 2A, side box). It is worth noting that RAGE (AGER, gene symbol) belongs to the genes listed into all the aforementioned BP. Thereafter, we took advantage of the TCGA dataset in order to ascertain whether the genes of the aforementioned BP terms and highlighted in Fig. 2 (panel B) may have a prognostic value in ER-positive BC patients. To avoid overlapping results, for the subsequent analyses we used the “biological adhesion” set as it encompasses all the genes belonging to the “cell–cell adhesion” and “cell adhesion” terms. Kaplan–Meier survival plots revealed that the cumulative expression of the chemotaxis-related genes (Fig. 2C) or the biological adhesion genes (Fig. 2D) is associated with a worse disease specific survival (DSS) in ER-positive BC patients. Overall, these data suggest that increased RAGE levels might guide a transcriptional program associated with BC cell motility toward a likely adverse prognostic shift.

### RAGE prompts BC cell dissemination

**Fig.3:**
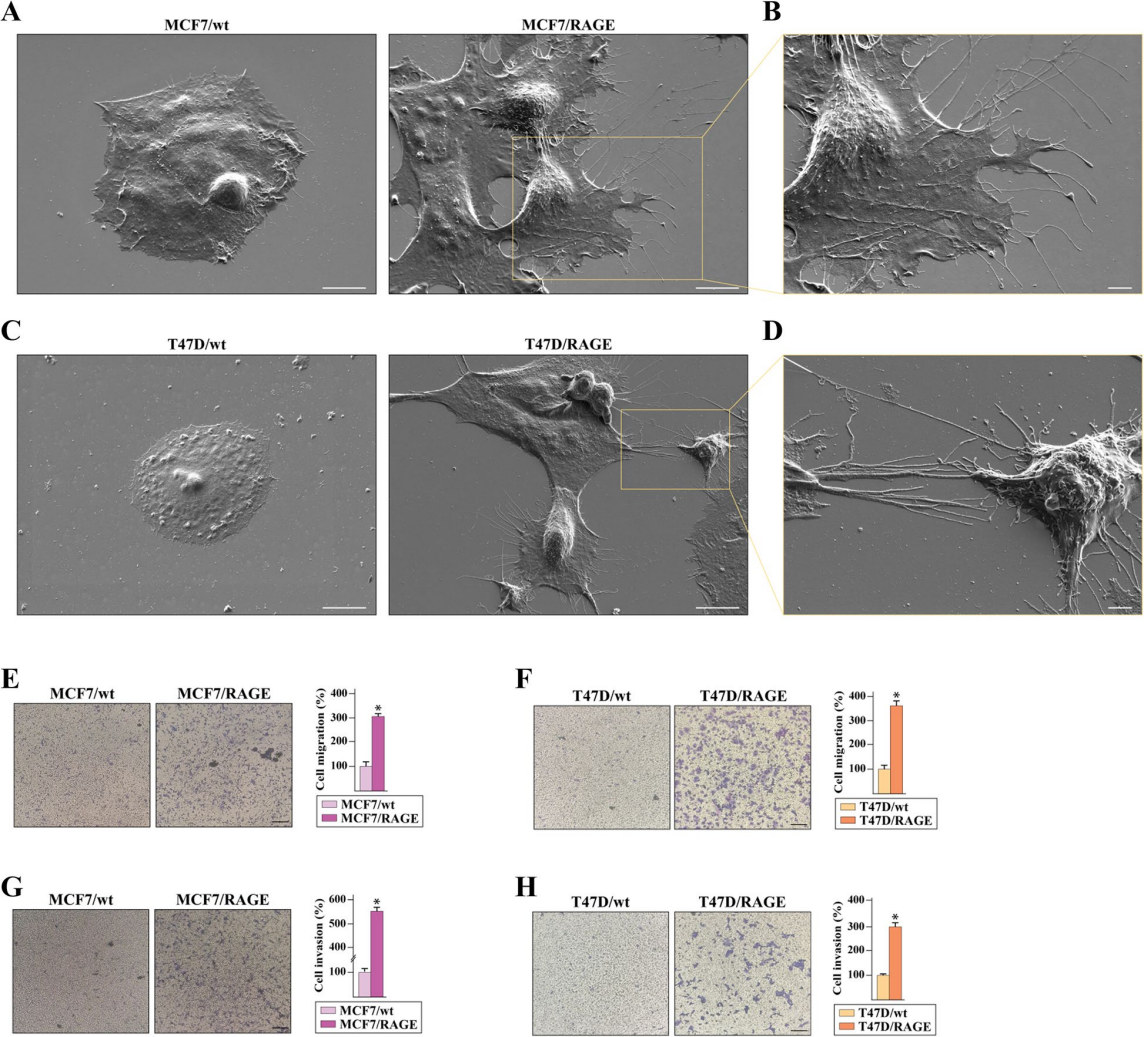
Membrane protrusions and motile phenotype in RAGE-overexpressing BC cells. **A** Scanning electron microscopy (SEM) images of wild type (MCF7/wt) and RAGE-overexpressing (MCF7/RAGE) MCF7 cells; scale bar: 10 μm. **B** Higher magnification (square in A), scale bar: 3 μm. **C** SEM images of wild type (T47D/wt) and RAGE-overexpressing (T47D/RAGE) T47D cells; scale bar: 10 μm. **D** Higher magnification (square in C), scale bar: 2 μm. Transwell migration (**E**–**F**) and invasion (**G**-**H**) assays in wild type and RAGE-overexpressing MCF7 and T47D cells. Cells were counted in at least 5 random fields in three independent experiments performed in triplicate, as quantified in the side panels. Scale bar 200 μm

**Fig.4:**
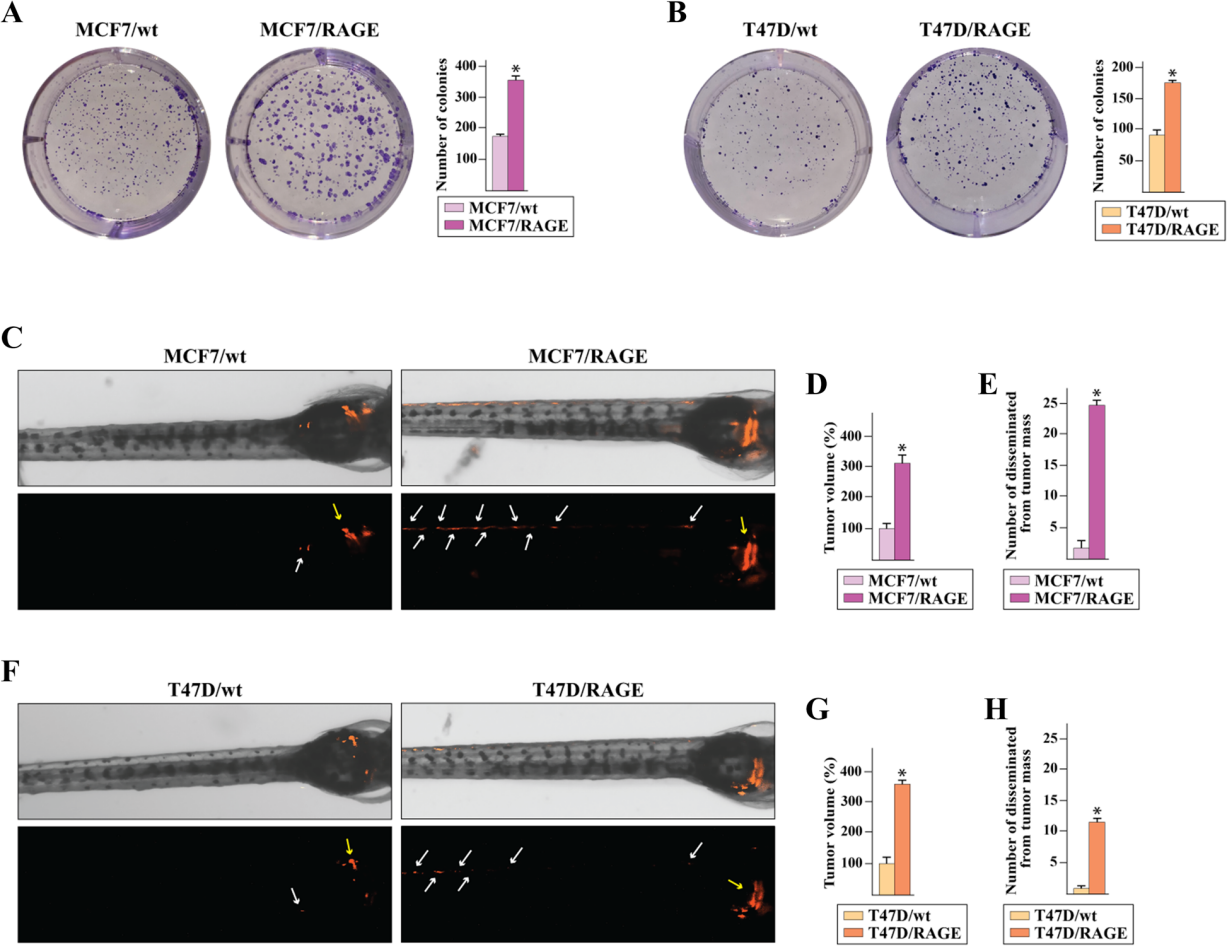
Growth and dissemination of RAGE-overexpressing BC cells. **A**-**B** Colony formation in wild type (MCF7/wt and T47D/wt) and RAGE-overexpressing (MCF7/RAGE and T47D/RAGE) MCF7 and T47D cells. After 10 days of incubation cell colonies were stained and pictures were captured by a digital camera. Colonies were counted using the program WCIF ImageJ for Windows. Each data point is the mean ± SD of three independent experiments performed in triplicate. **C**,**F** DiI-labeled wild type (MCF7/wt and T47D/wt) and RAGE-overexpressing (MCF7/RAGE and T47D/RAGE) cells were injected into the yolk sac of 48 hpf larvae, and tumor cell growth and dissemination were detected using fluorescent microscopy at day 3 post-injection. Yellow arrows indicate primary tumors. White arrows indicate disseminated tumor foci. **D**,**G** Quantification of DiI-labeled tumor volume (*n* = 6/group). The mean value of the tumor size in wild type zebrafish xenografts was settled as 100%. **E**,**H** Quantification of numbers of disseminated tumor foci (*n* = 6/group). Data are represented as mean ± SD. (*) indicates *p* < 0.05

To address the role of RAGE-driven gene signature in the outcome of BC cells, morphological and functional studies were performed. As shown in Fig. 3 (panels A-D), the increased RAGE expression stimulates cytoplasmic protrusions in MCF7/RAGE and T47D/RAGE cells. In particular, scanning electron microscopy (SEM) investigation revealed that both long and short lengths filopodia extend from the entire circumference of the cell body and in some cases appear as bridge-like protrusions serving for intercellular connections (Fig. 3A-D). On the basis of these findings and in accordance with previous evidence indicating that filopodial protrusions are abundant at the invasive front of cancer cells participating in cellular motility [58, 59], we found that MCF7/RAGE and T47D/RAGE cells display higher migratory (Fig. 3E-F) and invasive (Fig. 3G-H) potential compared to MCF7/wt and T47D/wt cells, respectively. Remarkably, the silencing of RAGE by siRNA impaired the migratory and invasive behavior of RAGE-overexpressing BC cells (Additional File 3), supporting the involvement of RAGE in inducing motile features in BC cells. To further investigate the pro-tumorigenic properties of RAGE overexpressing BC cells, we evaluated their ability to undergo “unlimited” division by performing colony formation assays. Of note, the clonogenic formation potential of RAGE-overexpressing MCF7 and T47D cells was found significantly increased respect to the parental cells (Fig. 4A-B). To further extend these data in vivo and to ascertain whether RAGE may also have a role in promoting early events of the metastatic cascade, cells were labeled with DiI dye and were injected into the yolk sac of 48 hpf larvae. At day 3 after implantation, tumor growth increased in the zebrafish xenotransplanted with RAGE-overexpressing cells respect to wild type zebrafish xenografts (Fig. 4C-D,F-G). Interestingly, MCF7/RAGE (Fig. 4C,E) and T47D/RAGE (Fig. 4F,H) cells disseminated into the dorsal stripe, while MCF7/wt and T47D/wt xenotransplanted groups did not show significant evidence of metastases (Fig. 4C,E,F,H), in accordance with previous studies showing that MCF7 and T47D cells do not typically invade or metastasize [60, 61].

### EphA3-mediated signaling is involved in the RAGE-dependent migration and invasion of BC cells

**Fig.5:**
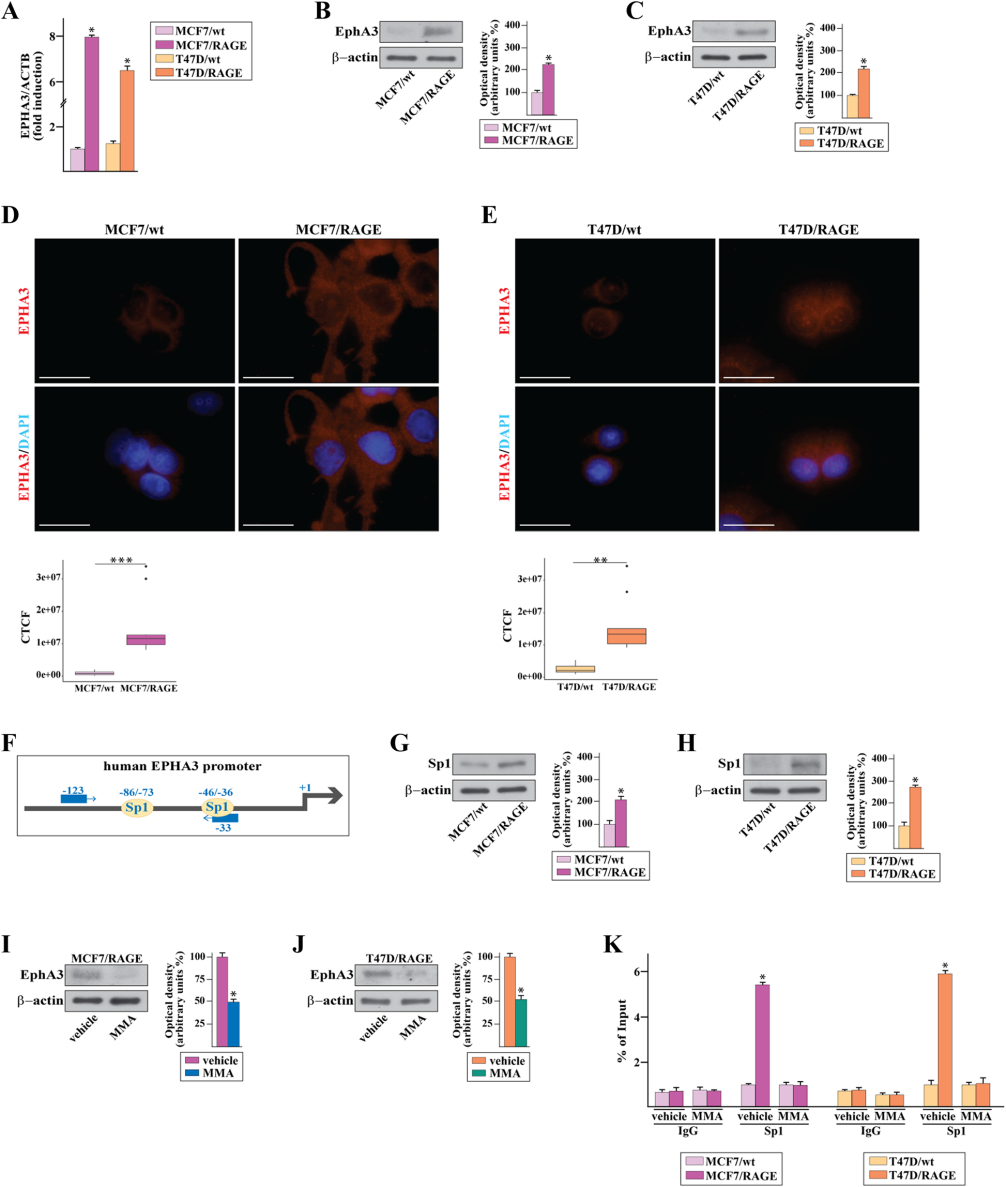
Sp1 is involved in the up-regulation of Epha3 in RAGE-overexpressing BC cells. **A** mRNA expression of EPHA3 in RAGE-overexpressing (MCF7/RAGE and T47D/RAGE) respect to wild type (MCF7/wt and T47D/wt) cells, as ascertained by real-time PCR. Values are normalized to the actin beta (ACTB) expression and shown as fold changes of mRNA expression in RAGE-overexpressing respect to wild type cells. **B**-**C** Immunoblots of EphA3 in wild type (MCF7/wt and T47D/wt) and RAGE-overexpressing (MCF7/RAGE and T47D/RAGE) cells. Side panels show densitometric analysis of the blots normalized to β-actin, which was used as a loading control. **D**-**E** Evaluation of EphA3 protein expression (red signal) by immunofluorescence experiment in wild type (MCF7/wt and T47D/wt) and RAGE-overexpressing (MCF7/RAGE and T47D/RAGE) cells; nuclei were stained by DAPI (blue signal). The images shown represent 10 random fields from three independent experiments. Bottom panels represent corrected total cell fluorescence (CTCF), which was calculated on at least 10 pictures from each sample. Scale bar: 25 μm. **F** Schematic representation of human EPHA3 promoter carrying the Sp1-responsive sites (the transcriptional start site is indicated as + 1). **G**-**H** Protein expression of Sp1 in wild type (MCF7/wt and T47D/wt) and RAGE-overexpressing (MCF7/RAGE and T47D/RAGE) cells, as evaluated by immunoblotting. **I**-**J** Protein levels of EphA3 in the presence of 100 nM Sp1 inhibitor mithramycin A (MMA) in RAGE-overexpressing (MCF7/RAGE and T47D/RAGE) cells. Side panel shows densitometric analysis of the blots normalized to β-actin. **K** Recruitment of Sp1 to EphA3 promoter by ChIP assay in wild type (MCF7/wt and T47D/wt) and RAGE-overexpressing (MCF7/RAGE and T47D/RAGE) cells in the presence or absence of 100 nM Sp1 inhibitor mithramycin A (MMA). In control samples nonspecific IgGs were used instead of the primary antibody. The amplified sequences were evaluated by real-time PCR. Values represent the mean ± SD of three independent experiments performed in triplicate. (*) indicates *p* < 0.05

On the basis of the aforementioned data, we aimed at providing mechanistic insights on the molecular events through which RAGE may drive BC growth and metastatic spread. As revealed by our RNA-seq study, EphA3 is one of most up-regulated genes in MCF7/RAGE respect to MCF7/wt cells and belongs to both biological adhesion and chemotaxis terms of GO analysis. EphA3 is a member of the Eph receptor tyrosine kinases that regulate the morphology, adhesion, migration and invasion of cancer cells in vitro as well as tumor growth, invasiveness, angiogenesis and metastasis in vivo [62]. Consistent with the RNA-seq data, we first confirmed that RAGE-overexpressing BC cells display higher EphA3 mRNA (Fig. 5A) and protein (Fig. 5B-E) levels than wild type cells. To provide mechanistic insights into EphA3 regulation in RAGE-overexpressing BC cells, we investigated the role of Sp1 in EphA3 modulation considering the Sp1 up-regulation assessed in MCF7/RAGE compared to MCF7/wt cells. In this regard, we first ascertained that the EphA3 promoter region, located most proximal to the start codon, contains three Sp1 sites. Therefore, specific primers for two out of these three Sp1 sites were designed (Fig. 5F). Next, we demonstrated that Sp1 levels are up-regulated in MCF7/RAGE and T47D/RAGE respect to parental cells (Fig. 5G-H). Remarkably, the Sp1 inhibitor mithramycin A (MMA) decreased the protein levels of EphA3 in RAGE-overexpressing BC cells (F ig. 5I-J), further suggesting that Sp1 can be involved in the regulation of EphA3, as previously reported [63]. Accordingly, we found that Sp1 is recruited to the EphA3 promoter region in both MCF7/RAGE and T47D/RAGE cells, as ascertained by chromatin immunoprecipitation (ChIP) assay (Fig. 5K). Further corroborating the role of the Sp1 sites in the RAGE-induced expression of EphA3, the Sp1 inhibitor MMA prevented the recruitment of Sp1 to the Sp1 DNA binding sites located within the EphA3 promoter (Fig. 5K).

**Fig.6:**
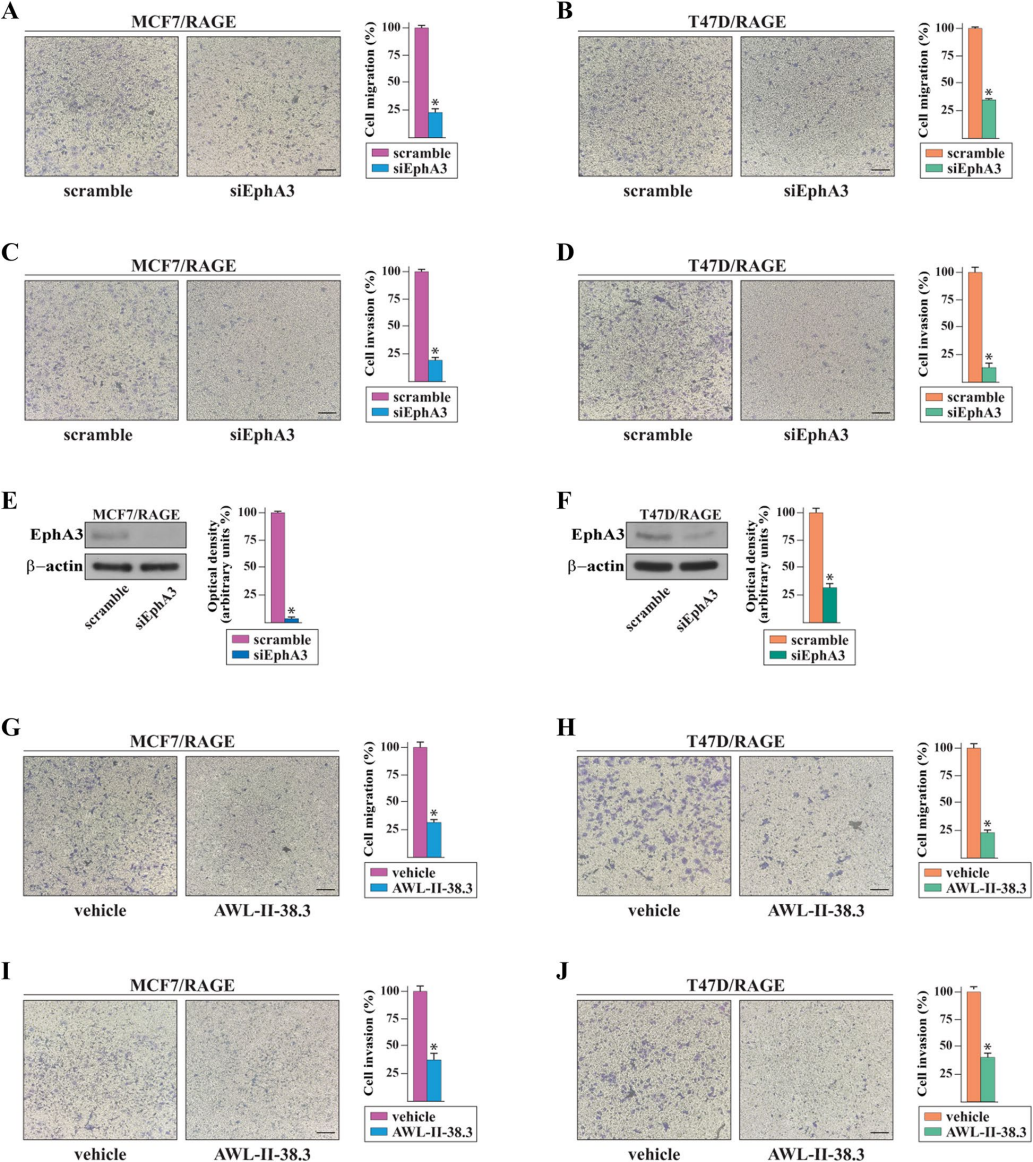
The EphA3 signaling mediates the motile phenotype of RAGE-overexpressing BC cells and is associated with a worse prognosis in BC patients. Transwell migration (**A**-**B**) and invasion (**C**-**D**) assays in MCF7/RAGE and T47D/RAGE cells in the presence or absence of EphA3 silencing. Efficacy of EphA3 silencing in MCF7/RAGE (**E**) and T47D/RAGE (**F**) cells. Side panel shows densitometric analysis of the blots normalized to β-actin. Transwell migration (**G**-**H**) and invasion (**I**-**J**) assays in MCF7/RAGE and T47D/RAGE cells in the presence or absence of 10 μM EphA3 inhibitor AWL-II-38.3. Cells were counted in at least 5 random fields in three independent experiments performed in triplicate, as quantified in the side panels. Scale bar: 200 μm. Values represent the mean ± SD of three independent experiments performed in triplicate. (*) indicates *p* < 0.05

The complexes between Eph receptors and their ligands, named ephrins, typically emanate the following bidirectional signals: i) forward signals that rely on Eph kinase activity and propagate to the receptor-expressing cells; ii) reverse signals that depend on Src kinase family and propagate to the ligand-expressing cells [62]. On the basis of these observations and in accordance with RNA-seq data, we first confirmed by real-time PCR that MCF7/RAGE and T47D/RAGE cells express high levels of the EphA3 ligand namely Ephrin B2 (Additional File 4). Next, we ascertained that the migration and invasion of MCF7/RAGE and T47D/RAGE cells occur in an EphA3-dependent manner given that the silencing of EphA3 expression abrogated these responses (Fig. 6A-F). Interestingly, the EphA3 kinase inhibitor AWL-II-38.3, which is not able to modify the protein levels of EphA3 (Additional File 5), reduced the migratory (Fig. 6G-H) and invasive (F ig. 6I-J) capacities of MCF7/RAGE and T47D/RAGE cells, suggesting that the kinase-dependent EphA3 forward signaling may be implicated in the motility of RAGE-overexpressing BC cells.

**Fig.7:**
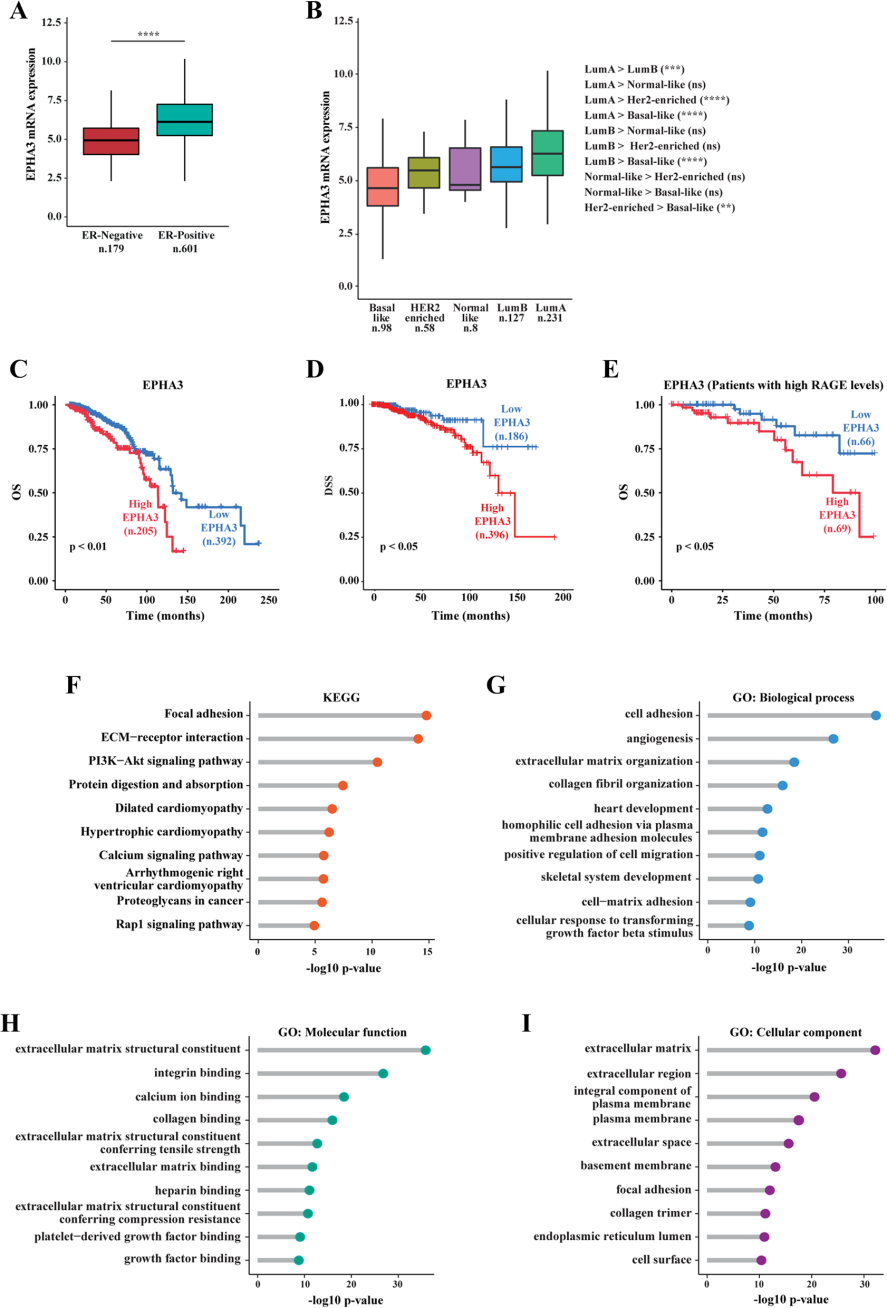
EphA3 expression correlates with poor clinical outcomes and pro-migratory and invasive genes in ER-positive BC patients. **A** Box plot showing the differential EphA3 expression levels in ER-positive and negative BC patients, as found in the TCGA dataset. **B** EphA3 mRNA levels according to BC intrinsic molecular subtypes of the TCGA cohort. The number of patients is reported in each panel. Kaplan–Meier plots showing the association of EphA3 expression with the overall survival (OS) (**C**) and disease specific survival (DSS) (**D**) of the TCGA ER-positive BC patients. Patients were divided into EphA3 high and low categories using the optimum cut-off. **E** Kaplan–Meier plot showing the association of EphA3 expression with the OS of ER-positive BC patients characterized by high RAGE expression levels (above the 3Q). Patients were divided into EphA3 high and low categories using the optimum cut-off. KEGG pathway (**F**) and gene ontology (GO) (**G**-**I**) analyses depicting the association of EphA3 expression with pro‐metastatic pathways and GO terms in ER‐positive BC samples of TCGA. The x-axes and the y-axes indicate respectively the -log10 *p*-value and the different KEGG pathways and GO terms. Lum A, Luminal A; Lum B, Luminal B; ns, not significant; (**) indicates *p* < 0.01; (***) indicates *p* < 0.001 and (****) indicates *p* < 0.0001

In order to better appreciate the biological significance of EphA3 in breast malignancy, we aimed to analyze its expression in the TCGA cohort of BC samples. In this respect, our analysis revealed that the expression levels of EphA3 are higher in ER-positive than ER-negative BC patients (Fig. 7A) as well as in luminal A and luminal B subtypes respect to the other molecular subgroups (Fig. 7B). In addition, we found that high EphA3 levels are significantly associated with a worse overall survival (OS) (Fig. 7C) and DSS (Fig. 7D) in ER-positive patients. In addition, distributing the patients on the basis of high and low RAGE levels (3Q of RAGE gene expression as threshold), we found that elevated EphA3 expression is predictive of a worse OS also in the BC subgroup of patients displaying high RAGE levels (Fig. 7E). Subsequently, we performed KEGG pathway enrichment and GO analyses in ER-positive BC patients using the top 1000 EphA3-correlated genes, ranked by Pearson correlation coefficients. The EphA3-related genes were found to be associated with certain transduction pathways (Fig. 7F) and GO terms (F ig. 7G-I) widely involved in pro-migratory and invasive properties of BC cells. Overall, our findings indicate that high RAGE levels might contribute to a more aggressive phenotype of BC cells orchestrating the transcription of genes involved in BC cell motility.

### RAGE promotes invasion of BC cells and CAFs through the EphA3 transduction pathway

**Fig.8:**
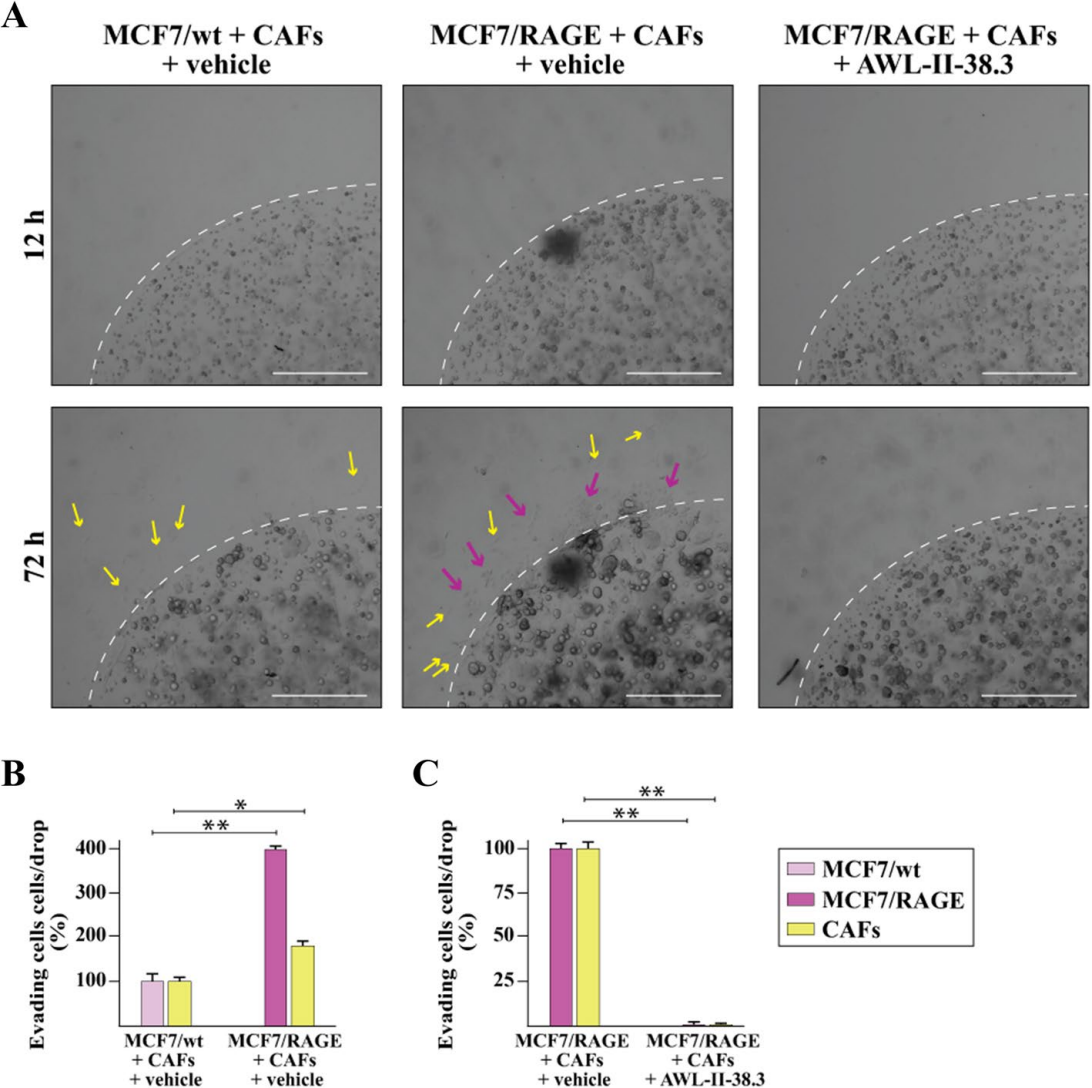
EphA3 axis is involved in the evasion of RAGE-overexpressing BC cells and CAFs from Matrigel drops. **A** Representative phase contrast images from the Matrigel drops evasion assay from co-cultures of MCF7/wt or MCF7/RAGE cells and CAFs in the presence or absence of 10 μM EphA3 inhibitor AWL-II-38.3. **B**-**C** Percentage of cells around the drop upon 3 days treatment from three independent experiments performed in triplicate. Yellow arrows indicate CAFs. Purple arrows indicate MCF7/RAGE cells. The dotted line indicates the border of drop; scale bar: 650 μm. (*) indicates *p* < 0.05; (**) indicates *p* < 0.01

The role of the tumor microenvironment in cancer outcome is widely recognized [64, 65]. In particular, CAFs are key components of the tumor microenvironment prompting the invasion of cancer cells and metastasis through diverse mechanisms including matrix remodeling, production of soluble factors and mutual functional interactions with cancer cells [66]. In this respect, contact-mediated Eph signaling has been shown to influence the migration of tumor cells that can switch from restrained to invasive phenotypes, depending on the Eph-receptor profile of the malignant cells and the reciprocal ephrin ligands expressed by neighboring cells like CAFs [43]. On the basis of these findings, we verified by real-time PCR experiments the mRNA expression of both EphA3 and its ligand Ephrin B2 in CAFs obtained from BC patients (data not shown). Thereafter, in order to assess the role exerted by EphA3-mediated interaction of BC cells and CAFs toward their invasive skills, we performed the 3D matrigel drops evasion assay. In this respect, BC cells and CAFs were first embedded in a drop of matrigel and then exposed to EphA3 kinase inhibitor AWL-II-38.3. Upon this experimental condition, the two cell types were distinguishable for their shape given that BC cells exhibit a spherical morphology, while CAFs show an elongated outline. Fascinatingly, MCF7/wt cells did not exhibit any capacity to migrate contrary to what shown by MCF7/RAGE cells (Fig. 8A-B). Moreover, the EphA3 kinase inhibitor AWL-II-38.3 completely abrogated the migratory potential of both MCF7/RAGE cells and CAFs (Fig. 8A-B), suggesting that the EphA3-mediated signaling is implicated in the motility properties of these cells.

## Discussion

Obesity and T2DM are recognized risk factors for the development of ER-positive BC and are associated with a shorter time to recurrence and greater mortality [5, 6, 8, 9, 67, 68]. Considering that BC is the most common malignancy and the leading cause of cancer death among women [69], a better understanding of the impact of obesity and T2DM on this type of cancer and the underlying molecular mechanistic events may have important implications for patients. Main mechanisms connecting both obesity and T2DM to BC include adipokines, hormones and growth factors (i.e. insulin, insulin-like growth factors and estrogen), hyperglycemia, dyslipidemia, hypoxia-dependent gene transcription, sustained activation of pro-tumorigenic inflammatory mediators and immune function dysregulation [5, 68, 70]. RAGE is aberrantly expressed and activated in obese women and patients affected by T2DM [71–73], therefore has been considered an additional factor connecting obesity/T2DM-related states and BC development [29, 74]. The RAGE ligands, namely AGEs, are formed endogenously or taken up with the diet, accumulate in tissues and circulation of both T2DM and obese patients, and are involved in the progression of several human cancers [17, 71, 75–77]. For instance, high serum levels of AGEs are correlated with enhanced metastasis incidence and mortality among postmenopausal women affected by BC [78, 79]. Moreover, the interaction of AGEs with RAGE initiates intracellular signaling pathways that lead to oxidative stress and sustained inflammatory events toward BC cell growth, angiogenesis, migration, invasion and metastases [78, 80, 81]. Likewise, the activation of RAGE by further ligands, like HMGB1 and S-100 proteins, triggers pro-inflammatory signaling cascades implicated in BC progression [25, 28, 33, 82, 83]. Of note, RAGE levels are low under normal conditions, whereas significantly increase in stressed and damaged tissues, including neoplastic cells. Accordingly, a high expression of RAGE has been found associated with advanced-stage, node-positivity and worse survival in BC [25, 84]. In addition, RAGE is overexpressed in triple-negative BC (TNBC), mediates invasive and metastatic features and correlates with poor prognosis and tumor size in this aggressive BC subtype [28, 30, 84, 85]. As it concerns the ER-positive BC, the molecular and functional role of RAGE remains to be deeply understood. By engineering MCF7 and T47D cells to stably overexpress RAGE, we aimed to provide a comprehensive RAGE-related transcriptomic landscape in ER-positive BC. Genome-wide RNA-seq and GO enrichment analyses indicated that biological events linked to motility may be particularly over-regulated. This is especially the case for the genes belonging to the biological adhesion and chemotaxis processes, which emerged as the most significantly induced in RAGE-overexpressing compared to wild type BC cells.

Both cell adhesion and chemotaxis are acknowledged as essential steps for tumor dissemination in metastatic sites [86–88]. In particular, the intracellular events that direct chemotaxis always encompass chemosensing, polarization and locomotion and result in the generation of protrusions at the leading edge in order to facilitate cell migration [87]. These protrusions are usually driven by actin polymerization and are stabilized by the adhesion to the extracellular matrix (ECM) or adjacent cells. The established adhesions act as traction sites for locomotion because cells move forward over them, thereafter are disassembled allowing cell detachment and migration [87, 89]. In agreement with these data and other studies showing a role for RAGE in the migratory, invasive, EMT-like phenotype of diverse tumors [30, 77, 90–94], we have established that RAGE overexpression leads to a protrusive cell machinery allied to a motile behavior of BC cells both in vitro and in zebrafish xenograft models.

Both cell-to-cell and cell-to-ECM processes involve transmembrane cell adhesion molecules, intracellular scaffold or signaling and cytoskeleton proteins that undergo a dynamic reorganization following certain circumstances like cancer cell migration and metastasis [95]. Cadherins and their associated scaffold proteins, namely catenins, play a major role in cell–cell adhesions, whereas integrins mainly mediate cell-ECM interactions [95, 96]. In addition to the aforementioned adhesion complexes, Eph receptor tyrosine kinases have gained considerable attention as critical adhesion and migration-promoting receptors during normal and oncogenic tissue patterning [35, 36]. To date, the Eph system has been suggested as a feasible and promising target for anticancer therapies [97]. Eph are tyrosine kinase receptors that bind to the so-called membrane-attached protein ephrins. Based on their structural homology, the two subfamilies of Eph, EPHA and EPHB, bind to ephrin-A and ephrin-B ligands, respectively [36]. Considering that both receptors and ligands are membrane-bound, the direct cell–cell interaction is required by the Eph/ephrin system to transduce intracellular signals. Specifically, the binding of a ligand- to a receptor-expressing cell triggers bidirectional transduction events known as ephrin-initiated reverse signaling and Eph-initiated forward signaling, respectively. The resulting pathways orchestrate multiple biological processes including cell survival, proliferation, differentiation, cell–cell and cell-ECM adhesion, motility and invasion [36, 98]. Eph receptors and cognate ligands exhibit different expression patterns and functions in cancer, depending on the stage and the type of malignancy [99]. For instance, in small cell lung cancer (SCLC) a low expression of EphA3 contributes to the development of chemoresistance [100]. On the contrary, in glioblastoma EphA3 is overexpressed and involved in maintaining tumor cells in a less differentiated state [101]. Furthermore, high EphA3 expression is associated with metastasis, advanced stages and worse survival in gastric, colorectal and hepatocellular carcinoma [102–104]. As it concerns BC, EphA3 is highly expressed in lymph node metastases and promotes invasive skills of BC cells [105, 106]. Accordingly, it has been reported that targeting EphA3 prevents BC growth by disrupting the integrity and function of newly formed tumor stroma and microvasculature [105]. Nevertheless, the precise oncogenic expression pattern of EphA3 and its role in BC remain to be clarified. In order to provide novel insights into the capability of RAGE to promote BC aggressive features, we have ascertained that RAGE-overexpressing BC cells exhibit high EphA3 levels, as confirmed by different experimental assays beyond RNA-seq analysis. In accordance with these findings and taking into consideration the pro-invasive action of both RAGE and EphA3 in BC [30, 106], we have also demonstrated that RAGE overexpression facilitates a migratory and invasive phenotype through the activation of the Ephrin B2/EphA3 signaling in ER-positive BC cells. In addition, we have identified the transcription factor Sp1 as a main positive regulator of the EphA3 transcription. In the framework of these observations, we have indeed ascertained that elevated Ep hA3expression may be predictive of a worse prognosis in ER-positive BCs. Furthermore, our data have revealed that the gene signature of BC patients showing high EphA3 levels is indicative of a migratory and invasive behavior. Overall, our findings indicate that high levels of RAGE may contribute to an aggressive phenotype of BC cells through the regulation of genes mainly involved in BC cell motility.

Within the tumor microenvironment (TME), cancer cells coexist and cooperate with a variety of cellular and extracellular components including CAFs, endothelial cells, pericytes, immune/inflammatory cells, and non-ECM components like collagen, fibronectin and laminin [107–109]. Acting as major players in the TME, CAFs actively participate in the development and progression of many types of tumors, including BC [64]. In particular, CAFs regulate cancer cell invasion and metastasis by releasing soluble factors as well as modulating genes implicated in cell adhesion, stress fiber formation, cytoskeletal remodeling and epithelial-mesenchymal transition [110]. Moreover, increasing evidence highlights a direct interaction between CAFs and cancer cells toward cancer cell invasion [111–113]. Nevertheless, the molecular mechanisms driving the physical communication between CAFs and malignant cells are still not well recognized. In an effort to understand the potential contribution of the EphA3 system in the cross-talk among tumor and stromal cells, we performed 3D co-culture motility assays using BC cells and CAFs as model systems. Of note, our data demonstrated that the bidirectional Ephrin B2/EphA3 signaling may trigger invasive properties in both cell types. Therefore, the EphA3-mediated cooperation between BC cells and CAFs may provide a rationale for further investigations aimed at targeting EphA3 in diverse elements within the TME, in order to prevent BC invasion and metastatic scattering.

## Conclusions

**Fig.9:**
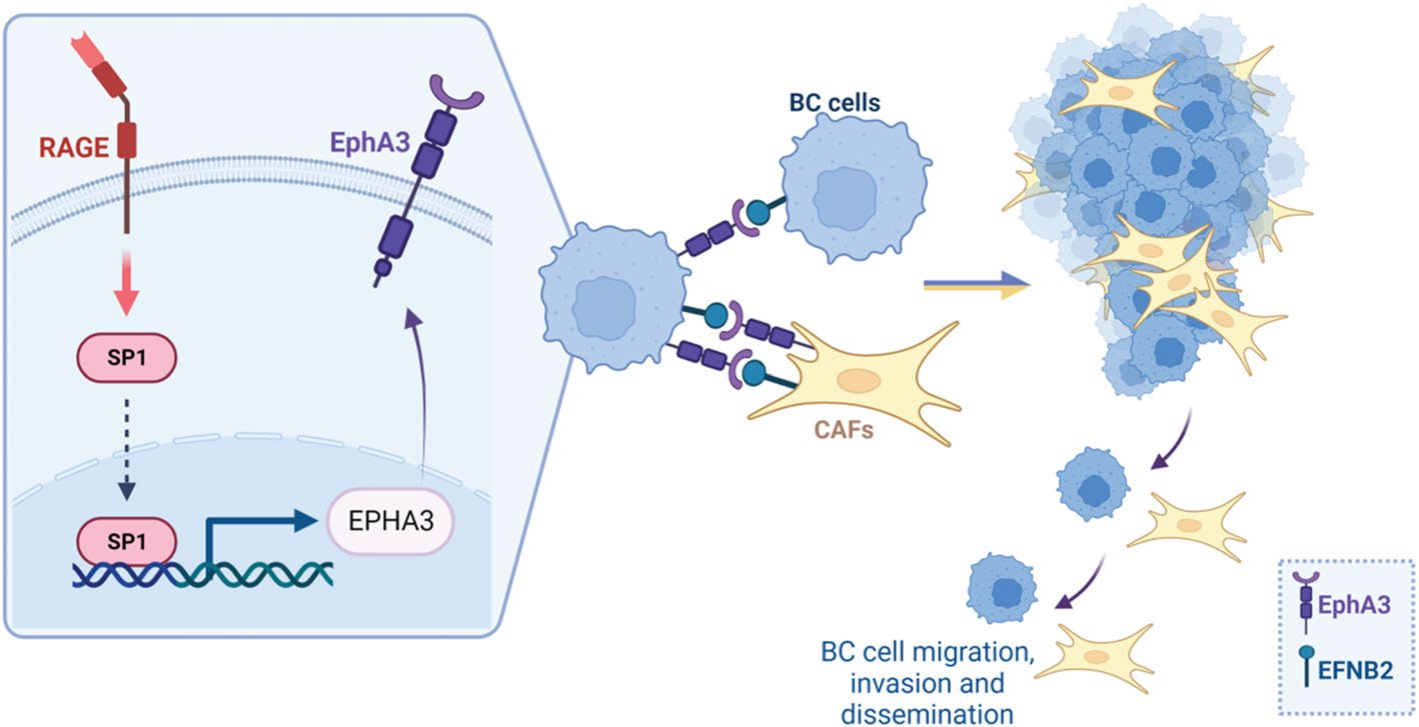
Cartoon depicting the molecular events and the biological responses triggered by RAGE within the BC microenvironment. Created with BioRender.com

Our study uncovers novel transcriptional and molecular mechanisms whereby RAGE may contribute to invasive and metastatic features in ER-positive BC. In particular, high RAGE levels can drive a motile BC behavior by engaging a migratory-related gene signature, including in particular EphA3 and its ligand EFBN2. Furthermore, the RAGE-dependent Ephrin B2/EphA3 transduction signaling may serve as a physical bridge between BC cells and CAFs to drive BC progression (Fig. 9). Taken together, our data suggest that RAGE overexpression, which frequently occurs in diabetic and obese individuals, may have an important role in BC progression. Thus, a comprehensive understanding of the expression profile and biological responses headed by RAGE in ER-positive BC might provide valuable benefits for the assessment of novel therapeutic approaches halting tumor progression, particularly in obese and/or diabetic patients.

## Supplementary Information

**Additional file 1:** Up-regulated genes (log2FC ≥ 0.5, *p* < 0.05) in MCF7/RAGE respect to MCF7/wt cells from RNA-seq.

**Additional file 2:** Down-regulated genes (log2FC ≤ -0.5, *p* < 0.05) in MCF7/RAGE respect to MCF7/wt cells from RNA-seq.

**Additional file 3:** RAGE silencing impairs the migratory and invasivebehavior of RAGE-overexpressing BC cells. Transwell migration (A-B) andinvasion (C-D) assays in wild type and RAGE-overexpressing MCF7 and T47D cells.Cells were counted in at least 5 random fields in three independent experimentsperformed in triplicate, as quantified in the side panels. Scale bar 200 μm. Efficacy of RAGEsilencing in MCF7/RAGE (E) and T47D/RAGE (F) cells. Side panels showdensitometric analysis of the blots normalized to β-actin, which was used as aloading control. Results shown are representative of at least three independentexperiments. (*) indicates *p* < 0.05.

**Additonal file 4:** Analysis of the expression of Ephrin B2 in wild type and RAGE-overexpressing MCF7 cells. mRNA expression of Ephrin B2 in RAGE-overexpressing (MCF7/RAGE) respect to wild type (MCF7/wt) cells, as ascertained by real-time PCR. Values are normalized to the actin beta (ACTB) expression and shown as fold changes of mRNA expression in RAGE-overexpressing respect to wild type cells. Values represent the mean ± SD of three independent experiments performed in triplicate. (*) indicates *p* < 0.05.

**Additional file 5:** The EphA3 inhibitor AWL-II-38.3 does not modify the whole protein levels of EphA3. Immunoblots of EphA3 in MCF7/RAGE (A) and T47D/RAGE (B) cells in the presence or absence of the EphA3 inhibitor AWL-II-38.3. Side panels show densitometric analysis of the blots normalized to β-actin, which was used as a loading control. Values represent the mean ± SD of three independent experiments performed in triplicate.

## Data Availability

All data that were generated or analyzed during our study have been included in this article. Materials, additional data and protocols described within the manuscript will be made available from the authors upon reasonable request.

## Abbreviations

AGEs: Advanced glycation end-products
BC: Breast cancer
CAFs: Cancer-associated fibroblast
DEGs: Differentially expressed genes
ECM: Extracellular matrix
EFNB2: Ephrin B2
EphA3: Ephrin type-A receptor 3
GO: Gene Ontology
KEGG: Kyoto Encyclopedia of Genes and Genomes
MMA: Mithramycin A
RAGE: Receptor for advanced glycation end-products
RNA-seq: RNA sequencing
SEM: Scanning electron microscopy
Sp1: Sp1 transcription factor
TME: Tumor microenvironment
