# Supplementary material for: The Ephrin tyrosine kinase a3 (EphA3) is a novel mediator of RAGE-prompted motility of breast cancer cells
Source: J Exp Clin Cancer Res. 2023 Jul 12;42:164. doi: 10.1186/s13046-023-02747-5 (PMC10337103; doi:10.1186/s13046-023-02747-5)
Supplement: Supplementary file 1 — Additional file 1: Up-regulated genes (log2FC ≥ 0.5, p < 0.05) in MCF7/RAGE respect to MCF7/wt cells from RNA-seq. [file 13046_2023_2747_MOESM1_ESM.pdf]

Additional File 1

Genes	Symbol	Entrez ID	LogFC	P-Value
ENSG00000118946	PCDH17	27253	9.69183022	8.21E-92
ENSG00000184226	PCDH9	5101	8.63637662	4.40E-59
ENSG00000111716	LDHB	3945	8.59160101	0.0158653
ENSG00000203926	SPANXA2	728712	8.11557417	1.33E-35
ENSG00000064763	FAR2	55711	7.4847534	1.45E-30
ENSG00000167165	UGT1A6	54578	6.88900293	9.79E-253
ENSG00000268606	MAGEA2	4101	6.65895113	2.85E-17
ENSG00000139144	PIK3C2G	5288	6.50472622	5.69E-17
ENSG00000269235	ZNF350-AS1	101669766	6.35349335	4.61E-13
ENSG00000183305	MAGEA2B	266740	6.17699311	2.35E-10
ENSG00000221867	MAGEA3	4102	6.12001071	9.12E-76
ENSG00000197172	MAGEA6	4105	6.10485163	1.85E-87
ENSG00000204305	AGER	177	6.02610702	5.27E-149
ENSG00000268916	CSAG3	389903	5.93328298	3.75E-09
ENSG00000233098	CCDC144NL-AS1	440416	5.86045158	2.94E-11
ENSG00000079841	RIMS1	22999	5.83846834	9.57E-07
ENSG00000011677	GABRA3	2556	5.69908084	1.22E-14
ENSG00000145147	SLIT2	9353	5.64327993	0.00173903
ENSG00000111404	RERGL	79785	5.61153221	9.57E-07
ENSG00000177707	NECTIN3	25945	5.58150856	9.57E-07
ENSG00000117114	ADGRL2	23266	5.56682166	0.02967712
ENSG00000198963	RORB	6096	5.35190361	3.75E-09
ENSG00000026025	VIM	7431	5.35095089	0.02553225
ENSG00000241635	UGT1A1	54658	5.09444738	4.19E-07
ENSG00000256612	CYP2B7P	1556	5.08034971	1.96E-30
ENSG00000268902	CSAG2	102723547	4.99156065	0.00048881
ENSG00000156269	NAA11	84779	4.9819881	1.56E-06
ENSG00000157873	TNFRSF14	8764	4.95583122	0.00024445
ENSG00000185737	NRG3	10718	4.90463602	4.11E-09
ENSG00000143891	GALM	130589	4.70644466	1.09E-09
ENSG00000198930	CSAG1	158511	4.70220234	5.22E-27
ENSG00000153993	SEMA3D	223117	4.66929473	2.10E-05
ENSG00000138347	MYPN	84665	4.59492644	5.72E-105
ENSG00000169855	ROBO1	6091	4.57986524	3.57E-06
ENSG00000133401	PDZD2	23037	4.57452869	5.81E-08
ENSG00000009694	TENM1	10178	4.5453241	2.10E-05
ENSG00000240747	KRBOX1	100506243	4.47634389	0.00781606
ENSG00000198021	SPANXA1	30014	4.46977213	0.00781606
ENSG00000121413	ZSCAN18	65982	4.42924963	7.65E-05

ENSG00000196139	AKR1C3	8644	4.39916895	0.00048881
ENSG00000175745	NR2F1	7025	4.33845964	0.00952564
ENSG00000182613	OR2V2	285659	4.32843337	0.01563034
ENSG00000213401	MAGEA12	4111	4.3052992	3.09E-41
ENSG00000152439	ZNF773	374928	4.27911962	0.00024445
ENSG00000268940	CT45A1	541466	4.26551738	2.10E-05
ENSG00000196867	ZFP28	140612	4.21493716	0.00781606
ENSG00000244474	UGT1A4	54657	4.19733654	0.01563034
ENSG00000100078	PLA2G3	50487	4.19111989	1.12E-07
ENSG00000198947	DMD	1756	4.14217088	0.03125763
ENSG00000170017	ALCAM	214	4.10953394	4.61E-39
ENSG00000113209	PCDHB5	26167	4.06746128	1.12E-07
ENSG00000242515	UGT1A10	54575	4.04344685	0.01563034
ENSG00000253953	PCDHGB4	8641	3.99424014	0.00027513
ENSG00000101825	MXRA5	25878	3.97795927	0.01563034
ENSG00000111799	COL12A1	1303	3.88698129	1.63E-06
ENSG00000150281	CTF1	1489	3.78713109	0.00390854
ENSG00000268104	SLC6A14	11254	3.74142067	5.67E-06
ENSG00000165379	LRFN5	145581	3.72831254	1.33E-10
ENSG00000187098	MITF	4286	3.69631065	7.02E-35
ENSG00000250120	PCDHA10	56139	3.63469733	3.13E-12
ENSG00000099260	PALMD	54873	3.54045291	4.02E-05
ENSG00000232677	LINC00665	100506930	3.52557099	0.00097782
ENSG00000184574	LPAR5	57121	3.52439847	0.01172575
ENSG00000167653	PSCA	8000	3.41872657	1.94E-11
ENSG00000271449	CT45A2	728911	3.39294691	0.01563034
ENSG00000240225	ZNF542P	147947	3.38803182	0.01563034
ENSG00000128815	WDFY4	57705	3.34898819	0.03907623
ENSG00000197619	ZNF615	284370	3.33106814	4.59E-13
ENSG00000122025	FLT3	2322	3.297446	1.95E-05
ENSG00000270946	CT45A9	102723680	3.26687218	0.03125763
ENSG00000073849	ST6GAL1	6480	3.26639497	0.00040325
ENSG00000147231	RADX	55086	3.25580211	0.02516849
ENSG00000151632	AKR1C2	1646	3.23969406	2.57E-06
ENSG00000253877	LINC01608	101927459	3.23092015	0.01563034
ENSG00000215182	MUC5AC	4586	3.22652296	0.01563034
ENSG00000178882	RFLNA	100533183	3.212005	5.16E-09
ENSG00000211452	DIO1	1733	3.18348372	0.00072987
ENSG00000145349	CAMK2D	817	3.1737153	0.00117768
ENSG00000136235	GPNMB	10457	3.15939554	2.44E-11
ENSG00000100292	HMOX1	3162	3.14800156	0.02966309
ENSG00000214652	ZNF727	442319	3.14595564	1.38E-08

ENSG00000109610	SOD3	6649	3.11497301	0.03907623
ENSG00000138185	ENTPD1	953	3.08973752	1.00E-13
ENSG00000161551	ZNF577	84765	3.07186247	0.00131423
ENSG00000130513	GDF15	9518	3.03722959	4.91E-33
ENSG00000144366	GULP1	51454	3.02877352	3.11E-34
ENSG00000197951	ZNF71	58491	3.01064545	0.00183304
ENSG00000184012	TMPRSS2	7113	2.92574849	3.32E-12
ENSG00000176046	NUPR1	26471	2.87907954	1.71E-32
ENSG00000044524	EPHA3	2042	2.87477309	0.03125763
ENSG00000134986	NREP	9315	2.7814851	0.01172575
ENSG00000006747	SCIN	85477	2.77618746	5.57E-123
ENSG00000138356	AOX1	316	2.70969118	1.76E-16
ENSG00000083844	ZNF264	9422	2.69538037	4.28E-06
ENSG00000079102	RUNX1T1	862	2.68943484	0.00635238
ENSG00000203326	ZNF525	170958	2.68770615	6.94E-10
ENSG00000185269	NOTUM	147111	2.67919319	5.46E-07
ENSG00000085741	WNT11	7481	2.63440195	3.13E-07
ENSG00000255408	PCDHA3	56145	2.58230184	0.00031163
ENSG00000168843	FSTL5	56884	2.57849644	0.02149439
ENSG00000139112	GABARAPL1	23710	2.5592857	0.00053488
ENSG00000171246	NPTX1	4884	2.4702665	5.02E-13
ENSG00000175592	FOSL1	8061	2.43445819	0.00091258
ENSG00000197360	ZNF98	148198	2.41027009	0.00258074
ENSG00000106853	PTGR1	22949	2.40730643	1.30E-08
ENSG00000157613	CREB3L1	90993	2.40309821	1.08E-06
ENSG00000109625	CPZ	8532	2.39761113	0.0041855
ENSG00000162496	DHRS3	9249	2.38441804	0.00011351
ENSG00000181634	TNFSF15	9966	2.34812422	7.84E-26
ENSG00000171428	NAT1	9	2.34522959	2.25E-24
ENSG00000023839	ABCC2	1244	2.33608652	0.00149224
ENSG00000021645	NRXN3	9369	2.31859742	0.02247458
ENSG00000188959	C9orf152	401546	2.30709881	5.53E-30
ENSG00000108984	MAP2K6	5608	2.30615093	0.00443083
ENSG00000176024	ZNF613	79898	2.15975201	2.82E-07
ENSG00000127084	FGD3	89846	2.13221225	2.56E-22
ENSG00000163281	GNPDA2	132789	2.11374641	0.00435016
ENSG00000256683	ZNF350	59348	2.10384432	4.20E-14
ENSG00000082482	KCNK2	3776	2.08765426	7.06E-05
ENSG00000112183	RBM24	221662	2.07387676	0.00019588
ENSG00000169908	TM4SF1	4071	2.07287729	0.00720686
ENSG00000122863	CHST3	9469	2.06431291	0.00151717
ENSG00000119714	GPR68	8111	2.04453289	0.01273765

ENSG00000136167	LCP1	3936	1.99783482	2.32E-14
ENSG00000198453	ZNF568	374900	1.98583071	0.02247458
ENSG00000170190	SLC16A5	9121	1.95799192	1.90E-06
ENSG00000182168	UNC5C	8633	1.95131491	0.03517795
ENSG00000142556	ZNF614	80110	1.94143718	7.41E-12
ENSG00000136574	GATA4	2626	1.93040766	0.00408454
ENSG00000100036	SLC35E4	339665	1.92596673	0.00047381
ENSG00000111885	MAN1A1	4121	1.92471136	1.04E-31
ENSG00000178685	PARP10	84875	1.91579351	0.00082401
ENSG00000165194	PCDH19	57526	1.91155419	6.85E-15
ENSG00000102683	SGCG	6445	1.89496897	1.46E-19
ENSG00000171631	P2RY6	5031	1.88945016	6.44E-15
ENSG00000148926	ADM	133	1.87899873	0.01183117
ENSG00000167600	CYP2S1	29785	1.85888899	0.01922887
ENSG00000188868	ZNF563	147837	1.84817396	0.03090773
ENSG00000132481	TRIM47	91107	1.84036736	1.93E-05
ENSG00000180818	HOXC10	3226	1.83598672	4.50E-16
ENSG00000164120	HPGD	3248	1.79904653	0.01922887
ENSG00000156011	PSD3	23362	1.79734586	4.60E-20
ENSG00000140470	ADAMTS17	170691	1.77800044	0.00593517
ENSG00000167562	ZNF701	55762	1.75609836	0.00119706
ENSG00000163993	S100P	6286	1.74057181	0.00936983
ENSG00000198521	ZNF43	7594	1.73854835	0.00936983
ENSG00000164128	NPY1R	4886	1.73211243	8.71E-19
ENSG00000147251	DOCK11	139818	1.70585255	0.03877031
ENSG00000086205	FOLH1	2346	1.70396159	8.23E-05
ENSG00000134755	DSC2	1824	1.65869088	8.24E-06
ENSG00000163083	INHBB	3625	1.64937718	2.83E-58
ENSG00000143341	HMCN1	83872	1.63972324	7.09E-127
ENSG00000228836	CT45A5	441521	1.62263066	0.01465289
ENSG00000256087	ZNF432	9668	1.58390692	9.90E-06
ENSG00000111319	SCNN1A	6337	1.56407433	1.72E-19
ENSG00000115641	FHL2	2274	1.5457634	0.00164793
ENSG00000198945	L3MBTL3	84456	1.52711346	0.0226817
ENSG00000166415	WDR72	256764	1.52703776	0.01256056
ENSG00000196267	ZNF836	162962	1.49515441	0.0226817
ENSG00000154654	NCAM2	4685	1.49445243	2.52E-45
ENSG00000183098	GPC6	10082	1.48980311	1.57E-09
ENSG00000267280	TBX2-AS1	103689912	1.48298056	2.02E-09
ENSG00000054983	GALC	2581	1.48139413	1.53E-37
ENSG00000100422	CERK	64781	1.48017004	0.00067188
ENSG00000110811	P3H3	10536	1.47730578	2.56E-05

ENSG00000179023	KLHDC7A	127707	1.47532567	0.04142196
ENSG00000100300	TSPO	706	1.4722494	6.10E-17
ENSG00000198948	MFAP3L	101928198	1.45106473	3.27E-05
ENSG00000092068	SLC7A8	23428	1.44345315	0.00433664
ENSG00000125257	ABCC4	10257	1.44027244	0.00046273
ENSG00000152078	TLCD4	148534	1.43459384	0.0008064
ENSG00000135116	HRK	8739	1.43410966	0.02899262
ENSG00000151726	ACSL1	2180	1.42427502	5.76E-27
ENSG00000157303	SUSD3	203328	1.41731469	1.89E-10
ENSG00000205730	ITPRIPL2	162073	1.41200487	6.71E-22
ENSG00000114529	C3orf52	79669	1.40949263	0.02415234
ENSG00000162817	C1orf115	79762	1.40825069	3.54E-09
ENSG00000196872	CRACDL	343990	1.40256909	1.20E-05
ENSG00000092969	TGFB2	7042	1.40105451	6.04E-05
ENSG00000068366	ACSL4	2182	1.38645365	0.00516516
ENSG00000162891	IL20	50604	1.38383666	0.01135448
ENSG00000136153	LMO7	4008	1.3823105	5.61E-15
ENSG00000171016	PYGO1	26108	1.3613675	0.02948972
ENSG00000204128	C2orf72	257407	1.34130561	0.02415234
ENSG00000170899	GSTA4	2941	1.34092062	0.04332723
ENSG00000100100	PIK3IP1	113791	1.32575616	0.00018485
ENSG00000213694	S1PR3	286223	1.32191372	1.48E-13
ENSG00000165521	EML5	161436	1.31325191	7.75E-05
ENSG00000162670	BRINP3	339479	1.30881789	2.42E-06
ENSG00000109475	RPL34	6164	1.29399247	4.76E-98
ENSG00000040608	RTN4R	65078	1.29074052	0.01388184
ENSG00000155380	SLC16A1	6566	1.27234758	0.00308679
ENSG00000153714	LURAP1L	286343	1.26439449	5.85E-06
ENSG00000151229	SLC2A13	114134	1.24617706	0.02948972
ENSG00000131238	PPT1	5538	1.244456	4.23E-62
ENSG00000042317	SPATA7	55812	1.2413517	1.13E-08
ENSG00000021826	CPS1	1373	1.24012587	1.86E-27
ENSG00000121101	TEX14	56155	1.23034635	0.00553323
ENSG00000177606	JUN	3725	1.23033237	0.00238516
ENSG00000135547	HEY2	23493	1.22539898	0.01662375
ENSG00000111052	LIN7A	8825	1.2209062	0.00026898
ENSG00000154330	PGM5	5239	1.21872365	1.57E-14
ENSG00000168615	ADAM9	8754	1.21772728	1.02E-22
ENSG00000126821	SGPP1	81537	1.21610529	3.62E-14
ENSG00000134668	SPOCD1	90853	1.20805357	0.02886298
ENSG00000184305	CCSER1	401145	1.20097762	0.00022594
ENSG00000185591	SP1	6667	1.19796144	0.00876789

ENSG00000107731	UNC5B	219699	1.19781336	0.01107088
ENSG00000134363	FST	10468	1.19485065	0.02011072
ENSG00000198189	HSD17B11	51170	1.18678216	0.02374648
ENSG00000174939	ASPHD1	253982	1.1863425	3.02E-06
ENSG00000184564	SLITRK6	84189	1.17752688	1.23E-10
ENSG00000148634	HERC4	26091	1.1760865	9.02E-24
ENSG00000188785	ZNF548	147694	1.17278222	0.00647926
ENSG00000065989	PDE4A	5141	1.17143082	0.02886298
ENSG00000219438	TAFA5	25817	1.1697926	0.00077025
ENSG00000052126	PLEKHA5	54477	1.16890832	1.34E-08
ENSG00000109762	SNX25	83891	1.16215361	0.00018851
ENSG00000138162	TACC2	10579	1.16065935	6.88E-19
ENSG00000136826	KLF4	9314	1.14853688	0.00015516
ENSG00000121966	CXCR4	7852	1.14750867	5.49E-05
ENSG00000222041	CYTOR	112597	1.14730093	0.00011105
ENSG00000174718	RESF1	55196	1.14483603	1.75E-09
ENSG00000197050	ZNF420	147923	1.13992857	0.04282405
ENSG00000041982	TNC	3371	1.13867267	0.04101652
ENSG00000144724	PTPRG	5793	1.13314949	7.66E-09
ENSG00000197608	ZNF841	284371	1.13211676	0.00106056
ENSG00000151136	BTBD11	121551	1.12426958	0.01956157
ENSG00000127124	HIVEP3	59269	1.12366172	5.74E-08
ENSG00000125266	EFNB2	1948	1.11724278	0.0026399
ENSG00000269586	CT45A10	102723631	1.1162083	0.00295923
ENSG00000161016	RPL8	6132	1.11467318	1.28E-74
ENSG00000259129	LINC00648	100506433	1.10578435	0.03853984
ENSG00000174915	PTDSS2	81490	1.10236274	4.07E-07
ENSG00000167232	ZNF91	7644	1.09506852	1.79E-05
ENSG00000128274	A4GALT	53947	1.09231157	0.00049513
ENSG00000180730	SHISA2	387914	1.09009428	0.00024046
ENSG00000185133	INPP5J	27124	1.08940755	0.00147627
ENSG00000136048	DRAM1	55332	1.08465412	3.90E-27
ENSG00000196917	HCAR1	27198	1.08392943	5.79E-11
ENSG00000165272	AQP3	360	1.08031182	1.29E-11
ENSG00000198431	TXNRD1	7296	1.0787413	8.87E-44
ENSG00000178896	EXOSC4	54512	1.0770474	4.26E-08
ENSG00000177600	RPLP2	6181	1.06948426	2.93E-75
ENSG00000177694	NAALADL2	254827	1.06929387	0.00032974
ENSG00000170631	ZNF16	7564	1.06521895	0.00019194
ENSG00000166165	CKB	1152	1.05851868	0.04459989
ENSG00000039560	RAI14	26064	1.05710542	0.00011725
ENSG00000166446	CDYL2	124359	1.04779744	3.81E-38

ENSG00000101974	ATP11C	286410	1.04485905	1.05E-12
ENSG00000196549	MME	4311	1.04133843	3.35E-11
ENSG00000188211	NCR3LG1	374383	1.04037878	0.00152863
ENSG00000166289	PLEKHF1	79156	1.03615604	1.26E-05
ENSG00000169783	LINGO1	84894	1.03286014	0.02595114
ENSG00000265688	MAFG-DT	92659	1.03139855	0.00988958
ENSG00000168769	TET2	54790	1.02247024	1.88E-12
ENSG00000142627	EPHA2	1969	1.01419863	0.00435929
ENSG00000204604	ZNF468	90333	1.01393366	3.45E-05
ENSG00000167700	MFSD3	113655	1.00981662	2.43E-06
ENSG00000152229	PSTPIP2	9050	1.00636884	0.00127212
ENSG00000008394	MGST1	4257	1.00511071	4.88E-25
ENSG00000147526	TACC1	6867	1.00277109	7.10E-29
ENSG00000109814	UGDH	7358	1.00247891	6.02E-37
ENSG00000104687	GSR	2936	1.00214953	1.92E-30
ENSG00000182197	EXT1	2131	0.99951375	1.42E-05
ENSG00000182704	TSKU	25987	0.99575568	6.91E-18
ENSG00000198856	OSTC	58505	0.99090977	7.69E-22
ENSG00000122026	RPL21	6144	0.98909738	5.02E-84
ENSG00000236552	RPL13AP5	728658	0.98660658	0.00361818
ENSG00000185885	IFITM1	8519	0.98518203	0.03576482
ENSG00000204611	ZNF616	90317	0.98517042	1.91E-05
ENSG00000104695	PPP2CB	5516	0.98254696	1.74E-08
ENSG00000181218	H2AW	92815	0.98007001	1.05E-07
ENSG00000187231	SESTD1	91404	0.97762782	8.35E-06
ENSG00000166441	RPL27A	6157	0.9756526	2.78E-84
ENSG00000120875	DUSP4	1846	0.97508654	1.33E-26
ENSG00000170293	CMTM8	152189	0.97083794	0.02936755
ENSG00000241360	PDXP	57026	0.97044196	0.01351926
ENSG00000064666	CNN2	1265	0.96896184	7.06E-15
ENSG00000103126	AXIN1	8312	0.96657415	5.22E-18
ENSG00000226950	DANCR	57291	0.96646458	2.83E-18
ENSG00000179632	MAF1	84232	0.96191337	5.39E-30
ENSG00000078401	EDN1	1906	0.96117716	0.00116279
ENSG00000167315	ACAA2	10449	0.96107701	8.49E-30
ENSG00000112208	BAG2	9532	0.96032479	0.00180523
ENSG00000122042	UBL3	5412	0.95025557	1.39E-32
ENSG00000170619	COMMD5	28991	0.94228214	1.34E-07
ENSG00000130193	THEM6	51337	0.93884342	3.26E-21
ENSG00000237515	SHISA9	729993	0.93780083	0.00021301
ENSG00000070778	PTPN21	11099	0.93696314	2.86E-07
ENSG00000167705	RILP	83547	0.93688635	0.02707373

ENSG00000120896	SORBS3	10174	0.93299658	4.12E-08
ENSG00000183579	ZNRF3	84133	0.92970497	0.00607217
ENSG00000104529	EEF1D	1936	0.92924392	2.36E-41
ENSG00000234616	JRK	8629	0.9284507	4.12E-08
ENSG00000163931	TKT	7086	0.92801009	6.06E-55
ENSG00000079459	FDFT1	2222	0.92535445	1.42E-33
ENSG00000132329	RAMP1	10267	0.92420146	0.02481345
ENSG00000150403	TMCO3	55002	0.92365536	1.62E-08
ENSG00000198682	PAPSS2	9060	0.91857475	0.00119117
ENSG00000147677	EIF3H	8667	0.91831274	5.11E-38
ENSG00000218336	TENM3	55714	0.91713652	0.00019773
ENSG00000184349	EFNA5	1946	0.91638346	0.01282657
ENSG00000185803	SLC52A2	79581	0.91258696	3.62E-11
ENSG00000261236	BOP1	23246	0.90988337	1.37E-10
ENSG00000235173	HGH1	51236	0.90948094	5.63E-07
ENSG00000133112	TPT1	7178	0.90836084	3.98E-39
ENSG00000053254	FOXN3	1112	0.90582572	2.08E-13
ENSG00000186603	HPDL	84842	0.90550454	0.00425564
ENSG00000104419	NDRG1	10397	0.90336053	9.51E-08
ENSG00000102302	FGD1	2245	0.89744751	2.48E-05
ENSG00000129993	CBFA2T3	863	0.89703145	8.46E-16
ENSG00000130821	SLC6A8	6535	0.89329036	4.62E-19
ENSG00000013364	MVP	9961	0.88924133	0.00425564
ENSG00000169439	SDC2	6383	0.88094242	0.0035442
ENSG00000139146	SINHCAF	58516	0.88085265	3.58E-11
ENSG00000162599	NFIA	4774	0.88039978	1.38E-05
ENSG00000147799	ARHGAP39	80728	0.87848751	3.22E-06
ENSG00000144218	AFF3	3899	0.875549	0.00020514
ENSG00000242125	SNHG3	8420	0.87470634	1.92E-06
ENSG00000113532	ST8SIA4	7903	0.87425495	2.33E-05
ENSG00000231131	LNCAROD	105378305	0.87085873	0.00018862
ENSG00000137393	RNF144B	255488	0.8707632	0.00020647
ENSG00000128283	CDC42EP1	11135	0.86719113	5.11E-05
ENSG00000071051	NCK2	8440	0.86058723	1.08E-05
ENSG00000081870	HSPB11	51668	0.85472915	0.00560977
ENSG00000027075	PRKCH	5583	0.85393722	4.64E-15
ENSG00000269858	EGLN2	112398	0.84930309	1.27E-06
ENSG00000126778	SIX1	6495	0.84911468	0.01068915
ENSG00000117399	CDC20	991	0.84573134	0.02011283
ENSG00000161638	ITGA5	3678	0.84548137	0.00883108
ENSG00000138814	PPP3CA	5530	0.84370333	5.55E-15
ENSG00000182541	LIMK2	3985	0.84215687	7.88E-05

ENSG00000256771	ZNF253	56242	0.84191222	0.00960245
ENSG00000184428	TOP1MT	116447	0.84185497	1.56E-08
ENSG00000175390	EIF3F	8665	0.83926464	1.45E-20
ENSG00000147224	PRPS1	5631	0.83816286	0.00088892
ENSG00000164237	CMBL	134147	0.8343478	3.26E-20
ENSG00000162909	CAPN2	824	0.83413701	0.01121641
ENSG00000087301	TXNDC16	57544	0.83072453	3.84E-06
ENSG00000174032	SLC25A30	253512	0.82956619	9.37E-05
ENSG00000185122	HSF1	3297	0.8257913	2.33E-17
ENSG00000116016	EPAS1	2034	0.81603623	2.27E-06
ENSG00000198964	SGMS1	259230	0.81509218	2.08E-06
ENSG00000172965	MIR4435-2HG	541471	0.81349191	0.0002647
ENSG00000147676	MAL2	114569	0.8134714	1.68E-18
ENSG00000174720	LARP7	51574	0.81246983	1.76E-06
ENSG00000197128	ZNF772	400720	0.811031	0.0086869
ENSG00000181026	AEN	64782	0.81089735	1.95E-05
ENSG00000073792	IGF2BP2	10644	0.80959265	0.02454408
ENSG00000117859	OSBPL9	114883	0.80811671	2.57E-13
ENSG00000186184	POLR1D	51082	0.80799205	1.11E-20
ENSG00000176371	ZSCAN2	54993	0.80535348	0.01790792
ENSG00000177030	DEAF1	10522	0.80383681	0.00011936
ENSG00000127585	FBXL16	146330	0.80382943	4.56E-08
ENSG00000100129	EIF3L	51386	0.80331614	6.28E-39
ENSG00000184545	DUSP8	1850	0.80269747	0.02454408
ENSG00000008853	RHOBTB2	23221	0.80257076	4.77E-07
ENSG00000133789	SWAP70	23075	0.80182723	0.00128586
ENSG00000189376	C8orf76	84933	0.79883114	7.40E-07
ENSG00000129925	PGAP6	58986	0.79763306	8.65E-10
ENSG00000152270	PDE3B	5140	0.79680766	0.00201165
ENSG00000119900	OGFRL1	79627	0.79611671	2.41E-07
ENSG00000147421	HMBOX1	79618	0.79483197	0.02958417
ENSG00000142541	RPL13A	23521	0.79462226	1.77E-30
ENSG00000132254	ARFIP2	23647	0.79348476	5.20E-13
ENSG00000237330	RNF223	401934	0.7922883	0.00196622
ENSG00000169972	PUSL1	126789	0.78966641	0.00847301
ENSG00000120889	TNFRSF10B	8795	0.78926044	6.07E-07
ENSG00000163435	ELF3	1999	0.78836056	1.53E-06
ENSG00000154274	C4orf19	55286	0.78568666	0.00258676
ENSG00000137154	RPS6	6194	0.78326577	3.79E-64
ENSG00000081923	ATP8B1	5205	0.78275822	1.41E-08
ENSG00000133121	STARD13	90627	0.78226112	0.00023705
ENSG00000180198	RCC1	1104	0.78118821	7.34E-11

ENSG00000137203	TFAP2A	7020	0.77794387	0.00116676
ENSG00000181588	MEX3D	399664	0.77708521	0.00911836
ENSG00000236699	ARHGEF38	54848	0.77702063	0.01609895
ENSG00000171169	NAIF1	203245	0.77544373	0.00536258
ENSG00000123388	HOXC11	3227	0.774785	0.03202564
ENSG00000171295	ZNF440	126070	0.77442198	0.02872458
ENSG00000171443	ZNF524	147807	0.77051063	0.00024966
ENSG00000136111	TBC1D4	9882	0.76755534	0.00024966
ENSG00000105640	RPL18A	6142	0.76572422	1.50E-29
ENSG00000183309	ZNF623	9831	0.76403353	8.72E-07
ENSG00000170545	SMAGP	57228	0.76401385	0.00064459
ENSG00000167106	FAM102A	399665	0.76363768	3.59E-15
ENSG00000116574	RHOU	58480	0.760161	0.01088506
ENSG00000116251	RPL22	6146	0.75942939	8.71E-38
ENSG00000131831	RAI2	10742	0.75857258	0.00289058
ENSG00000164713	BRI3	25798	0.75682774	0.00062449
ENSG00000114805	PLCH1	23007	0.75628899	0.01123873
ENSG00000181061	HIGD1A	25994	0.75579928	9.90E-05
ENSG00000015133	CCDC88C	440193	0.75369906	1.30E-06
ENSG00000214026	MRPL23	107987373	0.75272704	0.00016855
ENSG00000065978	YBX1	4904	0.75087617	9.00E-10
ENSG00000138802	SEC24B	10427	0.75007771	7.52E-07
ENSG00000006062	MAP3K14	9020	0.74899327	0.00664488
ENSG00000104689	TNFRSF10A	8797	0.74828747	0.03706963
ENSG00000197020	ZNF100	163227	0.74784099	0.03089212
ENSG00000145332	KLHL8	57563	0.74534717	0.00017467
ENSG00000140988	RPS2	6187	0.74310016	1.61E-41
ENSG00000105327	BBC3	27113	0.74240888	0.00320671
ENSG00000157985	AGAP1	116987	0.74150712	1.66E-07
ENSG00000169122	FAM110B	90362	0.73977632	0.02956812
ENSG00000178719	GRINA	2907	0.73849708	2.82E-26
ENSG00000135090	TAOK3	51347	0.73833547	6.33E-08
ENSG00000171612	SLC25A33	84275	0.73648678	0.03706963
ENSG00000064651	SLC12A2	6558	0.73342183	9.12E-13
ENSG00000012660	ELOVL5	60481	0.73245892	8.74E-12
ENSG00000085840	ORC1	4998	0.72860727	0.02956812
ENSG00000013583	HEBP1	50865	0.72816029	8.17E-08
ENSG00000112852	PCDHB2	56133	0.72639332	0.02641118
ENSG00000182208	MOB2	81532	0.72479624	0.01729429
ENSG00000119729	RHOQ	23433	0.72435409	9.72E-13
ENSG00000104763	ASAH1	427	0.72254324	9.97E-10
ENSG00000129422	MTUS1	57509	0.72084833	8.96E-05

ENSG00000104290	FZD3	7976	0.72065611	0.0002945
ENSG00000100290	BIK	638	0.71979733	0.02956812
ENSG00000182287	AP1S2	8905	0.71862578	0.00106135
ENSG00000198355	PIM3	415116	0.71845875	1.27E-07
ENSG00000170889	RPS9	6203	0.71805398	2.83E-39
ENSG00000107262	BAG1	573	0.71163879	9.74E-15
ENSG00000177570	SAMD12	401474	0.7094642	0.00154555
ENSG00000083635	NUFIP1	26747	0.70753789	0.0247251
ENSG00000114315	HES1	3280	0.70552654	0.00069929
ENSG00000077782	FGFR1	2260	0.70431517	9.06E-07
ENSG00000146376	ARHGAP18	93663	0.70368616	0.00033246
ENSG00000142230	SAE1	10055	0.70296306	1.08E-12
ENSG00000090661	CERS4	79603	0.70156697	0.00016586
ENSG00000012232	EXTL3	2137	0.70109049	7.63E-05
ENSG00000085276	MECOM	2122	0.70087722	0.03966267
ENSG00000099800	TIMM13	26517	0.70085513	0.00139193
ENSG00000187954	CYHR1	50626	0.69927342	8.97E-08
ENSG00000088930	XRN2	22803	0.69793997	4.82E-17
ENSG00000103855	CD276	80381	0.6959183	2.87E-10
ENSG00000120685	PROSER1	80209	0.69432099	3.05E-07
ENSG00000112245	PTP4A1	7803	0.69399095	3.22E-11
ENSG00000233927	RPS28	6234	0.68934069	1.16E-28
ENSG00000136159	NUDT15	55270	0.68869348	0.00858132
ENSG00000115758	ODC1	4953	0.68772217	3.00E-07
ENSG00000087074	PPP1R15A	23645	0.68700714	0.01016794
ENSG00000124766	SOX4	6659	0.6865172	4.30E-08
ENSG00000133657	ATP13A3	79572	0.68361204	5.64E-12
ENSG00000189337	KAZN	23254	0.68258964	0.04752587
ENSG00000122406	RPL5	6125	0.68210394	3.88E-42
ENSG00000150893	FREM2	341640	0.68054434	1.67E-08
ENSG00000111912	NCOA7	135112	0.67972152	0.01426604
ENSG00000122705	CLTA	1211	0.67922196	6.56E-08
ENSG00000060138	YBX3	8531	0.67750842	1.01E-08
ENSG00000167658	EEF2	1938	0.67717369	1.27E-34
ENSG00000022267	FHL1	2273	0.6771361	0.00240521
ENSG00000070047	PHRF1	57661	0.67550861	1.40E-05
ENSG00000170522	ELOVL6	79071	0.67363833	4.60E-09
ENSG00000014164	ZC3H3	23144	0.67298519	0.00033935
ENSG00000116685	KIAA2013	90231	0.67294733	2.13E-05
ENSG00000138660	AP1AR	55435	0.67203211	3.70E-06
ENSG00000133119	RFC3	5983	0.67005602	0.00079302
ENSG00000145808	ADAMTS19	171019	0.66878351	9.17E-05

ENSG00000104221	BRF2	55290	0.66851996	0.03354418
ENSG00000178568	ERBB4	2066	0.66837239	9.59E-05
ENSG00000131269	ABCB7	22	0.6650704	0.00358389
ENSG00000005801	ZNF195	7748	0.6648324	0.01784112
ENSG00000104723	TUSC3	7991	0.66354966	0.00143119
ENSG00000064932	SBNO2	22904	0.66341694	2.72E-06
ENSG00000171208	NETO2	81831	0.66223501	2.91E-05
ENSG00000148700	ADD3	120	0.66180015	0.03168044
ENSG00000123200	ZC3H13	23091	0.66018049	6.33E-09
ENSG00000164023	SGMS2	166929	0.65985159	0.04767428
ENSG00000136986	DERL1	79139	0.65967061	7.32E-14
ENSG00000069011	PITX1	5307	0.65951536	0.03765806
ENSG00000147789	ZNF7	7553	0.65912708	0.00304548
ENSG00000168077	SCARA3	51435	0.65884508	0.01689181
ENSG00000103260	METRN	79006	0.6574022	7.45E-08
ENSG00000121068	TBX2	6909	0.65600087	2.61E-10
ENSG00000179091	CYC1	1537	0.65379939	5.53E-14
ENSG00000167965	MLST8	64223	0.65347818	2.34E-09
ENSG00000156508	EEF1A1	1915	0.65342113	1.64E-23
ENSG00000187840	EIF4EBP1	1978	0.65331122	0.00027573
ENSG00000049246	PER3	8863	0.65215609	0.00017158
ENSG00000103811	CTSH	1512	0.65160827	0.00770398
ENSG00000224051	CPTP	80772	0.65147635	0.00689432
ENSG00000003436	TFPI	7035	0.64977374	0.01575796
ENSG00000179889	PDXDC1	23042	0.64779474	4.42E-20
ENSG00000162623	TYW3	127253	0.6476118	0.00725763
ENSG00000163938	GNL3	26354	0.64678924	1.26E-05
ENSG00000115365	LANCL1	10314	0.646557	1.00E-08
ENSG00000166483	WEE1	7465	0.64226153	0.00520451
ENSG00000165572	KBTBD6	89890	0.64181999	0.00088283
ENSG00000073417	PDE8A	5151	0.64014102	1.34E-06
ENSG00000111058	ACSS3	79611	0.63968412	0.00318957
ENSG00000054277	OPN3	23596	0.63903269	0.04189013
ENSG00000188342	GTF2F2	2963	0.63890858	0.00335158
ENSG00000170100	ZNF778	197320	0.63797195	0.01076652
ENSG00000171425	ZNF581	51545	0.63786387	3.23E-05
ENSG00000049759	NEDD4L	23327	0.63760328	0.00031982
ENSG00000156671	SAMD8	142891	0.63692534	0.00051777
ENSG00000119138	KLF9	687	0.63671178	0.04189013
ENSG00000196141	SPATS2L	26010	0.63470816	0.00853193
ENSG00000141376	BCAS3	54828	0.63449703	3.54E-15
ENSG00000170502	NUDT9	53343	0.63441219	0.00087755

ENSG00000204899	MZT1	440145	0.6333727	0.0135414
ENSG00000107404	DVL1	1855	0.63309272	7.58E-06
ENSG00000132128	LRRC41	10489	0.63242923	6.19E-07
ENSG00000100316	RPL3	6122	0.63224503	8.77E-39
ENSG00000120697	ALG5	29880	0.63111027	0.00494824
ENSG00000167747	C19orf48	84798	0.63058179	2.03E-10
ENSG00000165156	ZHX1	11244	0.63025816	1.07E-10
ENSG00000123908	ago-02	27161	0.62960507	3.85E-08
ENSG00000155850	SLC26A2	1836	0.62911295	8.35E-06
ENSG00000147955	SIGMAR1	10280	0.629109	0.00963631
ENSG00000108448	TRIM16L	147166	0.62816207	0.00335158
ENSG00000109321	AREG	374	0.62735681	1.02E-06
ENSG00000100410	PHF5A	84844	0.62698734	0.01177636
ENSG00000089006	SNX5	27131	0.62545215	1.89E-08
ENSG00000121350	PYROXD1	79912	0.62535361	0.03722334
ENSG00000154930	ACSS1	84532	0.62482752	0.00012219
ENSG00000116212	LRRC42	115353	0.62445798	0.00610882
ENSG00000261609	GAN	8139	0.62381252	0.0440448
ENSG00000177700	POLR2L	5441	0.62339805	3.80E-08
ENSG00000132286	TIMM10B	26515	0.62257626	0.0010637
ENSG00000166387	PPFIBP2	8495	0.62139863	0.01194807
ENSG00000096968	JAK2	3717	0.61962519	0.02039813
ENSG00000152520	PAN3	255967	0.61886808	0.00021968
ENSG00000099284	MACROH2A2	55506	0.61863463	0.00156341
ENSG00000142864	SERBP1	26135	0.61842865	1.01E-05
ENSG00000139517	LNX2	222484	0.61783211	8.50E-05
ENSG00000140455	USP3	9960	0.61779976	0.00010367
ENSG00000180900	SCRIB	23513	0.61525266	3.23E-10
ENSG00000135486	HNRNPA1	3178	0.61517388	1.18E-48
ENSG00000119899	SLC17A5	26503	0.61512843	0.01655194
ENSG00000057294	PKP2	5318	0.61490195	0.00020457
ENSG00000232388	SMIM26	388789	0.61420715	5.20E-05
ENSG00000115107	STEAP3	55240	0.61416978	0.00315289
ENSG00000130255	RPL36	25873	0.61406055	3.43E-21
ENSG00000197265	GTF2E2	2961	0.61274112	0.01121983
ENSG00000102103	PQBP1	10084	0.61180547	0.00028123
ENSG00000172009	THOP1	7064	0.61047959	0.00100013
ENSG00000181019	NQO1	1728	0.60915837	7.91E-12
ENSG00000133114	GPALPP1	55425	0.6085906	0.00704326
ENSG00000140743	CDR2	1039	0.60788073	0.02184047
ENSG00000049130	KITLG	4254	0.6078553	2.78E-05
ENSG00000174444	RPL4	6124	0.60719223	1.55E-61

ENSG00000122033	MTIF3	219402	0.60676309	0.00049358
ENSG00000179832	MROH1	727957	0.60646403	0.00027698
ENSG00000197785	ATAD3A	55210	0.60634851	0.00191488
ENSG00000132970	WASF3	10810	0.60538769	0.00528041
ENSG00000175175	PPM1E	22843	0.60452884	0.00022398
ENSG00000159873	CCDC117	150275	0.60377741	9.28E-07
ENSG00000165185	KIAA1958	158405	0.60371044	0.00787491
ENSG00000182774	RPS17	6218	0.60282736	1.19E-32
ENSG00000162783	IER5	51278	0.6019255	0.00396239
ENSG00000119655	NPC2	10577	0.60174982	6.44E-07
ENSG00000163644	PPM1K	152926	0.60172553	0.00921856
ENSG00000166337	TAF10	6881	0.60047886	0.00088228
ENSG00000102763	VWA8	23078	0.59894316	0.01447923
ENSG00000198169	ZNF251	90987	0.59886571	0.0275282
ENSG00000117000	RLF	6018	0.59867855	0.01031468
ENSG00000087076	HSD17B14	51171	0.59601615	0.01261352
ENSG00000153786	ZDHHC7	55625	0.59497304	1.07E-08
ENSG00000173545	ZNF622	90441	0.59473338	0.00047423
ENSG00000102780	DGKH	160851	0.59415844	0.01207381
ENSG00000099219	ERMP1	79956	0.59378818	2.17E-08
ENSG00000058804	NDC1	55706	0.59366051	0.00892766
ENSG00000117620	SLC35A3	23443	0.59239548	0.00188138
ENSG00000165929	TC2N	123036	0.5894002	6.51E-08
ENSG00000145604	SKP2	6502	0.5891387	0.00737038
ENSG00000142937	RPS8	6202	0.58832959	1.01E-41
ENSG00000197858	GPAA1	8733	0.58748303	5.12E-09
ENSG00000181788	SIAH2	6478	0.58676012	3.42E-09
ENSG00000101004	NINL	22981	0.58596225	0.01126753
ENSG00000242173	ARHGDIG	398	0.58574059	0.02811009
ENSG00000169862	CTNND2	1501	0.58503315	6.54E-07
ENSG00000198911	SREBF2	6721	0.58353675	1.05E-12
ENSG00000239900	ADSL	158	0.58346949	9.07E-05
ENSG00000133872	SARAF	51669	0.58131304	6.64E-10
ENSG00000120686	UFM1	51569	0.58045317	5.83E-05
ENSG00000133103	COG6	57511	0.58043003	0.00104815
ENSG00000162437	RAVER2	55225	0.58035748	0.00939213
ENSG00000160007	ARHGAP35	2909	0.5793236	1.72E-15
ENSG00000177951	BET1L	51272	0.57832886	0.00032289
ENSG00000112972	HMGCS1	3157	0.57768408	1.36E-07
ENSG00000115183	TANC1	85461	0.57701733	0.00135589
ENSG00000134508	CABLES1	91768	0.57660011	6.47E-05
ENSG00000120688	WBP4	11193	0.57594478	0.01930052

ENSG00000168743	NPNT	255743	0.57582226	4.05E-12
ENSG00000177156	TALDO1	6888	0.57541473	3.30E-10
ENSG00000125844	RRBP1	6238	0.57530177	3.84E-07
ENSG00000078369	GNB1	2782	0.5735114	1.48E-15
ENSG00000175274	TP53I11	9537	0.57340296	2.05E-05
ENSG00000104880	ARHGEF18	23370	0.57295149	0.00346461
ENSG00000130766	SESN2	83667	0.57285934	0.0232841
ENSG00000125629	INSIG2	51141	0.57261501	0.00601379
ENSG00000142534	RPS11	6205	0.57256994	1.54E-26
ENSG00000183020	AP2A2	161	0.57045722	9.24E-05
ENSG00000130227	XPO7	23039	0.56981817	1.78E-06
ENSG00000177225	GATD1	347862	0.56920652	0.00376294
ENSG00000148175	STOM	2040	0.56912261	4.33E-08
ENSG00000167513	CDT1	81620	0.56889436	0.00025867
ENSG00000117450	PRDX1	5052	0.56867725	2.00E-19
ENSG00000072401	UBE2D1	7321	0.56825615	0.03864362
ENSG00000145592	RPL37	6167	0.56795754	2.29E-34
ENSG00000126067	PSMB2	5690	0.5668318	7.57E-05
ENSG00000085788	DDHD2	23259	0.56636779	0.00177375
ENSG00000147874	HAUS6	54801	0.56628478	0.02195977
ENSG00000104671	DCTN6	10671	0.5657703	0.01095504
ENSG00000102804	TSC22D1	8848	0.56571173	1.79E-07
ENSG00000177697	CD151	977	0.5656646	1.72E-09
ENSG00000175324	LSM1	27257	0.56509863	0.00979225
ENSG00000156735	BAG4	9530	0.56486161	0.01095504
ENSG00000105372	RPS19	6223	0.56467543	2.47E-37
ENSG00000168028	RPSA	3921	0.56408334	4.18E-23
ENSG00000141646	SMAD4	4089	0.56389417	0.00226924
ENSG00000100209	HSCB	150274	0.5636402	0.00566909
ENSG00000120696	KBTBD7	84078	0.56339185	0.00507746
ENSG00000132746	ALDH3B2	222	0.56318177	2.67E-09
ENSG00000110651	CD81	975	0.56242174	5.28E-10
ENSG00000173660	UQCRH	7388	0.5621591	1.24E-06
ENSG00000154380	ENAH	55740	0.56213128	0.00125811
ENSG00000100354	TNRC6B	23112	0.56127804	0.00679905
ENSG00000112306	RPS12	6206	0.56116092	9.44E-29
ENSG00000100243	CYB5R3	1727	0.55984719	1.73E-06
ENSG00000122644	ARL4A	10124	0.55941129	0.00030192
ENSG00000087088	BAX	581	0.55815131	1.88E-05
ENSG00000184014	DENND5A	23258	0.55718011	0.00327438
ENSG00000137309	HMGA1	3159	0.55602551	2.04E-15
ENSG00000179950	PUF60	22827	0.5550355	9.20E-11

ENSG00000131941	RHPN2	85415	0.55417438	2.86E-07
ENSG00000166471	TMEM41B	440026	0.55325824	0.00273764
ENSG00000153561	RMND5A	64795	0.55108458	6.70E-07
ENSG00000139684	ESD	2098	0.551056	1.25E-07
ENSG00000160948	VPS28	51160	0.55093257	4.76E-07
ENSG00000152137	HSPB8	26353	0.55092899	0.00011015
ENSG00000023516	AKAP11	11215	0.55091481	4.92E-05
ENSG00000135480	KRT7	3855	0.55059456	0.04526259
ENSG00000175602	CCDC85B	11007	0.54954884	0.01666087
ENSG00000087086	FTL	2512	0.54932223	1.02E-16
ENSG00000141101	NOB1	28987	0.54908572	3.20E-07
ENSG00000100029	PES1	23481	0.54907213	0.00011747
ENSG00000076864	RAP1GAP	5909	0.54902998	0.00023707
ENSG00000188846	RPL14	9045	0.54887359	5.36E-27
ENSG00000136152	COG3	83548	0.54859416	0.00034849
ENSG00000099246	RAB18	22931	0.54833145	3.34E-07
ENSG00000102870	ZNF629	23361	0.54813041	1.21E-05
ENSG00000177105	RHOG	391	0.54757014	0.01435891
ENSG00000138801	PAPSS1	9061	0.5471373	7.25E-06
ENSG00000050405	LIMA1	51474	0.54620568	1.77E-05
ENSG00000123144	TRIR	79002	0.54460398	5.31E-08
ENSG00000076826	CAMSAP3	57662	0.54370792	0.00021441
ENSG00000113504	SLC12A7	10723	0.54331849	0.0005527
ENSG00000162368	CMPK1	51727	0.54302011	3.12E-06
ENSG00000123143	PKN1	5585	0.54259584	1.68E-09
ENSG00000180921	FAM83H	286077	0.54228054	2.67E-11
ENSG00000109654	TRIM2	23321	0.54054689	0.01866435
ENSG00000206053	JPT2	90861	0.53895342	7.98E-21
ENSG00000130159	ECSIT	51295	0.53847694	0.00063752
ENSG00000143153	ATP1B1	481	0.53808127	9.46E-10
ENSG00000100266	PACSIN2	11252	0.53795156	1.22E-05
ENSG00000073169	SELENOO	83642	0.53781953	0.02015425
ENSG00000198816	ZNF358	140467	0.53777361	0.00409834
ENSG00000143514	TP53BP2	7159	0.53747382	0.01602836
ENSG00000109534	GAR1	54433	0.53713081	0.02258252
ENSG00000184292	TACSTD2	4070	0.53711158	3.24E-06
ENSG00000165233	CARD19	84270	0.53461596	0.01730307
ENSG00000163683	SMIM14	201895	0.53441499	1.20E-11
ENSG00000205213	LGR4	55366	0.53418599	0.00589431
ENSG00000142453	CARM1	10498	0.53322012	2.09E-06
ENSG00000171490	RSL1D1	26156	0.53236899	1.81E-14
ENSG00000165392	WRN	7486	0.53189837	0.0401492

ENSG00000078674	PCM1	5108	0.53177432	9.31E-06
ENSG00000071082	RPL31	6160	0.53131779	7.90E-29
ENSG00000116221	MRPL37	51253	0.53099155	3.30E-05
ENSG00000007047	MARK4	57787	0.53046561	0.00510103
ENSG00000183762	KREMEN1	83999	0.53009337	0.03052006
ENSG00000167173	C15orf39	56905	0.52975433	0.01602836
ENSG00000118960	HS1BP3	64342	0.52724956	0.00059463
ENSG00000127870	RNF6	6049	0.52723822	0.00036334
ENSG00000174574	AKIRIN1	79647	0.52690256	0.0061261
ENSG00000132964	CDK8	1024	0.52674708	0.0281966
ENSG00000130522	JUND	3727	0.52533195	0.00116263
ENSG00000179526	SHARPIN	81858	0.52530749	0.00317983
ENSG00000099804	CDC34	997	0.52506886	0.00354328
ENSG00000176853	FAM91A1	157769	0.52361047	4.97E-09
ENSG00000138326	RPS24	6229	0.52189559	2.97E-30
ENSG00000165637	VDAC2	7417	0.52110932	1.38E-08
ENSG00000111859	NEDD9	4739	0.52058569	0.03840827
ENSG00000164323	CFAP97	57587	0.52040092	0.00084186
ENSG00000065150	IPO5	3843	0.51980765	1.02E-07
ENSG00000104969	SGTA	6449	0.51977041	8.67E-05
ENSG00000100296	THOC5	8563	0.51906873	0.00566245
ENSG00000169756	LIMS1	3987	0.51843806	0.00047844
ENSG00000134419	RPS15A	6210	0.51835084	8.22E-18
ENSG00000120149	MSX2	4488	0.51823641	0.01188071
ENSG00000162402	USP24	23358	0.51799976	0.00246676
ENSG00000186468	RPS23	6228	0.51782957	1.23E-23
ENSG00000089289	IGBP1	3476	0.51771444	5.59E-07
ENSG00000100353	EIF3D	8664	0.51716624	2.74E-14
ENSG00000100380	ST13	6767	0.51686083	1.07E-10
ENSG00000089157	RPLP0	6175	0.51684095	1.39E-13
ENSG00000167657	DAPK3	1613	0.51664192	0.01484569
ENSG00000175756	AURKAIP1	54998	0.51649986	9.56E-05
ENSG00000137818	RPLP1	6176	0.51580791	1.75E-22
ENSG00000105373	NOP53	29997	0.51298691	1.08E-12
ENSG00000184281	TSSC4	10078	0.51284654	0.01316826
ENSG00000132963	POMP	51371	0.51233605	0.00035886
ENSG00000083845	RPS5	6193	0.51228006	1.16E-15
ENSG00000118816	CCNI	10983	0.51156393	9.48E-15
ENSG00000114942	EEF1B2	1933	0.51137435	4.06E-21
ENSG00000261150	EPPK1	83481	0.51059609	0.00067745
ENSG00000159023	EPB41	2035	0.51055529	0.01376434
ENSG00000156795	NTAQ1	55093	0.51050117	0.0164383

ENSG00000126777	KTN1	3895	0.50975177	4.28E-11
ENSG00000124839	RAB17	64284	0.50961269	0.00072253
ENSG00000168268	NT5DC2	64943	0.508904	3.05E-05
ENSG00000115339	GALNT3	2591	0.50882518	8.30E-05
ENSG00000166803	PCLAF	9768	0.50774797	0.0097811
ENSG00000162601	MYSM1	114803	0.50751848	0.01718438
ENSG00000169255	B3GALNT1	8706	0.50581901	6.71E-06
ENSG00000132823	OSER1	51526	0.50539581	0.00945832
ENSG00000103152	MPG	4350	0.50311056	3.75E-06
ENSG00000169682	SPNS1	83985	0.50306836	6.44E-05
ENSG00000173418	NAA20	51126	0.50239844	0.00012808
ENSG00000183779	ZNF703	80139	0.50206567	1.96E-06
ENSG00000131873	CHSY1	22856	0.50188093	0.02121559
ENSG00000185043	CIB1	10519	0.50142265	0.0005015
ENSG00000103415	HMOX2	3163	0.50018633	9.32E-05
