# Supplementary material for: The Ephrin tyrosine kinase a3 (EphA3) is a novel mediator of RAGE-prompted motility of breast cancer cells
Source: J Exp Clin Cancer Res. 2023 Jul 12;42:164. doi: 10.1186/s13046-023-02747-5 (PMC10337103; doi:10.1186/s13046-023-02747-5)
Supplement: Supplementary file 2 — Additional file 2: Down-regulated genes (log2FC ≤ -0.5, p < 0.05) in MCF7/RAGE respect to MCF7/wt cells from RNA-seq. [file 13046_2023_2747_MOESM2_ESM.pdf]

Additional File 2

Genes	Symbol	Entrez ID	LogFC	P-Value
ENSG00000065534	MYLK	4638	-5.7707721	1.38E-50
ENSG00000256870	SLC5A8	160728	-5.1176764	1.47E-10
ENSG00000126947	ARMCX1	51309	-5.0187859	0.00048881
ENSG00000158258	CLSTN2	64084	-4.998478	5.08E-29
ENSG00000170819	BFSP2	8419	-4.9774193	4.11E-09
ENSG00000231528	FAM225A	286333	-4.8709483	0.00195456
ENSG00000100234	TIMP3	7078	-4.7415882	9.94E-41
ENSG00000172137	CALB2	794	-4.6999547	0.00012225
ENSG00000198771	RCSD1	92241	-4.5738484	0.00390854
ENSG00000137843	PAK6	106821730	-4.5628851	0.00012225
ENSG00000170011	MYRIP	25924	-4.5291811	3.06E-05
ENSG00000215039	CD27-AS1	678655	-4.3666576	5.57E-21
ENSG00000171401	KRT13	3860	-4.1531409	3.06E-05
ENSG00000175356	SCUBE2	57758	-4.1163625	2.99E-06
ENSG00000173335	CST9	128822	-4.0604236	0.03125763
ENSG00000137875	BCL2L10	10017	-3.7976573	0.03125763
ENSG00000163873	GRIK3	2899	-3.7444395	4.02E-05
ENSG00000103489	XYLT1	64131	-3.6383919	2.87E-09
ENSG00000135905	DOCK10	55619	-3.5954389	0.00195456
ENSG00000174791	RIN1	9610	-3.576812	1.63E-06
ENSG00000231133	HAR1B	768097	-3.5581145	2.99E-06
ENSG00000109472	CPE	1363	-3.5485384	2.57E-47
ENSG00000181449	SOX2	6657	-3.5402783	1.95E-05
ENSG00000082175	PGR	5241	-3.4947287	4.68E-10
ENSG00000166432	ZMAT1	84460	-3.4686359	1.32E-07
ENSG00000140682	TGFB1I1	7041	-3.4205062	0.00051957
ENSG00000196074	SYCP2	10388	-3.3929216	2.62E-29
ENSG00000163485	ADORA1	134	-3.2531105	1.10E-14
ENSG00000001561	ENPP4	22875	-3.1878748	1.87E-07
ENSG00000178538	CA8	767	-3.1711428	3.04E-18
ENSG00000173175	ADCY5	111	-3.1511415	1.95E-05
ENSG00000221955	SLC12A8	84561	-3.102811	0.01563034
ENSG00000171798	KNDC1	85442	-2.9539705	1.53E-05
ENSG00000036530	CYP46A1	10858	-2.9277582	0.01172575
ENSG00000104381	GDAP1	54332	-2.9108012	5.93E-82
ENSG00000003987	MTMR7	9108	-2.8992559	0.00040325
ENSG00000152213	ARL11	115761	-2.8961241	0.0023529
ENSG00000205426	KRT81	3887	-2.8758801	3.41E-165
ENSG00000184845	DRD1	1812	-2.8695068	0.01172575
ENSG00000172403	SYNPO2	171024	-2.8679763	3.38E-07

ENSG00000169894	MUC3A	4584	-2.8535452	0.03907623
ENSG00000174080	CTSF	8722	-2.8008397	1.95E-06
ENSG00000176406	RIMS2	9699	-2.7670167	0.00072987
ENSG00000198960	ARMCX6	54470	-2.7630263	4.50E-23
ENSG00000136883	KIF12	113220	-2.7116108	2.44E-09
ENSG00000119946	CNNM1	26507	-2.6954029	0.00072987
ENSG00000119938	PPP1R3C	5507	-2.6806329	4.51E-15
ENSG00000130303	BST2	684	-2.6668536	0.00027786
ENSG00000243566	UPK3B	105375355	-2.6648096	1.10E-05
ENSG00000169129	AFAP1L2	84632	-2.6222188	1.22E-11
ENSG00000004838	ZMYND10	51364	-2.6113869	0.03907623
ENSG00000174827	PDZK1	5174	-2.6044798	9.35E-13
ENSG00000174469	CNTNAP2	26047	-2.6003389	1.94E-05
ENSG00000205307	SAP25	100316904	-2.5969852	0.00739204
ENSG00000178826	TMEM139	135932	-2.5750059	0.03907623
ENSG00000152527	PLEKHH2	130271	-2.5380054	8.17E-08
ENSG00000152217	SETBP1	26040	-2.5248175	0.0041855
ENSG00000240204	SMKR1	100287482	-2.5168521	4.28E-06
ENSG00000144802	NFKBIZ	64332	-2.5048187	1.49E-13
ENSG00000173531	MST1	4485	-2.4987756	6.65E-05
ENSG00000134533	RERG	85004	-2.4960921	7.20E-16
ENSG00000112562	SMOC2	64094	-2.4850427	0.00015696
ENSG00000197603	CPLANE1	65250	-2.4821743	0.00085715
ENSG00000062282	DGAT2	84649	-2.4710352	0.00739204
ENSG00000173210	ABLIM3	22885	-2.4048822	1.65E-06
ENSG00000151612	ZNF827	152485	-2.4015623	0.0075462
ENSG00000143847	PPFIA4	8497	-2.4012836	0.03859258
ENSG00000235863	B3GALT4	8705	-2.3977624	0.00011351
ENSG00000143502	SUSD4	55061	-2.3872981	0.00091258
ENSG00000185745	IFIT1	3434	-2.3680819	0.01294922
ENSG00000158747	NBL1	100532736	-2.3620851	2.41E-32
ENSG00000186086	NBPF6	653149	-2.3575305	2.68E-09
ENSG00000106123	EPHB6	2051	-2.3405927	0.02149439
ENSG00000204248	COL11A2	1302	-2.3280788	0.00149224
ENSG00000157064	NMNAT2	23057	-2.3069618	0.00053488
ENSG00000224940	PRRT4	401399	-2.3025688	0.0041855
ENSG00000166750	SLFN5	162394	-2.2935759	0.00260405
ENSG00000189221	MAOA	4128	-2.2830522	2.45E-05
ENSG00000108679	LGALS3BP	3959	-2.2800886	6.50E-59
ENSG00000007516	BAIAP3	8938	-2.2685756	7.64E-12
ENSG00000153071	DAB2	112267931	-2.252094	1.44E-05
ENSG00000178922	HYI	81888	-2.2484318	0.01273765

ENSG00000108515	ENO3	2027	-2.2115737	9.31E-06
ENSG00000154917	RAB6B	51560	-2.2074145	0.00053488
ENSG00000078018	MAP2	4133	-2.196063	0.03907623
ENSG00000165626	BEND7	222389	-2.1925999	7.60E-07
ENSG00000021300	PLEKHB1	58473	-2.1844169	1.69E-10
ENSG00000164626	KCNK5	8645	-2.1828394	0.00091457
ENSG00000137441	FGFBP2	83888	-2.1824262	0.03907623
ENSG00000075461	CACNG4	27092	-2.17963	2.56E-39
ENSG00000213928	IRF9	10379	-2.1781382	3.87E-08
ENSG00000127588	GNG13	51764	-2.1646246	0.00091258
ENSG00000113749	HRH2	3274	-2.1565352	0.03859258
ENSG00000089127	OAS1	4938	-2.1551783	0.01273765
ENSG00000163082	SGPP2	130367	-2.118093	0.03907623
ENSG00000106327	TFR2	7036	-2.1180234	5.60E-06
ENSG00000215788	TNFRSF25	8718	-2.1061504	2.06E-06
ENSG00000164742	ADCY1	107	-2.0962151	4.67E-30
ENSG00000156587	UBE2L6	9246	-2.0832865	2.08E-06
ENSG00000164850	GPER1	2852	-2.0692045	0.00019588
ENSG00000169116	PARM1	25849	-2.0666892	0.01273765
ENSG00000112796	ENPP5	59084	-2.0574782	0.0001167
ENSG00000254815	LMNTD2-AS1	692247	-2.0525192	0.02247458
ENSG00000091527	CDV3	55573	-2.0511857	4.79E-232
ENSG00000256061	DNAAF4	161582	-2.0439656	0.01273765
ENSG00000183186	C2CD4C	126567	-2.0411361	0.02247458
ENSG00000185686	PRAME	23532	-2.0203474	2.51E-05
ENSG00000143994	ABHD1	84696	-2.0170114	0.00720686
ENSG00000050628	PTGER3	5733	-2.0144791	0.02247458
ENSG00000242294	STAG3L5P	101735302	-2.0136236	6.19E-08
ENSG00000102401	ARMCX3	51566	-2.0102826	5.13E-20
ENSG00000114923	SLC4A3	6508	-2.0070808	0.0003252
ENSG00000106624	AEBP1	165	-2.0048843	0.01183117
ENSG00000127863	TNFRSF19	55504	-1.9913273	0.00260405
ENSG00000130203	APOE	348	-1.9896904	0.0001167
ENSG00000154914	USP43	124739	-1.9787014	6.19E-08
ENSG00000129295	DNAAF11	23639	-1.9701546	0.03517795
ENSG00000125510	OPRL1	4987	-1.963984	0.00091457
ENSG00000101104	PABPC1L	80336	-1.9591749	4.15E-07
ENSG00000004399	PLXND1	23129	-1.9554482	2.57E-17
ENSG00000130600	H19	102724852	-1.9506304	2.40E-05
ENSG00000245750	DRAIC	145837	-1.9382728	0.00435016
ENSG00000151090	THRB	7068	-1.9329747	3.62E-11
ENSG00000183840	GPR39	2863	-1.9313517	0.01922887

ENSG00000185507	IRF7	3665	-1.9211478	6.61E-05
ENSG00000196427	NBPF4	148545	-1.9190173	6.16E-14
ENSG00000004948	CALCR	799	-1.9157188	0.03859258
ENSG00000159445	THEM4	117145	-1.90686	6.63E-07
ENSG00000170379	TCAF2	285966	-1.8982126	3.61E-05
ENSG00000164877	MICALL2	79778	-1.8978343	1.97E-18
ENSG00000108821	COL1A1	1277	-1.8870287	7.41E-12
ENSG00000121005	CRISPLD1	83690	-1.8865942	6.21E-05
ENSG00000196353	CPNE4	131034	-1.8840924	0.00143458
ENSG00000177519	RPRM	56475	-1.8831215	0.00720686
ENSG00000111981	ULBP1	80329	-1.8804457	0.03859258
ENSG00000161905	ALOX15	246	-1.8693707	3.76E-13
ENSG00000099338	CATSPERG	57828	-1.8567088	0.02128703
ENSG00000105204	DYRK1B	9149	-1.8556722	2.99E-08
ENSG00000145911	N4BP3	23138	-1.8534996	1.10E-93
ENSG00000167646	DNAAF3	352909	-1.8520815	0.002321
ENSG00000247746	USP51	158880	-1.8370544	0.01183117
ENSG00000103723	AP3B2	8120	-1.8365005	0.00372671
ENSG00000204642	HLA-F	3134	-1.8305604	0.002321
ENSG00000150995	ITPR1	3708	-1.8256239	9.04E-07
ENSG00000266714	MYO15B	80022	-1.8188936	4.47E-14
ENSG00000240024	LINC00888	100505687	-1.8115983	0.00082401
ENSG00000155367	PPM1J	333926	-1.8084209	0.00593517
ENSG00000123119	NECAB1	64168	-1.8044779	4.55E-26
ENSG00000176809	LRRC37A3	374819	-1.8031128	3.03E-10
ENSG00000186648	CARMIL3	90668	-1.8015622	0.00593517
ENSG00000163040	CCDC74A	90557	-1.7976043	0.00372671
ENSG00000141424	SLC39A6	25800	-1.7972992	1.25E-156
ENSG00000004777	ARHGAP33	115703	-1.7971231	0.00017101
ENSG00000153814	JAZF1	221895	-1.7956882	0.03517795
ENSG00000055118	KCNH2	3757	-1.7955095	9.08E-05
ENSG00000255346	NOX5	79400	-1.7921791	0.00082401
ENSG00000132688	NES	10763	-1.7918842	0.00132252
ENSG00000163513	TGFBR2	7048	-1.7864	0.00662094
ENSG00000076555	ACACB	32	-1.7788998	3.09E-30
ENSG00000168000	BSCL2	26580	-1.7631451	6.25E-11
ENSG00000116117	PARD3B	117583	-1.7616841	0.00038993
ENSG00000136237	RAPGEF5	9771	-1.761248	0.00662094
ENSG00000114812	VIPR1	7433	-1.758125	0.00188365
ENSG00000128604	IRF5	3663	-1.7569758	0.00068219
ENSG00000173852	DPY19L1	23333	-1.7515429	3.86E-13
ENSG00000110318	CEP126	57562	-1.7512438	0.03517795

ENSG00000071246	VASH1	22846	-1.747896	0.00014281
ENSG00000177943	MAMDC4	158056	-1.7384083	0.00662094
ENSG00000149927	DOC2A	8448	-1.7269718	6.78E-07
ENSG00000165548	TMEM63C	57156	-1.7239093	0.00024833
ENSG00000258839	MC1R	4157	-1.7199064	1.16E-06
ENSG00000014914	MTMR11	10903	-1.7160244	2.74E-05
ENSG00000113763	UNC5A	90249	-1.7129947	0.00662094
ENSG00000108551	RASD1	51655	-1.7017171	2.74E-05
ENSG00000188833	ENTPD8	377841	-1.6986565	0.04907249
ENSG00000006071	ABCC8	6833	-1.6906205	0.01922887
ENSG00000150556	LYPD6B	130576	-1.6885134	0.00294266
ENSG00000167525	PROCA1	147011	-1.6884627	0.01691965
ENSG00000123999	INHA	3623	-1.6833614	0.00593517
ENSG00000006047	YBX2	51087	-1.6822955	2.58E-11
ENSG00000165795	NDRG2	57447	-1.675122	6.78E-07
ENSG00000196739	COL27A1	85301	-1.6709216	7.39E-05
ENSG00000067840	PDZD4	57595	-1.6662636	1.27E-05
ENSG00000137198	GMPR	2766	-1.6651894	0.00294266
ENSG00000204740	MALRD1	340895	-1.6642681	3.79E-05
ENSG00000126709	IFI6	2537	-1.6577032	0.01691965
ENSG00000105290	APLP1	333	-1.6553476	2.31E-07
ENSG00000037042	TUBG2	27175	-1.652913	4.26E-05
ENSG00000275793	RIMBP3	85376	-1.6515566	0.0226817
ENSG00000179148	ALOXE3	59344	-1.6489067	0.03517795
ENSG00000127415	IDUA	3425	-1.6458627	2.77E-06
ENSG00000075213	SEMA3A	10371	-1.6440795	0.01691965
ENSG00000003989	SLC7A2	6542	-1.640688	6.40E-72
ENSG00000150551	LYPD1	116372	-1.6392636	0.01691965
ENSG00000100767	PAPLN	89932	-1.6294529	0.00294266
ENSG00000087085	ACHE	43	-1.6287203	0.04907249
ENSG00000105711	SCN1B	6324	-1.61994	0.00814464
ENSG00000168386	FILIP1L	11259	-1.6184757	0.00145564
ENSG00000250067	YJEFN3	374887	-1.6061462	0.03090773
ENSG00000115896	PLCL1	5334	-1.6060266	0.00166338
ENSG00000214021	TTLL3	26140	-1.598839	4.30E-07
ENSG00000179304	FAM156B	727866	-1.5958185	0.01691965
ENSG00000215769	ARHGAP27P1-BPTFP1-KPNA2P3	109286553	-1.591626	0.00456191
ENSG00000072778	ACADVL	37	-1.5861075	2.82E-47
ENSG00000102878	HSF4	3299	-1.5854794	8.91E-05
ENSG00000156049	GNA14	9630	-1.5787015	0.00701472
ENSG00000139182	CLSTN3	9746	-1.5786613	1.77E-09

ENSG00000113448	PDE4D	5144	-1.5701372	0.00294266
ENSG00000245532	NEAT1	283131	-1.5688294	3.80E-14
ENSG00000113108	APBB3	10307	-1.5677863	0.00222859
ENSG00000111341	MGP	4256	-1.5636654	0.00015494
ENSG00000105088	OLFM2	93145	-1.5592024	3.92E-05
ENSG00000121716	PILRB	29990	-1.5539829	2.79E-13
ENSG00000114670	NEK11	79858	-1.5528742	0.00011797
ENSG00000009950	MLXIPL	51085	-1.5527051	0.0001355
ENSG00000072858	SIDT1	54847	-1.552149	8.39E-16
ENSG00000101115	SALL4	57167	-1.5510464	2.92E-06
ENSG00000136367	ZFHX2	85446	-1.5484334	0.00600204
ENSG00000072195	SPEG	10290	-1.547582	0.01256056
ENSG00000110844	PRPF40B	25766	-1.5469674	6.65E-08
ENSG00000160188	RSPH1	89765	-1.5424158	0.02662897
ENSG00000137819	PAQR5	54852	-1.5415944	0.00814464
ENSG00000223705	NSUN5P1	155400	-1.5397872	1.05E-08
ENSG00000160469	BRSK1	84446	-1.5296307	0.00600204
ENSG00000128596	CCDC136	64753	-1.5277813	0.01465289
ENSG00000122971	ACADS	35	-1.521753	1.97E-05
ENSG00000146555	SDK1	221935	-1.519888	0.00062419
ENSG00000101096	NFATC2	4773	-1.5195594	9.42E-29
ENSG00000105808	RASA4	10156	-1.5176788	3.05E-14
ENSG00000134824	FADS2	9415	-1.5157327	4.98E-19
ENSG00000164638	SLC29A4	222962	-1.5142992	0.01465289
ENSG00000173209	AHSA2P	130872	-1.5120859	4.04E-09
ENSG00000215018	COL28A1	340267	-1.5024576	0.02662897
ENSG00000180176	TH	7054	-1.4999865	0.04907249
ENSG00000282458	WASH5P	375690	-1.4988629	6.80E-05
ENSG00000240771	ARHGEF25	115557	-1.4967047	2.96E-05
ENSG00000232434	AJM1	389813	-1.493589	0.00905983
ENSG00000140718	FTO	79068	-1.4929932	1.99E-30
ENSG00000108846	ABCC3	8714	-1.4908434	1.88E-10
ENSG00000255052	FAM66D	112268389	-1.4899713	0.0226817
ENSG00000178498	DTX3	196403	-1.4898843	7.14E-10
ENSG00000188747	NOXA1	10811	-1.4882627	0.01615018
ENSG00000168010	ATG16L2	89849	-1.4882316	0.00046273
ENSG00000100968	NFATC4	4776	-1.4874698	0.01256056
ENSG00000160111	CPAMD8	27151	-1.4833235	0.00394342
ENSG00000128567	PODXL	5420	-1.4822116	1.51E-26
ENSG00000198753	PLXNB3	5365	-1.4815299	2.20E-13
ENSG00000130635	COL5A1	1289	-1.475653	7.46E-07
ENSG00000108932	SLC16A6	9120	-1.4737923	0.0008064

ENSG00000163584	RPL22L1	200916	-1.4693559	3.50E-18
ENSG00000272752	STAG3L5P-PVRIG2P-PILRB	101752399	-1.4682966	3.72E-13
ENSG00000169599	NFU1	27247	-1.4664246	5.01E-06
ENSG00000104093	DMXL2	23312	-1.4633023	9.02E-31
ENSG00000143653	SCCPDH	51097	-1.4627861	8.85E-05
ENSG00000176532	PRR15	222171	-1.4594213	1.49E-07
ENSG00000179240	GVQW3	100506127	-1.454266	0.0009406
ENSG00000108828	VAT1	10493	-1.4502839	5.22E-23
ENSG00000183508	TENT5C	54855	-1.4479126	1.69E-08
ENSG00000144452	ABCA12	26154	-1.4466828	5.69E-14
ENSG00000006756	ARSD	414	-1.4458566	4.49E-18
ENSG00000101850	GPR143	4935	-1.4447084	0.03472297
ENSG00000167889	MGAT5B	146664	-1.4394203	0.00046273
ENSG00000105255	FSD1	79187	-1.43883	0.00905983
ENSG00000161791	FMNL3	91010	-1.4377832	0.0009406
ENSG00000149243	KLHL35	283212	-1.4372332	0.00033902
ENSG00000154310	TNIK	23043	-1.4359912	0.03969922
ENSG00000144476	ACKR3	57007	-1.4324235	7.49E-20
ENSG00000188818	ZDHHC11	79844	-1.429692	0.01918314
ENSG00000104899	AMH	268	-1.4292571	0.02899262
ENSG00000125968	ID1	3397	-1.4271376	1.63E-05
ENSG00000170667	RASA4B	100271927	-1.4248407	2.98E-09
ENSG00000261052	SULT1A3	6818	-1.4239327	0.01147417
ENSG00000155265	GOLGA7B	401647	-1.4198491	0.00433664
ENSG00000115525	ST3GAL5	8869	-1.4167339	0.0106931
ENSG00000143387	CTSK	1513	-1.4148242	0.04142196
ENSG00000175984	DENND2C	163259	-1.4143754	4.82E-05
ENSG00000206530	CFAP44	55779	-1.4119977	0.00764991
ENSG00000179406	LINC00174	285908	-1.4090118	0.00039656
ENSG00000163462	TRIM46	80128	-1.4062823	0.01355555
ENSG00000163638	ADAMTS9	56999	-1.405687	0.00177681
ENSG00000166073	GPR176	11245	-1.4052462	0.01355555
ENSG00000104369	JPH1	56704	-1.4050516	1.06E-07
ENSG00000100867	DHRS2	10202	-1.3997689	1.04E-06
ENSG00000204934	ATP6V0E2-AS1	401431	-1.3986808	0.03472297
ENSG00000130558	OLFM1	10439	-1.3986487	1.43E-09
ENSG00000185621	LMLN	89782	-1.3976168	0.00011327
ENSG00000141258	SGSM2	9905	-1.3971079	1.47E-37
ENSG00000168970	JMJD7-PLA2G4B	8681	-1.3906251	0.01918314
ENSG00000214193	SH3D21	79729	-1.3887785	0.01135448
ENSG00000163393	SLC22A15	55356	-1.386993	7.08E-05
ENSG00000079308	TNS1	7145	-1.3854333	0.00433664

ENSG00000160781	PAQR6	79957	-1.3842384	0.03472297
ENSG00000115616	SLC9A2	6549	-1.3841686	8.89E-07
ENSG00000101210	EEF1A2	1917	-1.3707157	3.39E-68
ENSG00000217555	CKLF	51192	-1.3696794	0.00541742
ENSG00000075275	CELSR1	9620	-1.3686746	9.66E-31
ENSG00000240303	ACAD11	84129	-1.3649264	1.88E-06
ENSG00000205517	RGL3	57139	-1.3649146	0.02899262
ENSG00000205560	CPT1B	1375	-1.3611795	0.00058784
ENSG00000127946	HIP1	3092	-1.3531027	1.08E-13
ENSG00000184860	SDR42E1	93517	-1.3520166	0.00644346
ENSG00000106976	DNM1	1759	-1.34745	0.00020946
ENSG00000235194	PPP1R3E	90673	-1.3429559	0.00073425
ENSG00000130813	SHFL	55337	-1.3392541	0.00086653
ENSG00000169992	NLGN2	57555	-1.3378578	2.09E-14
ENSG00000164919	COX6C	1345	-1.3375718	1.29E-81
ENSG00000135722	FBXL8	55336	-1.3375258	0.0167043
ENSG00000167925	GHDC	84514	-1.333847	2.33E-06
ENSG00000159200	RCAN1	1827	-1.3320762	7.55E-08
ENSG00000158457	TSPAN33	340348	-1.330881	0.04332723
ENSG00000187608	ISG15	9636	-1.3236632	6.69E-06
ENSG00000213920	MDP1	145553	-1.3227353	0.00042503
ENSG00000134759	ELP2	55250	-1.3210571	9.28E-43
ENSG00000083457	ITGAE	3682	-1.3185917	5.38E-05
ENSG00000215252	GOLGA8B	440270	-1.3171431	0.00185314
ENSG00000184557	SOCS3	9021	-1.3114338	0.00010829
ENSG00000162004	CCDC78	124093	-1.3112835	9.79E-09
ENSG00000160602	NEK8	284086	-1.310248	7.21E-07
ENSG00000162636	FAM102B	284611	-1.3096841	4.66E-53
ENSG00000142173	COL6A2	1292	-1.3080961	0.00662829
ENSG00000139192	TAPBPL	55080	-1.3078627	0.00018485
ENSG00000138061	CYP1B1	1545	-1.3072904	4.13E-66
ENSG00000156103	MMP16	4325	-1.3065126	9.95E-07
ENSG00000128849	CGNL1	84952	-1.3028577	0.02899262
ENSG00000170917	NUDT6	11162	-1.30175	0.00127001
ENSG00000130508	PXDN	7837	-1.3012772	1.17E-16
ENSG00000170382	LRRN2	10446	-1.296382	0.00127001
ENSG00000124104	SNX21	90203	-1.2962917	0.00010829
ENSG00000182796	TMEM198B	440104	-1.2955828	0.00031561
ENSG00000213859	KCTD11	147040	-1.2949343	9.25E-08
ENSG00000142632	ARHGEF19	128272	-1.2946237	0.00062133
ENSG00000117983	MUC5B	727897	-1.2885195	8.52E-23
ENSG00000166681	BEX3	27018	-1.2873649	1.09E-05

ENSG00000117016	RIMS3	9783	-1.2839636	0.02434528
ENSG00000272031	ANKRD34A	284615	-1.2796917	6.02E-10
ENSG00000187848	P2RX2	22953	-1.2793106	0.02434528
ENSG00000232859	LYRM9	201229	-1.2789716	0.04282405
ENSG00000184619	KRBA2	124751	-1.2788675	0.02899262
ENSG00000106479	ZNF862	643641	-1.2787778	1.25E-06
ENSG00000248487	ABHD14A	25864	-1.2783471	0.00793678
ENSG00000251136	RIPK2-DT	101929709	-1.2742905	0.00053891
ENSG00000179029	TMEM107	84314	-1.2733963	0.0009034
ENSG00000185046	ANKS1B	56899	-1.2631304	0.01388184
ENSG00000154118	JPH3	57338	-1.2619477	0.0167043
ENSG00000171680	PLEKHG5	57449	-1.2618081	7.45E-07
ENSG00000174963	ZIC4	84107	-1.2595486	0.0357396
ENSG00000182749	PAQR7	164091	-1.2593193	0.00155508
ENSG00000276547	PCDHGB5	56101	-1.2588762	2.94E-06
ENSG00000115935	WIPF1	7456	-1.2555734	2.53E-06
ENSG00000197415	VEPH1	79674	-1.2500471	0.02415234
ENSG00000119681	LTBP2	4053	-1.2493359	0.00015882
ENSG00000109436	TBC1D9	23158	-1.2491357	5.14E-30
ENSG00000161010	MRNIP	51149	-1.248776	4.23E-08
ENSG00000204262	COL5A2	1290	-1.2481324	1.28E-06
ENSG00000131015	ULBP2	80328	-1.2450243	0.01388184
ENSG00000185010	F8	2157	-1.2449337	0.00385349
ENSG00000197168	NEK5	341676	-1.2431347	0.04332723
ENSG00000167371	PRRT2	112476	-1.2396455	0.04332723
ENSG00000076351	SLC46A1	113235	-1.237659	1.40E-07
ENSG00000166816	LDHD	197257	-1.2337211	5.53E-05
ENSG00000123096	SSPN	8082	-1.233196	7.05E-06
ENSG00000124507	PACSIN1	29993	-1.2304585	3.35E-05
ENSG00000105519	CAPS	828	-1.2298012	0.02948972
ENSG00000147041	SYTL5	94122	-1.2285105	5.74E-09
ENSG00000154265	ABCA5	23461	-1.224268	0.0066221
ENSG00000161958	FGF11	2256	-1.2224431	0.00157146
ENSG00000175265	GOLGA8A	23015	-1.2217959	0.00412449
ENSG00000163697	APBB2	323	-1.2177736	1.92E-23
ENSG00000186417	GLDN	342035	-1.2125526	0.00662829
ENSG00000161996	WDR90	197335	-1.2122251	0.00045532
ENSG00000168280	KIF5C	3800	-1.2118057	0.00796621
ENSG00000262943	ALOX12P2	245	-1.2089791	0.04282405
ENSG00000174567	GOLT1A	127845	-1.2077662	0.00553323
ENSG00000143845	ETNK2	55224	-1.2045208	2.23E-08
ENSG00000197183	NOL4L	140688	-1.204513	2.56E-06

ENSG00000100478	AP4S1	11154	-1.2022751	0.01138179
ENSG00000145569	OTULINL	54491	-1.2019748	0.00053923
ENSG00000243701	DUBR	344595	-1.1988231	0.00321544
ENSG00000167740	CYB5D2	124936	-1.1984068	1.59E-08
ENSG00000080823	MOK	5891	-1.1980543	0.00958521
ENSG00000101213	PTK6	5753	-1.1965671	6.56E-07
ENSG00000254505	CHMP4A	29082	-1.1940154	5.54E-07
ENSG00000198892	SHISA4	149345	-1.1910923	0.02948972
ENSG00000178971	CTC1	80169	-1.1840419	2.36E-05
ENSG00000214029	ZNF891	101060200	-1.1804876	0.02374648
ENSG00000163596	ICA1L	130026	-1.1796791	0.0066221
ENSG00000107537	PHYH	5264	-1.1771313	6.88E-09
ENSG00000197774	EME2	197342	-1.1755576	1.83E-11
ENSG00000171346	KRT15	3866	-1.1733972	0.00268307
ENSG00000241472	PTPRG-AS1	100506994	-1.1672644	0.00223898
ENSG00000106404	CLDN15	24146	-1.1668843	0.0009206
ENSG00000261115	TMEM178B	100507421	-1.1666482	0.00032002
ENSG00000005249	PRKAR2B	5577	-1.166579	2.22E-08
ENSG00000132535	DLG4	1742	-1.1649866	2.00E-05
ENSG00000139625	MAP3K12	7786	-1.163733	0.00011105
ENSG00000151892	GFRA1	2674	-1.1629914	2.08E-61
ENSG00000108947	EFNB3	1949	-1.1624435	2.56E-06
ENSG00000131370	SH3BP5	9467	-1.1610131	1.23E-39
ENSG00000261801	LOXL1-AS1	100287616	-1.1599425	0.02011072
ENSG00000135899	SP110	3431	-1.1594573	0.00647926
ENSG00000138613	APH1B	83464	-1.1548179	0.00018533
ENSG00000105707	HPN	3249	-1.1540137	0.02374648
ENSG00000196405	EVL	51466	-1.1506365	2.25E-55
ENSG00000132359	RAP1GAP2	23108	-1.1504818	4.13E-09
ENSG00000157992	KRTCAP3	200634	-1.1473009	0.01331877
ENSG00000196976	LAGE3	8270	-1.1467021	1.64E-11
ENSG00000174945	AMZ1	155185	-1.1448903	2.36E-05
ENSG00000173227	SYT12	91683	-1.1430393	5.98E-21
ENSG00000112541	PDE10A	90632	-1.1421952	0.02758429
ENSG00000120738	EGR1	1958	-1.1407625	7.80E-05
ENSG00000160191	PDE9A	5152	-1.1379993	0.00647926
ENSG00000196189	SEMA4A	64218	-1.1376125	3.58E-08
ENSG00000235750	KIAA0040	9674	-1.1354824	2.35E-10
ENSG00000174292	TNK1	8711	-1.129586	6.84E-06
ENSG00000105613	MAST1	22983	-1.1265276	0.04101652
ENSG00000174282	ZBTB4	57659	-1.1257941	9.08E-18
ENSG00000185101	ANO9	338440	-1.1256882	0.0026399

ENSG00000131171	SH3BGRL	6451	-1.1242232	1.54E-21
ENSG00000108852	MPP2	4355	-1.1240328	0.00370751
ENSG00000213983	AP1G2	8906	-1.1227438	6.50E-16
ENSG00000165821	SALL2	6297	-1.1212599	0.00061575
ENSG00000180694	TMEM64	169200	-1.1197652	9.50E-51
ENSG00000159176	CSRP1	1465	-1.119238	2.57E-28
ENSG00000198719	DLL1	28514	-1.1175411	0.02374648
ENSG00000104081	BMF	90427	-1.1175217	1.23E-06
ENSG00000162194	LBHD1	79081	-1.1165945	0.00308114
ENSG00000271122	HERPUD2-AS1	101930085	-1.1136583	5.36E-05
ENSG00000149557	FEZ1	105369550	-1.1125273	0.00942714
ENSG00000134285	FKBP11	51303	-1.1110885	0.00029931
ENSG00000106333	PCOLCE	5118	-1.110906	8.85E-05
ENSG00000143126	CELSR2	1952	-1.1092886	2.29E-41
ENSG00000204516	MICB	4277	-1.1077417	0.00910332
ENSG00000146830	GIGYF1	64599	-1.107721	6.34E-18
ENSG00000105963	ADAP1	11033	-1.1075803	4.69E-06
ENSG00000137801	THBS1	7057	-1.107019	1.68E-34
ENSG00000108509	CAMTA2	23125	-1.1064262	1.54E-10
ENSG00000058404	CAMK2B	816	-1.1023725	0.00256179
ENSG00000248932	COPB2-DT	100507291	-1.1007766	0.02758429
ENSG00000116661	FBXO2	26232	-1.0979831	0.02758429
ENSG00000139998	RAB15	376267	-1.0979452	2.43E-11
ENSG00000108465	CDK5RAP3	80279	-1.0967195	1.85E-15
ENSG00000198879	SFMBT2	57713	-1.0951858	6.66E-06
ENSG00000197283	SYNGAP1	8831	-1.0929426	1.41E-08
ENSG00000182379	NXPH4	11247	-1.0927344	0.00177329
ENSG00000178726	THBD	7056	-1.0906693	0.00295923
ENSG00000141503	MINK1	50488	-1.0905108	6.74E-16
ENSG00000110375	UPK2	7379	-1.0892743	0.00517494
ENSG00000135164	DMTF1	9988	-1.0890037	1.67E-08
ENSG00000163704	PRRT3	285368	-1.0886093	7.07E-09
ENSG00000112320	SOBP	55084	-1.0860819	0.02758429
ENSG00000160323	ADAMTS13	11093	-1.0819275	0.01100626
ENSG00000186364	NUDT17	200035	-1.0771743	0.0059444
ENSG00000105875	WDR91	29062	-1.0765364	0.01048947
ENSG00000156050	FAM161B	145483	-1.0745159	0.00061575
ENSG00000165801	ARHGEF40	55701	-1.0732917	0.01048947
ENSG00000174353	STAG3L3	378108	-1.071189	0.00027525
ENSG00000131650	KREMEN2	79412	-1.0711082	0.00028793
ENSG00000104823	ECH1	1891	-1.0690012	6.76E-30
ENSG00000100889	PCK2	5106	-1.0686723	8.98E-10

ENSG00000164855	TMEM184A	202915	-1.0654377	5.97E-15
ENSG00000162782	TDRD5	163589	-1.0650952	0.00117794
ENSG00000238105	GOLGA2P5	55592	-1.0640151	0.00867309
ENSG00000160182	TFF1	7031	-1.0624939	8.14E-50
ENSG00000196689	TRPV1	7442	-1.060528	0.02758429
ENSG00000180626	ZNF594	84622	-1.0585687	0.00356315
ENSG00000171033	PKIA	5569	-1.0573701	0.00295923
ENSG00000167123	CERCAM	51148	-1.0565683	1.59E-13
ENSG00000108352	RAPGEFL1	51195	-1.0560787	0.02595114
ENSG00000132793	LPIN3	64900	-1.055189	5.99E-13
ENSG00000106351	AGFG2	3268	-1.0540609	2.06E-05
ENSG00000160991	ORAI2	80228	-1.0521074	1.75E-11
ENSG00000102390	PBDC1	51260	-1.0518086	0.00056792
ENSG00000143412	ANXA9	8416	-1.0506024	9.58E-14
ENSG00000074416	MGLL	11343	-1.0497529	0.00429277
ENSG00000183718	TRIM52	84851	-1.0494351	0.00112037
ENSG00000114626	ABTB1	80325	-1.0486575	4.80E-05
ENSG00000277363	SRCIN1	80725	-1.0468654	0.00492681
ENSG00000127990	SGCE	8910	-1.0440054	5.70E-10
ENSG00000123384	LRP1	4035	-1.0435888	1.99E-06
ENSG00000120594	PLXDC2	84898	-1.0426738	0.00117794
ENSG00000035862	TIMP2	7077	-1.0412261	2.37E-06
ENSG00000220205	VAMP2	6844	-1.0404505	0.00088211
ENSG00000170006	TMEM154	201799	-1.0380945	0.02134525
ENSG00000113971	NPHP3	27031	-1.0377093	0.00073498
ENSG00000143882	ATP6V1C2	245973	-1.0366754	0.01048947
ENSG00000198556	ZNF789	285989	-1.0352138	0.03853984
ENSG00000107186	MPDZ	8777	-1.033452	0.00112037
ENSG00000171160	MORN4	118812	-1.0315376	0.03853984
ENSG00000123570	RAB9B	51209	-1.0311598	0.04362974
ENSG00000151611	MMAA	166785	-1.0291774	0.01538784
ENSG00000198712	MT-CO2	4513	-1.0282477	6.58E-14
ENSG00000270276	H4C15	554313	-1.0230405	2.70E-06
ENSG00000111816	FRK	2444	-1.0222345	0.00465033
ENSG00000146540	C7orf50	84310	-1.021405	9.51E-18
ENSG00000161921	CXCL16	58191	-1.0197534	4.58E-06
ENSG00000186088	GSAP	54103	-1.0196469	0.02134525
ENSG00000188372	ZP3	7784	-1.0194579	2.69E-05
ENSG00000103642	LACTB	114294	-1.0173607	0.00012737
ENSG00000138028	CGREF1	10669	-1.0168964	9.50E-10
ENSG00000197594	ENPP1	5167	-1.0133893	0.0003757
ENSG00000161265	U2AF1L4	199746	-1.0096876	0.04709685

ENSG00000134107	BHLHE40	8553	-1.0080497	3.45E-11
ENSG00000265479	DTX2P1-UPK3BP1-PMS2P11	441263	-1.0075374	0.00717742
ENSG00000157326	DHRS4	10901	-1.0060252	3.64E-05
ENSG00000071282	LMCD1	29995	-1.0057237	8.35E-12
ENSG00000146826	TRAPPC14	55262	-1.0050247	3.64E-05
ENSG00000129566	TEP1	7011	-1.0024152	4.21E-07
ENSG00000187630	DHRS4L2	317749	-1.0006123	0.0006126
ENSG00000257315	ZBED6	100381270	-1.0001441	2.09E-16
ENSG00000106477	CEP41	95681	-1.00001	0.01639734
ENSG00000258366	RTEL1	51750	-0.9993086	0.0032023
ENSG00000186868	MAPT	4137	-0.9977551	4.59E-09
ENSG00000198133	TMEM229B	161145	-0.9926496	2.81E-07
ENSG00000197948	FCHSD1	89848	-0.9884615	0.00027947
ENSG00000188878	FBF1	85302	-0.9873195	0.0135343
ENSG00000186472	PCLO	27445	-0.9847358	0.01254867
ENSG00000119636	BBOF1	80127	-0.9837078	0.0315984
ENSG00000162777	DENND2D	79961	-0.9824545	7.43E-11
ENSG00000169093	ASMTL	8623	-0.981595	1.60E-05
ENSG00000131558	EXOC4	60412	-0.9804622	5.09E-12
ENSG00000251562	MALAT1	378938	-0.9776248	8.17E-06
ENSG00000221968	FADS3	3995	-0.9761026	0.01037949
ENSG00000128513	POT1	25913	-0.975861	0.00161424
ENSG00000104320	NBN	4683	-0.9754872	3.94E-24
ENSG00000143554	SLC27A3	11000	-0.9739219	4.14E-08
ENSG00000196923	PDLIM7	9260	-0.9737139	8.72E-10
ENSG00000185324	CDK10	8558	-0.9734677	8.50E-07
ENSG00000183401	CCDC159	126075	-0.9731613	0.01639734
ENSG00000175455	CCDC14	64770	-0.9718295	1.44E-06
ENSG00000117115	PADI2	11240	-0.9716845	0.00435929
ENSG00000069020	MAST4	375449	-0.9707163	0.00069161
ENSG00000074370	ATP2A3	489	-0.9693949	6.86E-44
ENSG00000148180	GSN	2934	-0.9688526	3.91E-10
ENSG00000196507	TCEAL3	85012	-0.968808	1.71E-06
ENSG00000100558	PLEK2	26499	-0.968725	0.01118238
ENSG00000152926	ZNF117	109504726	-0.9680572	0.00150321
ENSG00000155100	OTUD6B	51633	-0.9671784	2.29E-06
ENSG00000245694	CRNDE	101927480	-0.9643158	3.25E-06
ENSG00000196220	SRGAP3	9901	-0.9638323	0.00711834
ENSG00000176244	ACBD7	414149	-0.9620063	5.49E-05
ENSG00000161249	DMKN	93099	-0.9615308	1.10E-07
ENSG00000109046	WSB1	26118	-0.9612785	5.87E-06

ENSG00000012171	SEMA3B	7869	-0.960957	0.00026232
ENSG00000010361	FUZ	80199	-0.9589079	0.00313456
ENSG00000167595	PROSER3	148137	-0.9583128	0.03010198
ENSG00000170310	STX8	9482	-0.9566221	0.00634012
ENSG00000100911	PSME2	5721	-0.9564557	7.27E-10
ENSG00000245060	LINC00847	729678	-0.9561939	8.35E-06
ENSG00000112297	CRYBG1	202	-0.9532494	5.47E-09
ENSG00000069966	GNB5	10681	-0.9513084	5.29E-07
ENSG00000168461	RAB31	11031	-0.9501847	3.63E-06
ENSG00000185561	TLCD2	727910	-0.950091	0.01518474
ENSG00000163660	CCNL1	57018	-0.9478141	2.13E-08
ENSG00000132563	REEP2	51308	-0.9467063	0.03576482
ENSG00000116299	ELAPOR1	57535	-0.9452047	7.56E-55
ENSG00000152518	ZFP36L2	678	-0.944444	1.54E-36
ENSG00000270882	H4C14	8370	-0.9423164	1.29E-07
ENSG00000099949	LZTR1	8216	-0.9413217	0.01531348
ENSG00000176623	RMDN1	51115	-0.9411914	2.65E-12
ENSG00000157388	CACNA1D	776	-0.9410249	1.69E-12
ENSG00000160766	GBAP1	2630	-0.9402288	0.02707373
ENSG00000156535	CD109	135228	-0.9397219	0.00590135
ENSG00000204681	GABBR1	2550	-0.9395111	0.01988795
ENSG00000235706	DICER1-AS1	400242	-0.9391098	0.01988795
ENSG00000135622	SEMA4F	10505	-0.9366505	0.00731364
ENSG00000007168	PAFAH1B1	5048	-0.9362883	2.40E-13
ENSG00000197261	C6orf141	135398	-0.9358399	0.00590135
ENSG00000197417	SHPK	23729	-0.9350604	3.01E-05
ENSG00000104325	DECR1	1666	-0.934498	7.20E-11
ENSG00000170153	RNF150	57484	-0.9332918	0.01872011
ENSG00000100167	SEPTIN3	55964	-0.9326968	0.04006152
ENSG00000005189	REXO5	81691	-0.9318824	0.01518474
ENSG00000146904	EPHA1	2041	-0.928214	6.55E-05
ENSG00000130827	PLXNA3	55558	-0.9278876	3.06E-12
ENSG00000155016	CYP2U1	113612	-0.9268077	0.02707373
ENSG00000077454	LRCH4	4034	-0.9262333	1.19E-13
ENSG00000243335	KCTD7	154881	-0.9260949	0.03322491
ENSG00000107201	DDX58	23586	-0.9260176	0.04006152
ENSG00000239521	CASTOR3	352954	-0.9252288	0.00139498
ENSG00000171444	MCC	4163	-0.9249686	0.00290028
ENSG00000101577	LPIN2	9663	-0.9242492	0.00033809
ENSG00000186104	CYP2R1	120227	-0.9211238	0.01037949
ENSG00000162545	CAMK2N1	55450	-0.9206881	1.83E-05
ENSG00000143320	CRABP2	1382	-0.9203455	7.56E-23

ENSG00000081307	UBA5	79876	-0.9193689	5.40E-09
ENSG00000089723	OTUB2	78990	-0.9178554	0.02481345
ENSG00000187838	PLSCR3	57048	-0.9175517	6.59E-06
ENSG00000141499	WRAP53	55135	-0.91706	0.00463941
ENSG00000160796	NBEAL2	23218	-0.9166659	9.15E-19
ENSG00000139631	CSAD	51380	-0.9153319	0.00607217
ENSG00000103544	VPS35L	57020	-0.9148293	0.04904902
ENSG00000164951	PDP1	54704	-0.9140747	0.00099428
ENSG00000184307	ZDHHC23	254887	-0.9139659	2.03E-05
ENSG00000078269	SYNJ2	8871	-0.9137361	0.00012835
ENSG00000106484	MEST	4232	-0.9129813	1.19E-09
ENSG00000171503	ETFDH	2110	-0.9122287	8.34E-05
ENSG00000171700	RGS19	10287	-0.9119775	0.00069331
ENSG00000136247	ZDHHC4	55146	-0.9110919	7.13E-10
ENSG00000100439	ABHD4	63874	-0.9110024	0.00016937
ENSG00000135473	PAN2	9924	-0.9101645	3.84E-08
ENSG00000169231	THBS3	7059	-0.9098775	2.89E-05
ENSG00000162591	MEGF6	1953	-0.9096471	0.00419245
ENSG00000072849	DERL2	51009	-0.9073364	0.00019773
ENSG00000198585	NUDT16	131870	-0.906847	3.01E-08
ENSG00000090924	PLEKHG2	64857	-0.9034129	3.04E-07
ENSG00000181409	AATK	9625	-0.9030974	0.00033809
ENSG00000146776	ATXN7L1	222255	-0.9029007	0.00671181
ENSG00000182224	CYB5D1	124637	-0.9018696	1.11E-05
ENSG00000277053	GTF2IP1	2970	-0.8992989	5.72E-13
ENSG00000077549	CAPZB	832	-0.8990544	9.74E-25
ENSG00000184939	ZFP90	146198	-0.8987385	4.00E-06
ENSG00000041880	PARP3	10039	-0.8987198	0.0035442
ENSG00000204267	TAP2	6891	-0.8981344	0.00053475
ENSG00000150967	ABCB9	23457	-0.8973983	0.00419245
ENSG00000145916	RMND5B	64777	-0.89647	4.28E-29
ENSG00000106665	CLIP2	7461	-0.8937199	0.03322491
ENSG00000111331	OAS3	4940	-0.8933305	1.85E-06
ENSG00000183011	NAA38	84316	-0.8928542	4.08E-06
ENSG00000110881	ASIC1	41	-0.8926053	0.00246095
ENSG00000131788	PIAS3	10401	-0.8907719	2.48E-15
ENSG00000173599	PC	5091	-0.8906723	9.45E-07
ENSG00000116667	C1orf21	81563	-0.8905908	4.39E-07
ENSG00000163482	STK36	27148	-0.8902733	1.36E-05
ENSG00000260280	SLX1B-SULT1A4	100526831	-0.8899875	0.03290963
ENSG00000269713	NBPF9	400818	-0.8891753	7.00E-12
ENSG00000006282	SPATA20	64847	-0.8883903	1.43E-23

ENSG00000086300	SNX10	29887	-0.887346	1.85E-06
ENSG00000104783	KCNN4	3783	-0.8869556	0.00888272
ENSG00000184465	WDR27	253769	-0.8865945	0.00246095
ENSG00000215041	NEURL4	84461	-0.8864181	5.08E-05
ENSG00000067836	ROGDI	79641	-0.8860632	1.78E-05
ENSG00000058668	ATP2B4	493	-0.885153	0.00018663
ENSG00000167720	SRR	63826	-0.8841931	0.00888272
ENSG00000170037	CNTROB	116840	-0.8835903	5.01E-05
ENSG00000215271	HOMEZ	57594	-0.8798031	0.00028868
ENSG00000088367	EPB41L1	2036	-0.8791763	6.20E-07
ENSG00000130589	HELZ2	85441	-0.8790118	4.26E-05
ENSG00000167615	LENG8	114823	-0.8787543	0.00038929
ENSG00000138463	SLC49A4	84925	-0.8786726	0.01413742
ENSG00000166435	XRRA1	143570	-0.876415	0.01872011
ENSG00000117616	RSRP1	57035	-0.8763482	7.21E-05
ENSG00000156299	TIAM1	7074	-0.8744017	0.00120618
ENSG00000122783	CYREN	78996	-0.8742141	7.21E-05
ENSG00000138030	KHK	3795	-0.8740654	0.00225879
ENSG00000228253	MT-ATP8	4509	-0.8738619	1.97E-05
ENSG00000185989	RASA3	22821	-0.8733723	7.48E-07
ENSG00000184887	BTBD6	90135	-0.8728378	1.28E-10
ENSG00000103888	CEMIP	57214	-0.8720093	0.00077365
ENSG00000150593	PDCD4	27250	-0.870638	4.21E-20
ENSG00000141480	ARRB2	409	-0.8694436	1.27E-05
ENSG00000146950	SHROOM2	357	-0.8683838	0.0003488
ENSG00000128564	VGF	7425	-0.8675946	0.0035442
ENSG00000177679	SRRM3	222183	-0.867168	0.01068915
ENSG00000203880	PCMTD2	55251	-0.8656253	2.85E-09
ENSG00000204314	PRRT1	100507547	-0.8652406	0.04446836
ENSG00000114166	KAT2B	8850	-0.8646923	0.00172811
ENSG00000168067	MAP4K2	5871	-0.8641182	0.00120618
ENSG00000221909	FAM200A	221786	-0.863149	0.01548956
ENSG00000167702	KIFC2	90990	-0.8630372	5.56E-05
ENSG00000171703	TCEA2	6919	-0.8627172	9.40E-05
ENSG00000006016	CRLF1	9244	-0.8627148	0.00389482
ENSG00000056998	GYG2	8908	-0.8621885	0.04896708
ENSG00000196700	ZNF512B	57473	-0.8614677	8.78E-14
ENSG00000089820	ARHGAP4	393	-0.8602601	0.0002922
ENSG00000158623	COPG2	26958	-0.8598599	3.31E-08
ENSG00000181035	SLC25A42	284439	-0.8592219	0.03009825
ENSG00000165792	METTL17	64745	-0.8590823	4.69E-05
ENSG00000155363	MOV10	4343	-0.8569188	4.31E-12

ENSG00000168350	DEGS2	123099	-0.8565671	8.19E-13
ENSG00000160223	ICOSLG	23308	-0.8542564	0.01068915
ENSG00000164292	RHOBTB3	22836	-0.8542438	9.40E-11
ENSG00000160345	C9orf116	138162	-0.8535653	0.03285603
ENSG00000196981	WDR5B	54554	-0.8476754	0.00247827
ENSG00000187837	H1-2	3006	-0.8466719	0.00013327
ENSG00000125520	SLC2A4RG	56731	-0.8466529	4.28E-14
ENSG00000110975	SYT10	341359	-0.8466332	5.90E-07
ENSG00000204946	ZNF783	100289678	-0.8451711	0.00172811
ENSG00000233369	GTF2IP4	100093631	-0.8449076	1.11E-18
ENSG00000168778	TCTN2	79867	-0.8444732	0.0364323
ENSG00000145882	PCYOX1L	78991	-0.8436897	0.00189237
ENSG00000198786	MT-ND5	4540	-0.8426573	1.26E-07
ENSG00000187189	TSPYL4	23270	-0.8425278	2.99E-06
ENSG00000163389	POGLUT1	56983	-0.8415343	0.00560977
ENSG00000125841	NRSN2	80023	-0.8408419	0.00034996
ENSG00000263001	GTF2I	2969	-0.8402895	4.16E-40
ENSG00000156398	SFXN2	118980	-0.8401858	0.00101224
ENSG00000212907	MT-ND4L	4539	-0.8389799	0.00024456
ENSG00000124615	MOCS1	4337	-0.8372477	0.01406214
ENSG00000222011	FAM185A	222234	-0.8357308	0.00809179
ENSG00000130584	ZBTB46	140685	-0.8351667	0.01705789
ENSG00000088280	ASAP3	55616	-0.8349367	0.03285603
ENSG00000005469	CROT	54677	-0.8343996	0.00029281
ENSG00000182575	NXPH3	11248	-0.834239	0.01530055
ENSG00000146828	SLC12A9	56996	-0.832448	5.76E-11
ENSG00000134253	TRIM45	80263	-0.8320509	2.08E-09
ENSG00000197956	S100A6	6277	-0.8314902	1.76E-15
ENSG00000118513	MYB	4602	-0.8313691	5.47E-10
ENSG00000188735	TMEM120B	144404	-0.8303583	0.00800851
ENSG00000085719	CPNE3	8895	-0.8296106	6.07E-29
ENSG00000198715	GLMP	112770	-0.8268979	4.29E-05
ENSG00000172878	METAP1D	254042	-0.8258131	0.04306273
ENSG00000108518	PFN1	5216	-0.825766	1.60E-31
ENSG00000205356	TECPR1	25851	-0.8250935	0.00031837
ENSG00000180530	NRIP1	8204	-0.8248072	6.52E-08
ENSG00000114054	PCCB	5096	-0.8235066	1.26E-09
ENSG00000172264	MACROD2	140733	-0.8234928	0.02454408
ENSG00000110237	ARHGEF17	9828	-0.8231469	9.37E-05
ENSG00000138434	ITPRID2	6744	-0.8229743	4.39E-45
ENSG00000198910	L1CAM	3897	-0.8226945	0.00045121
ENSG00000174151	CYB561D1	284613	-0.8204049	4.98E-06

ENSG00000132522	GPS2	2874	-0.8203662	6.58E-05
ENSG00000156253	RWDD2B	10069	-0.8203347	0.00070825
ENSG00000029725	RABEP1	9135	-0.8200209	4.84E-11
ENSG00000123836	PFKFB2	5208	-0.8195609	0.00605884
ENSG00000126522	ASL	435	-0.8181697	9.07E-06
ENSG00000006025	OSBPL7	114881	-0.8180766	0.01286961
ENSG00000142102	PGGHG	80162	-0.8164088	0.01040523
ENSG00000230487	PSMG3-AS1	114796	-0.8148357	4.62E-05
ENSG00000149218	ENDOD1	23052	-0.8144583	0.00735811
ENSG00000100949	RABGGTA	5875	-0.8133633	0.0001209
ENSG00000005884	ITGA3	3675	-0.8115973	8.60E-09
ENSG00000215424	MCM3AP-AS1	114044	-0.8111826	0.02037437
ENSG00000105520	PLPPR2	64748	-0.8102449	7.49E-07
ENSG00000106397	PLOD3	8985	-0.8095506	8.22E-10
ENSG00000167644	C19orf33	64073	-0.8094387	3.81E-06
ENSG00000182134	TDRKH	11022	-0.8081747	1.36E-06
ENSG00000064655	EYA2	2139	-0.8081544	0.04306273
ENSG00000184220	CMSS1	84319	-0.8080308	0.00348266
ENSG00000129071	MBD4	8930	-0.8062375	3.59E-05
ENSG00000141337	ARSG	22901	-0.8059893	0.0022175
ENSG00000167971	CASKIN1	57524	-0.8056401	0.0029164
ENSG00000274070	CASTOR2	729438	-0.8055897	0.00264539
ENSG00000181222	POLR2A	5430	-0.805422	1.16E-08
ENSG00000162650	ATXN7L2	127002	-0.8050742	0.01658307
ENSG00000140104	CLBA1	122616	-0.8035803	0.00037425
ENSG00000071859	FAM50A	9130	-0.8030005	1.34E-09
ENSG00000145014	TMEM44	93109	-0.8016126	0.02454408
ENSG00000122729	ACO1	48	-0.8014244	0.00128586
ENSG00000214194	SMIM30	401397	-0.8009979	0.00041107
ENSG00000099940	SNAP29	9342	-0.8007045	0.00016821
ENSG00000162825	NBPF20	100288142	-0.8004689	0.02958417
ENSG00000171130	ATP6V0E2	155066	-0.7999663	1.94E-08
ENSG00000034533	ASTE1	28990	-0.7993779	0.04414239
ENSG00000134202	GSTM3	2947	-0.7991438	1.26E-23
ENSG00000198920	KIAA0753	9851	-0.798737	0.00383982
ENSG00000215256	DHRS4-AS1	55449	-0.7973372	0.00011029
ENSG00000225210	DUXAP9	503638	-0.7963206	0.00308092
ENSG00000146067	FAM193B	54540	-0.7953032	4.22E-05
ENSG00000114107	CEP70	80321	-0.7913871	0.01151703
ENSG00000166888	STAT6	6778	-0.7911395	2.91E-06
ENSG00000204271	SPIN3	169981	-0.7901247	0.00074825
ENSG00000176444	CLK2	1196	-0.7886751	4.69E-07

ENSG00000214944	ARHGEF28	64283	-0.7886739	0.03202564
ENSG00000185880	TRIM69	140691	-0.7882805	0.03285603
ENSG00000070444	MNT	4335	-0.7880788	0.00019573
ENSG00000183036	PCP4	5121	-0.7854452	0.03568111
ENSG00000213599	SLX1A-SULT1A3	100526830	-0.7843525	0.01344987
ENSG00000198886	MT-ND4	4538	-0.7832424	4.21E-06
ENSG00000169919	GUSB	2990	-0.7824951	9.44E-07
ENSG00000126870	DYNC2I1	55112	-0.7823017	0.01040523
ENSG00000197150	ABCB8	11194	-0.7821114	0.00233987
ENSG00000110719	TCIRG1	10312	-0.7818503	0.00656416
ENSG00000178053	MLF1	4291	-0.7816484	0.00939279
ENSG00000124357	NAGK	55577	-0.7813414	0.00017774
ENSG00000184898	RBM43	375287	-0.7801577	0.03202564
ENSG00000204764	RANBP17	64901	-0.7799853	0.02958417
ENSG00000138834	MAPK8IP3	23162	-0.7798563	1.95E-06
ENSG00000174327	SLC16A13	201232	-0.7795625	0.0464685
ENSG00000108523	RNF167	26001	-0.7791286	2.78E-08
ENSG00000142765	SYTL1	84958	-0.7785917	0.00019969
ENSG00000130702	LAMA5	3911	-0.7776989	5.27E-15
ENSG00000196781	TLE1	7088	-0.7757943	7.68E-06
ENSG00000100908	EMC9	51016	-0.7755468	0.03202564
ENSG00000166262	FAM227B	196951	-0.7740706	0.01990865
ENSG00000123358	NR4A1	3164	-0.7734956	6.04E-06
ENSG00000139722	VPS37B	79720	-0.7731012	0.00038689
ENSG00000105976	MET	4233	-0.7728818	2.85E-05
ENSG00000213462	ERV3-1	2086	-0.7727641	0.00315612
ENSG00000070366	SMG6	23293	-0.77271	2.85E-05
ENSG00000072682	P4HA2	8974	-0.7718499	0.00593506
ENSG00000114021	NIT2	56954	-0.7714675	4.56E-06
ENSG00000132394	EEFSEC	60678	-0.771393	0.00086884
ENSG00000024422	EHD2	30846	-0.7713418	0.00709095
ENSG00000133028	SCO1	6341	-0.7706252	3.06E-05
ENSG00000213977	TAX1BP3	30851	-0.7697078	1.65E-06
ENSG00000100350	FOXRED2	80020	-0.7696724	4.28E-11
ENSG00000155097	ATP6V1C1	528	-0.7695076	5.12E-11
ENSG00000137502	RAB30	27314	-0.7687179	6.76E-05
ENSG00000083857	FAT1	2195	-0.7677235	7.61E-14
ENSG00000170043	TRAPPC1	58485	-0.7676175	9.81E-08
ENSG00000166886	NAB2	4665	-0.7674289	4.56E-06
ENSG00000156162	DPY19L4	286148	-0.766141	1.16E-06
ENSG00000169871	TRIM56	81844	-0.7656148	1.35E-13
ENSG00000160226	CFAP410	755	-0.7646103	0.01210223

ENSG00000068615	REEP1	65055	-0.7645634	0.02660727
ENSG00000170004	CHD3	1107	-0.7641516	3.56E-30
ENSG00000185722	ANKFY1	51479	-0.7633085	2.24E-06
ENSG00000198105	ZNF248	57209	-0.7632058	0.01729429
ENSG00000100445	SDR39U1	56948	-0.7631577	0.00024966
ENSG00000163013	FBXO41	150726	-0.7630302	0.00103177
ENSG00000159267	HLCS	3141	-0.763004	0.00113962
ENSG00000164808	SPIDR	23514	-0.7614312	1.24E-06
ENSG00000161048	NAPEPLD	222236	-0.760864	0.00709095
ENSG00000163069	SGCB	6443	-0.7606893	0.01210223
ENSG00000155096	AZIN1	51582	-0.7604624	2.67E-19
ENSG00000204852	TCTN1	79600	-0.7589818	0.00050627
ENSG00000239382	ALKBH6	84964	-0.7586971	0.03452241
ENSG00000197818	SLC9A8	23315	-0.7583257	0.00406183
ENSG00000005100	DHX33	56919	-0.757974	0.00014249
ENSG00000186815	TPCN1	53373	-0.7567975	0.00060052
ENSG00000198919	DZIP3	9666	-0.7556967	5.83E-05
ENSG00000243317	STMP1	647087	-0.7548269	5.07E-07
ENSG00000164920	OSR2	116039	-0.7546375	0.00185109
ENSG00000122550	KLHL7	55975	-0.754304	0.00688385
ENSG00000223768	LINC00205	642852	-0.7542693	1.32E-05
ENSG00000198938	MT-CO3	4514	-0.7542063	3.47E-08
ENSG00000111727	HCFC2	29915	-0.7538397	0.01446633
ENSG00000156469	MTERF3	51001	-0.7530124	0.00763897
ENSG00000128872	TMOD2	29767	-0.7508274	0.00140877
ENSG00000161395	PGAP3	93210	-0.7507628	0.00269914
ENSG00000004059	ARF5	381	-0.750048	2.16E-11
ENSG00000152402	GUCY1A2	2977	-0.7496824	0.04150669
ENSG00000179562	GCC1	79571	-0.7488186	0.0011081
ENSG00000124181	PLCG1	5335	-0.7481717	1.66E-26
ENSG00000169583	CLIC3	9022	-0.7471056	0.00071228
ENSG00000139410	SDSL	113675	-0.7463022	0.00520606
ENSG00000173436	MICOS10	440574	-0.7461951	0.00049223
ENSG00000074356	NCBP3	55421	-0.7448724	5.65E-05
ENSG00000267796	LIN37	55957	-0.743989	0.03452241
ENSG00000214253	FIS1	51024	-0.7420678	3.80E-14
ENSG00000148824	MTG1	92170	-0.74127	0.00043119
ENSG00000162639	HENMT1	113802	-0.739876	3.02E-08
ENSG00000129219	PLD2	5338	-0.7387914	0.0010732
ENSG00000204228	HSD17B8	7923	-0.7379036	0.02575117
ENSG00000198899	MT-ATP6	4508	-0.7370646	1.06E-06
ENSG00000223959	AFG3L1P	172	-0.7361925	0.01331619

ENSG00000122678	POLM	27434	-0.7354108	0.00087513
ENSG00000138696	BMPR1B	658	-0.735139	0.00597681
ENSG00000285410	GABPB1-IT1	55056	-0.7347402	0.00049223
ENSG00000198794	SCAMP5	192683	-0.7344282	0.00167031
ENSG00000116489	CAPZA1	829	-0.7340026	3.35E-19
ENSG00000092010	PSME1	5720	-0.7319332	1.40E-12
ENSG00000172037	LAMB2	3913	-0.7318572	1.36E-23
ENSG00000182095	TNRC18	84629	-0.7310466	1.34E-19
ENSG00000114098	ARMC8	25852	-0.7306774	0.00025221
ENSG00000019144	PHLDB1	23187	-0.7287873	0.00035223
ENSG00000206527	HACD2	201562	-0.7277555	7.93E-11
ENSG00000133460	SLC2A11	66035	-0.7272699	0.02067744
ENSG00000129103	SUMF2	25870	-0.7272605	4.67E-10
ENSG00000167842	MIS12	79003	-0.7269328	0.007105
ENSG00000135454	B4GALNT1	2583	-0.7267687	0.00758123
ENSG00000106610	STAG3L4	101929736	-0.7264929	0.03536305
ENSG00000186787	SPIN2B	474343	-0.7259687	0.0247251
ENSG00000161955	TNFSF13	8741	-0.7259063	0.00820798
ENSG00000169926	KLF13	51621	-0.7253059	0.00407257
ENSG00000130590	SAMD10	140700	-0.7252502	0.03706963
ENSG00000082497	SERTAD4	56256	-0.7246457	0.00329731
ENSG00000168264	IRF2BP2	359948	-0.7244584	2.34E-06
ENSG00000072201	LNX1	84708	-0.7240147	0.00940777
ENSG00000240184	PCDHGC3	5098	-0.723664	0.00013774
ENSG00000105854	PON2	5445	-0.7230864	5.83E-05
ENSG00000198695	MT-ND6	4541	-0.7227779	0.0006878
ENSG00000164466	SFXN1	94081	-0.7226807	1.73E-14
ENSG00000196497	IPO4	79711	-0.7224026	1.59E-11
ENSG00000241878	PISD	23761	-0.7222739	7.22E-11
ENSG00000064652	SNX24	28966	-0.7220929	0.01248091
ENSG00000100897	DCAF11	80344	-0.720515	2.47E-11
ENSG00000096093	EFHC1	114327	-0.7197568	0.00434579
ENSG00000116977	LGALS8	3964	-0.7193649	3.32E-05
ENSG00000062716	VMP1	81671	-0.7183624	6.84E-22
ENSG00000132749	TESMIN	9633	-0.7180181	0.01248091
ENSG00000034677	RNF19A	25897	-0.7178311	3.67E-05
ENSG00000150961	SEC24D	9871	-0.7176311	0.00071311
ENSG00000110492	MDK	4192	-0.7167733	9.47E-19
ENSG00000162069	BICDL2	146439	-0.7163106	0.00130713
ENSG00000110583	NAA40	79829	-0.7122476	0.00052403
ENSG00000008282	SYPL1	6856	-0.7122104	1.01E-16
ENSG00000198625	MDM4	4194	-0.7108274	0.00025977

ENSG00000175305	CCNE2	9134	-0.7102817	0.007105
ENSG00000151498	ACAD8	27034	-0.7102215	0.00724088
ENSG00000144840	RABL3	285282	-0.7097496	0.00044385
ENSG00000164896	FASTK	10922	-0.7097217	1.01E-05
ENSG00000135596	MICAL1	64780	-0.7096745	0.03966267
ENSG00000138172	CALHM2	51063	-0.7090735	0.00065809
ENSG00000136279	DBNL	28988	-0.7085971	9.50E-09
ENSG00000258701	LINC00638	196872	-0.7085235	0.01852427
ENSG00000130724	CHMP2A	27243	-0.7083732	1.82E-05
ENSG00000170581	STAT2	6773	-0.706636	3.76E-05
ENSG00000176410	DNAJC30	84277	-0.7046281	0.001716
ENSG00000171345	KRT19	3880	-0.7033653	3.96E-15
ENSG00000158467	AHCYL2	23382	-0.7032036	0.00182715
ENSG00000177646	ACAD9	28976	-0.702544	5.20E-05
ENSG00000185875	THNSL1	79896	-0.7014925	0.00047279
ENSG00000108799	EZH1	2145	-0.6976322	0.0054836
ENSG00000104450	SPAG1	6674	-0.6970338	0.02641118
ENSG00000049883	PTCD2	79810	-0.6962744	0.02240834
ENSG00000196937	FAM3C	10447	-0.6960826	0.00769057
ENSG00000124574	ABCC10	89845	-0.6956971	0.00396752
ENSG00000149485	FADS1	3992	-0.6948285	1.46E-06
ENSG00000168827	GFM1	85476	-0.6947091	6.22E-07
ENSG00000092098	RNF31	55072	-0.6947073	0.00017611
ENSG00000130023	ERMARD	55780	-0.6946541	0.03765941
ENSG00000167566	NCKAP5L	57701	-0.6939111	3.95E-06
ENSG00000140400	MAN2C1	4123	-0.6929276	0.00025468
ENSG00000003147	ICA1	3382	-0.6928723	1.49E-06
ENSG00000258315	C17orf49	124944	-0.6927507	0.00059316
ENSG00000106052	TAX1BP1	8887	-0.692559	4.23E-10
ENSG00000146587	RBAK	57786	-0.6922315	0.00025816
ENSG00000183426	NPIPA1	642799	-0.6920624	3.68E-05
ENSG00000164659	ELAPOR2	222223	-0.6920459	4.70E-05
ENSG00000129235	TXNDC17	84817	-0.6916237	0.00101652
ENSG00000006576	PHTF2	57157	-0.6914355	4.51E-05
ENSG00000144554	FANCD2	2177	-0.6912721	0.00051661
ENSG00000235823	OLMALINC	90271	-0.6910931	0.00012192
ENSG00000260027	HOXB7	3217	-0.6909062	0.0054836
ENSG00000131797	CLUHP3	100132341	-0.6907519	0.03765806
ENSG00000181754	AMIGO1	57463	-0.6905443	0.00133227
ENSG00000115073	ACTR1B	10120	-0.6888267	2.05E-07
ENSG00000235106	BRD3OS	266655	-0.6885906	0.00077621
ENSG00000070669	ASNS	440	-0.6881507	0.00659713

ENSG00000161036	LRWD1	222229	-0.6878125	0.00014334
ENSG00000165731	RET	5979	-0.6860773	8.52E-15
ENSG00000113494	PRLR	5618	-0.6854967	4.59E-16
ENSG00000115461	IGFBP5	3488	-0.6844089	1.18E-26
ENSG00000050438	SLC4A8	9498	-0.6843795	0.00166746
ENSG00000183250	LINC01547	84536	-0.6837903	0.04767428
ENSG00000178234	GALNT11	63917	-0.6834231	0.00377517
ENSG00000142686	C1orf216	127703	-0.6821046	0.01505838
ENSG00000250312	ZNF718	255403	-0.6817836	0.04229717
ENSG00000161920	MED11	400569	-0.6815423	0.03168044
ENSG00000054793	ATP9A	10079	-0.6805822	7.82E-39
ENSG00000176155	CCDC57	284001	-0.6800836	2.48E-08
ENSG00000080802	CNOT4	4850	-0.6796648	0.0044546
ENSG00000166997	CNPY4	245812	-0.6796302	0.04496901
ENSG00000091640	SPAG7	9552	-0.6796068	8.09E-05
ENSG00000188690	UROS	7390	-0.6793384	0.00043949
ENSG00000101347	SAMHD1	25939	-0.6793013	0.02190678
ENSG00000167280	ENGASE	64772	-0.6784048	0.02120014
ENSG00000163472	TMEM79	84283	-0.6782364	0.02510471
ENSG00000196275	GTF2IRD2	84163	-0.6779252	0.03354418
ENSG00000064419	TNPO3	23534	-0.6778656	3.31E-06
ENSG00000147654	EBAG9	9166	-0.6764821	0.00022185
ENSG00000135241	PNPLA8	50640	-0.6760061	1.90E-05
ENSG00000205903	ZNF316	100131017	-0.6759835	7.73E-05
ENSG00000101187	SLCO4A1	28231	-0.6754525	0.00468877
ENSG00000130244	FAM98C	147965	-0.6749987	0.01275221
ENSG00000198727	MT-CYB	4519	-0.6745708	5.59E-06
ENSG00000149582	TMEM25	84866	-0.6734638	0.03765941
ENSG00000144355	DLX1	1745	-0.6732012	0.00496734
ENSG00000164830	OXR1	55074	-0.6727222	0.0001451
ENSG00000196924	FLNA	2316	-0.6724708	2.73E-31
ENSG00000075303	SLC25A40	55972	-0.6724024	3.72E-05
ENSG00000121542	SEC22A	26984	-0.6723829	0.01786918
ENSG00000174705	SH3PXD2B	285590	-0.6721806	0.0265555
ENSG00000114353	GNAI2	2771	-0.6715993	2.27E-10
ENSG00000266338	NBPF15	284565	-0.6712837	1.89E-06
ENSG00000101246	ARFRP1	10139	-0.6704817	0.00691947
ENSG00000134504	KCTD1	284252	-0.6700501	0.01077663
ENSG00000241973	PI4KA	5297	-0.6699674	1.47E-06
ENSG00000163611	SPICE1	152185	-0.6698696	0.00617914
ENSG00000100462	PRMT5	10419	-0.6696691	0.00010113
ENSG00000206573	THUMPD3-AS1	440944	-0.6695632	0.02989096

ENSG00000124193	SRSF6	6431	-0.6689402	2.30E-10
ENSG00000075702	WDR62	284403	-0.6688184	0.02514606
ENSG00000159842	ABR	29	-0.6660894	1.08E-09
ENSG00000013016	EHD3	30845	-0.6660377	0.00321526
ENSG00000127804	METTL16	79066	-0.6656906	0.00083786
ENSG00000176903	PNMA1	9240	-0.6655116	8.54E-08
ENSG00000170291	ELP5	23587	-0.665426	0.00067751
ENSG00000143578	CREB3L4	148327	-0.6649738	4.13E-17
ENSG00000171163	ZNF692	55657	-0.6648853	0.02981849
ENSG00000165389	SPTSSA	171546	-0.6643895	4.16E-05
ENSG00000140280	LYSMD2	256586	-0.6641794	0.00083786
ENSG00000255737	AGAP2-AS1	100130776	-0.6628066	0.00730625
ENSG00000004975	DVL2	1856	-0.661134	5.05E-07
ENSG00000141456	PELP1	27043	-0.660891	4.78E-06
ENSG00000087077	TRIP6	7205	-0.6605422	1.31E-11
ENSG00000102901	CENPT	80152	-0.660341	0.01077663
ENSG00000091129	NRCAM	4897	-0.6601613	9.29E-12
ENSG00000151474	FRMD4A	55691	-0.6600919	0.00321526
ENSG00000091140	DLD	1738	-0.6591516	4.90E-14
ENSG00000124942	AHNAK	79026	-0.6584625	9.13E-16
ENSG00000198898	CAPZA2	830	-0.6583233	5.08E-07
ENSG00000105738	SIPA1L3	23094	-0.6580323	1.28E-05
ENSG00000182809	CRIP2	1397	-0.6571249	1.78E-11
ENSG00000172765	TMCC1	23023	-0.6571048	0.00128567
ENSG00000106344	RBM28	55131	-0.6569876	0.00258888
ENSG00000128581	IFT22	64792	-0.6568104	6.34E-05
ENSG00000197879	MYO1C	4641	-0.6567297	9.70E-24
ENSG00000178149	DALRD3	55152	-0.6559756	0.00071472
ENSG00000084764	MAPRE3	22924	-0.6556493	0.0265555
ENSG00000099308	MAST3	23031	-0.6553593	0.00730625
ENSG00000196776	CD47	961	-0.6542885	2.44E-09
ENSG00000135549	PKIB	5570	-0.6542506	4.37E-07
ENSG00000178980	SELENOW	6415	-0.6540317	0.00021192
ENSG00000155090	KLF10	7071	-0.6530356	7.84E-05
ENSG00000144959	NCEH1	57552	-0.6528693	0.04235752
ENSG00000132424	PNISR	25957	-0.6524548	0.00073211
ENSG00000172731	LRRC20	55222	-0.6522132	0.03765806
ENSG00000180891	CUEDC1	404093	-0.6516008	0.00471024
ENSG00000102007	PLP2	5355	-0.6506454	0.0004673
ENSG00000277147	LINC00869	388692	-0.6490986	0.00772749
ENSG00000132199	ENOSF1	55556	-0.6490699	0.00816314
ENSG00000100461	RBM23	55147	-0.6485107	2.78E-05

ENSG00000215440	NPEPL1	79716	-0.6479763	1.51E-15
ENSG00000106211	HSPB1	3315	-0.6477122	8.44E-14
ENSG00000228594	FNDC10	643988	-0.647687	0.00030636
ENSG00000025039	RRAGD	58528	-0.6475683	0.03722334
ENSG00000266173	STRADA	92335	-0.6465069	0.00273199
ENSG00000114023	FAM162A	26355	-0.6461393	0.00098259
ENSG00000198690	FAN1	22909	-0.6454644	0.01337628
ENSG00000196187	TMEM63A	9725	-0.6453412	0.00816314
ENSG00000168453	HR	55806	-0.6445765	0.00075331
ENSG00000256525	POLG2	11232	-0.6442052	0.02221216
ENSG00000139354	GAS2L3	283431	-0.6440025	0.03999195
ENSG00000144455	SUMF1	100130207	-0.6436842	1.28E-05
ENSG00000071794	HLTF	6596	-0.6434029	1.29E-09
ENSG00000128607	KLHDC10	23008	-0.6433658	0.00013214
ENSG00000134215	VAV3	10451	-0.6433268	0.00064076
ENSG00000040531	CTNS	1497	-0.6425754	0.04189013
ENSG00000182504	CEP97	79598	-0.6425164	0.02514606
ENSG00000116793	PHTF1	10745	-0.6421879	0.0007933
ENSG00000181444	ZNF467	168544	-0.64191	0.01204409
ENSG00000166579	NDEL1	81565	-0.641286	0.01014508
ENSG00000184436	THAP7	80764	-0.6410037	0.02221216
ENSG00000061938	TNK2	10188	-0.6404872	2.92E-07
ENSG00000113240	CLK4	57396	-0.6404462	0.02492804
ENSG00000106460	TMEM106B	54664	-0.6397297	1.93E-05
ENSG00000085063	CD59	966	-0.6389633	2.57E-11
ENSG00000168300	PCMTD1	115294	-0.6374767	0.00054438
ENSG00000197448	GSTK1	373156	-0.6370199	3.53E-06
ENSG00000149657	LSM14B	149986	-0.6359043	5.61E-06
ENSG00000113790	EHHADH	1962	-0.6356071	0.03976049
ENSG00000189171	S100A13	6284	-0.6347725	6.46E-12
ENSG00000005020	SKAP2	8935	-0.6334126	6.68E-05
ENSG00000119927	GPAM	57678	-0.6333638	0.01422024
ENSG00000198168	SVIP	258010	-0.633339	0.00109373
ENSG00000139636	LMBR1L	55716	-0.6330889	0.04717609
ENSG00000175591	P2RY2	5029	-0.6328472	0.04475318
ENSG00000106077	ABHD11	83451	-0.632628	5.42E-05
ENSG00000221978	CCNL2	81669	-0.6306632	0.02244274
ENSG00000164164	OTUD4	54726	-0.6296017	0.00024633
ENSG00000099194	SCD	6319	-0.6294544	1.66E-33
ENSG00000107819	SFXN3	81855	-0.6291414	0.0015802
ENSG00000092929	UNC13D	201294	-0.6288211	3.51E-06
ENSG00000167535	CACNB3	784	-0.6286879	2.47E-05

ENSG00000137504	CREBZF	58487	-0.6285464	1.09E-05
ENSG00000163913	IFT122	55764	-0.6283152	0.00617128
ENSG00000138468	SENP7	57337	-0.6280598	0.01177636
ENSG00000148450	MSRB2	22921	-0.627579	0.01194807
ENSG00000059691	GATB	5188	-0.6274877	0.00763067
ENSG00000134255	CEPT1	10390	-0.6274602	0.00078833
ENSG00000128534	LSM8	51691	-0.627293	0.00417469
ENSG00000197763	TXNRD3	114112	-0.6270947	0.02109273
ENSG00000169710	FASN	2194	-0.6266355	5.80E-23
ENSG00000092330	TINF2	26277	-0.6264225	0.00019939
ENSG00000167721	TSR1	55720	-0.6259368	2.71E-06
ENSG00000127774	EMC6	83460	-0.6257748	0.01014508
ENSG00000105887	MTPN	767558	-0.6250864	6.46E-11
ENSG00000105865	DUS4L	11062	-0.6244541	0.03477534
ENSG00000100441	KHNYN	23351	-0.6235401	1.10E-07
ENSG00000146063	TRIM41	90933	-0.6231222	5.22E-06
ENSG00000164172	MOCS2	4338	-0.6230926	0.00148973
ENSG00000124098	FAM210B	116151	-0.6225997	2.78E-13
ENSG00000149499	EML3	256364	-0.6224659	2.01E-05
ENSG00000128609	NDUFA5	4698	-0.6221093	0.00041535
ENSG00000141510	TP53	7157	-0.6212778	6.61E-08
ENSG00000106299	WASL	8976	-0.6209198	2.21E-05
ENSG00000198563	DDX39B	7919	-0.6200611	0.00018127
ENSG00000135723	FHOD1	29109	-0.6189707	0.0004571
ENSG00000059588	TARBP1	6894	-0.6187773	0.04133532
ENSG00000106571	GLI3	2737	-0.6181904	0.00171771
ENSG00000163659	TIPARP	25976	-0.617979	0.01177636
ENSG00000172932	ANKRD13D	338692	-0.6166077	0.00269295
ENSG00000100938	GMPR2	51292	-0.6165272	2.81E-06
ENSG00000142733	MAP3K6	9064	-0.6163117	0.03722334
ENSG00000127993	RBM48	84060	-0.6155462	0.02184047
ENSG00000136631	VPS45	11311	-0.6151206	0.0001509
ENSG00000105655	ISYNA1	51477	-0.6147613	0.00029856
ENSG00000125746	EML2	24139	-0.6142794	0.00043587
ENSG00000075399	VPS9D1	9605	-0.6142708	0.03093098
ENSG00000128585	MKLN1	4289	-0.6140195	0.00156341
ENSG00000142507	PSMB6	5694	-0.6137755	2.95E-06
ENSG00000133597	ADCK2	90956	-0.6130308	0.00203724
ENSG00000214753	HNRNPUL2	221092	-0.6129472	1.95E-06
ENSG00000106399	RPA3	6119	-0.6129107	0.00330612
ENSG00000132549	VPS13B	157680	-0.6128392	7.46E-06
ENSG00000095397	WHRN	25861	-0.6125478	0.00351836

ENSG00000132361	CLUH	23277	-0.6122049	1.59E-09
ENSG00000106605	BLVRA	644	-0.6119468	1.52E-06
ENSG00000169241	SLC50A1	55974	-0.6112471	7.25E-08
ENSG00000122507	BBS9	27241	-0.6112233	0.02944917
ENSG00000104375	STK3	6788	-0.6111044	0.00787491
ENSG00000188997	KCTD21	283219	-0.6109356	0.02335269
ENSG00000154358	OBSCN	84033	-0.6104309	0.00191488
ENSG00000115657	ABCB6	10058	-0.6089701	0.000387
ENSG00000112659	CUL9	23113	-0.6085041	0.00182737
ENSG00000102890	ELMO3	79767	-0.6083625	0.00062147
ENSG00000198478	SH3BGRL2	83699	-0.6079831	0.00751681
ENSG00000168090	COPS6	10980	-0.6077442	2.18E-13
ENSG00000106246	PTCD1	26024	-0.6073472	0.00840565
ENSG00000090006	LTBP4	8425	-0.6071891	0.00041086
ENSG00000157353	FCSK	197258	-0.6067229	0.01177636
ENSG00000058063	ATP11B	23200	-0.6066073	0.00038118
ENSG00000164074	ABHD18	80167	-0.6064611	0.04298931
ENSG00000181191	PJA1	64219	-0.6057781	0.03647463
ENSG00000128524	ATP6V1F	9296	-0.6052422	1.21E-05
ENSG00000175110	MRPS22	56945	-0.6050092	0.00278168
ENSG00000068885	IFT80	57560	-0.6047168	0.00787491
ENSG00000106330	MOSPD3	64598	-0.6039302	0.00213507
ENSG00000106635	BCL7B	9275	-0.6033462	4.97E-05
ENSG00000108528	SLC25A11	8402	-0.6028126	2.50E-05
ENSG00000146416	AIG1	51390	-0.6023119	0.00451936
ENSG00000081014	AP4E1	23431	-0.6021443	3.97E-05
ENSG00000144115	THNSL2	55258	-0.6021191	0.00411918
ENSG00000143624	INTS3	65123	-0.5998847	2.84E-07
ENSG00000111674	ENO2	2026	-0.599075	0.00176414
ENSG00000074755	ZZEF1	23140	-0.5985398	3.22E-05
ENSG00000131779	PEX11B	8799	-0.5984286	4.41E-05
ENSG00000050327	ARHGEF5	7984	-0.5982438	0.00528041
ENSG00000071462	BUD23	114049	-0.5978157	3.74E-05
ENSG00000117305	HMGCL	3155	-0.5970262	0.01818803
ENSG00000147669	POLR2K	5440	-0.5968285	0.00033667
ENSG00000108479	GALK1	2584	-0.5961879	0.000898
ENSG00000102125	TAFAZZIN	6901	-0.5960985	0.01154503
ENSG00000151093	OXSM	54995	-0.5957614	0.04298931
ENSG00000100926	TM9SF1	10548	-0.5955527	3.91E-05
ENSG00000173992	CCS	9973	-0.5951736	0.01207381
ENSG00000143416	SELENBP1	8991	-0.5950086	4.52E-12
ENSG00000165055	METTL2B	55798	-0.5948103	0.01154503

ENSG00000143374	TARS2	80222	-0.5943411	3.67E-05
ENSG00000129250	KIF1C	10749	-0.5931213	2.22E-14
ENSG00000005486	RHBDD2	57414	-0.5921229	1.30E-05
ENSG00000278709	NKILA	105416157	-0.59186	0.0169731
ENSG00000134884	ARGLU1	55082	-0.5918235	0.0054715
ENSG00000105048	TNNT1	7138	-0.5906502	2.98E-05
ENSG00000146457	WTAP	9589	-0.5899574	0.00100488
ENSG00000186591	UBE2H	7328	-0.5897575	1.33E-08
ENSG00000051382	PIK3CB	5291	-0.5880091	0.00016727
ENSG00000120533	ENY2	56943	-0.5874882	0.00021579
ENSG00000176531	PHLDB3	653583	-0.5874149	0.03395864
ENSG00000129197	RPAIN	84268	-0.586829	0.01651638
ENSG00000105254	TBCB	1155	-0.5867549	0.00016727
ENSG00000054148	PHPT1	29085	-0.5863385	0.00042621
ENSG00000106348	IMPDH1	3614	-0.5863381	1.34E-06
ENSG00000171435	KSR2	283455	-0.5861477	0.01352015
ENSG00000176485	PLAAT3	11145	-0.5856629	0.00396239
ENSG00000226137	BAIAP2-DT	440465	-0.5852715	0.00431665
ENSG00000144815	NXPE3	91775	-0.5843179	0.00921856
ENSG00000128563	PRKRIP1	79706	-0.5832555	0.00033005
ENSG00000177628	GBA	2629	-0.5831528	2.40E-07
ENSG00000143797	MBOAT2	129642	-0.5828032	0.00278312
ENSG00000140416	TPM1	7168	-0.5823413	1.82E-08
ENSG00000111077	TNS2	23371	-0.5818185	0.00082789
ENSG00000127995	CASD1	64921	-0.5816214	0.0021448
ENSG00000172594	SMPDL3A	10924	-0.5810515	0.01261352
ENSG00000187164	SHTN1	57698	-0.5807792	2.81E-06
ENSG00000198830	HMGN2	3151	-0.5807529	5.85E-06
ENSG00000164649	CDCA7L	55536	-0.5804414	0.00515932
ENSG00000109107	ALDOC	230	-0.5790635	0.03819509
ENSG00000196411	EPHB4	2050	-0.5784573	9.43E-14
ENSG00000106603	COA1	55744	-0.5784088	0.00059775
ENSG00000110013	SIAE	54414	-0.5780588	0.00461961
ENSG00000138600	SPPL2A	84888	-0.5776587	2.16E-10
ENSG00000172915	NBEA	26960	-0.5771426	0.02811009
ENSG00000184083	FAM120C	54954	-0.5757195	0.00187657
ENSG00000188191	PRKAR1B	5575	-0.5753179	0.0005504
ENSG00000132635	PCED1A	64773	-0.5747831	0.02503422
ENSG00000009830	POMT2	29954	-0.5743629	0.00875562
ENSG00000167107	ACSF2	80221	-0.5742614	0.00135589
ENSG00000132510	KDM6B	23135	-0.5710363	4.92E-05
ENSG00000075945	KIFAP3	22920	-0.5704358	0.01537074

ENSG00000181885	CLDN7	1366	-0.5703544	7.09E-09
ENSG00000123815	COQ8B	79934	-0.5700886	0.01006864
ENSG00000182871	COL18A1	80781	-0.5696852	0.00102213
ENSG00000132507	EIF5A	1984	-0.5686808	0.00010503
ENSG00000172830	SSH3	54961	-0.5674985	1.02E-07
ENSG00000124006	OBSL1	23363	-0.5668551	1.44E-07
ENSG00000069869	NEDD4	4734	-0.5666536	0.00066528
ENSG00000107798	LIPA	3988	-0.5663995	0.00025867
ENSG00000101911	PRPS2	5634	-0.5663843	0.00376294
ENSG00000103168	TAF1C	9013	-0.5663211	0.00011118
ENSG00000269190	FBXO17	115290	-0.5658301	0.03437462
ENSG00000163781	TOPBP1	11073	-0.5652846	3.16E-05
ENSG00000122515	ZMIZ2	83637	-0.5651773	1.21E-05
ENSG00000226752	CUTALP	253039	-0.564992	0.00226924
ENSG00000178096	BOLA1	51027	-0.5641716	0.00118534
ENSG00000121957	GPSM2	29899	-0.563791	0.00209199
ENSG00000135249	RINT1	60561	-0.5633126	0.00302276
ENSG00000159216	RUNX1	100506403	-0.5629591	0.03706426
ENSG00000008256	CYTH3	9265	-0.5618207	0.01141159
ENSG00000138495	COX17	10063	-0.5612893	0.01235082
ENSG00000143590	EFNA3	1944	-0.5610335	0.00783106
ENSG00000006125	AP2B1	163	-0.5602529	3.88E-08
ENSG00000087111	PIGS	94005	-0.5601034	0.00010411
ENSG00000069974	RAB27A	5873	-0.559749	0.03864362
ENSG00000259431	THTPA	79178	-0.5578993	0.04347034
ENSG00000077044	DGKD	8527	-0.5578494	0.00165491
ENSG00000197782	ZNF780A	284323	-0.5578374	0.04893335
ENSG00000143093	STRIP1	85369	-0.5566987	0.00159202
ENSG00000048392	RRM2B	50484	-0.5566701	4.10E-06
ENSG00000276293	PIP4K2B	8396	-0.5565193	0.0001603
ENSG00000197296	FITM2	128486	-0.5564025	0.04706979
ENSG00000148120	AOPEP	84909	-0.5558884	0.00011566
ENSG00000092108	SCFD1	23256	-0.5556849	0.00419977
ENSG00000179163	FUCA1	2517	-0.5553609	0.04023933
ENSG00000125534	PPDPF	79144	-0.5545049	1.66E-07
ENSG00000164934	DCAF13	25879	-0.5544899	3.60E-06
ENSG00000012822	CALCOCO1	57658	-0.5544199	0.00107576
ENSG00000006530	AGK	55750	-0.554418	0.02890644
ENSG00000132388	UBE2G1	7326	-0.5543331	0.00049403
ENSG00000127952	STYXL1	51657	-0.553629	0.00917869
ENSG00000106290	TAF6	6878	-0.5536232	8.71E-08
ENSG00000235437	LINC01278	92249	-0.5534532	0.026305

ENSG00000081803	CADPS2	93664	-0.5533271	0.03437462
ENSG00000263956	NBPF11	200030	-0.5526561	0.00491333
ENSG00000143314	MRPL24	79590	-0.5520395	4.74E-05
ENSG00000132541	RIDA	10247	-0.5511277	0.00735105
ENSG00000198420	TCAF1	9747	-0.5510159	3.05E-08
ENSG00000125459	MSTO1	55154	-0.5503775	0.02174386
ENSG00000119401	TRIM32	22954	-0.5500872	0.02733966
ENSG00000134262	AP4B1	10717	-0.5494041	0.00046135
ENSG00000173156	RHOD	29984	-0.5492512	0.0001603
ENSG00000159199	ATP5MC1	516	-0.5465039	0.00078566
ENSG00000104517	UBR5	51366	-0.5459674	3.01E-10
ENSG00000219545	UMAD1	729852	-0.5453869	0.04347034
ENSG00000179387	ELMOD2	255520	-0.545265	0.0061222
ENSG00000105607	GCDH	2639	-0.5441557	0.03723275
ENSG00000173281	PPP1R3B	79660	-0.5436572	0.00510163
ENSG00000198538	ZNF28	7576	-0.5435082	0.04347592
ENSG00000156471	PTDSS1	9791	-0.5425358	4.37E-06
ENSG00000108469	RECQL5	9400	-0.5424505	0.0012972
ENSG00000105793	GTPBP10	101927446	-0.5420193	0.00306772
ENSG00000068724	TTC7A	57217	-0.540857	0.02626305
ENSG00000108773	KAT2A	2648	-0.5404988	0.00510103
ENSG00000169738	DCXR	51181	-0.5403578	4.83E-07
ENSG00000156170	NDUFAF6	137682	-0.5403526	0.02733966
ENSG00000129255	MPDU1	9526	-0.5398479	0.00381378
ENSG00000005206	SPPL2B	56928	-0.5393666	0.03723275
ENSG00000135931	ARMC9	80210	-0.5392109	0.035796
ENSG00000170027	YWHAG	7532	-0.5391082	2.00E-10
ENSG00000128833	MYO5C	55930	-0.5389588	3.06E-10
ENSG00000164924	YWHAZ	7534	-0.5389096	2.05E-36
ENSG00000143443	C1orf56	54964	-0.5386018	0.04889087
ENSG00000197324	LRP10	26020	-0.5383886	1.17E-12
ENSG00000196227	FAM217B	63939	-0.5381019	1.22E-05
ENSG00000114446	IFT57	55081	-0.5372519	0.00549009
ENSG00000074590	NUAK1	9891	-0.5369389	0.00510163
ENSG00000069956	MAPK6	5597	-0.536829	3.68E-08
ENSG00000161956	SENP3	26168	-0.5359943	0.00015144
ENSG00000112773	TENT5A	55603	-0.5358951	0.01382026
ENSG00000104361	NIPAL2	79815	-0.5349818	0.00822115
ENSG00000127989	MTERF1	7978	-0.5349777	0.0401492
ENSG00000178381	ZFAND2A	90637	-0.5348163	0.01866435
ENSG00000106683	LIMK1	3984	-0.5341481	0.01025935
ENSG00000248008	NRAV	100506668	-0.5337265	0.04889087

ENSG00000066629	EML1	2009	-0.5334129	0.02343272
ENSG00000119699	TGFB3	7043	-0.5332149	0.00410165
ENSG00000119661	DNAL1	83544	-0.5330095	0.03443983
ENSG00000100888	CHD8	57680	-0.5329027	7.09E-06
ENSG00000189077	TMEM120A	83862	-0.5324098	0.00265747
ENSG00000124155	PIGT	51604	-0.5320025	5.97E-06
ENSG00000132825	PPP1R3D	5509	-0.5319349	0.00104802
ENSG00000004864	SLC25A13	10165	-0.5316104	0.00022255
ENSG00000278970	HEIH	100859930	-0.5301644	0.0017261
ENSG00000136935	GOLGA1	2800	-0.529283	0.00367897
ENSG00000129484	PARP2	10038	-0.5286676	0.0401492
ENSG00000172889	EGFL7	51162	-0.5285137	0.00659419
ENSG00000198804	MT-CO1	4512	-0.5284279	0.00053482
ENSG00000109118	PHF12	57649	-0.5284143	0.00108535
ENSG00000055163	CYFIP2	26999	-0.5282193	6.16E-05
ENSG00000141753	IGFBP4	3487	-0.5281986	7.32E-09
ENSG00000203667	COX20	116228	-0.5277634	0.03184884
ENSG00000205544	TMEM256	254863	-0.527608	0.01188071
ENSG00000053371	AKR7A2	8574	-0.5264211	0.00016814
ENSG00000114026	OGG1	4968	-0.5256873	0.01484569
ENSG00000161960	EIF4A1	1973	-0.5249681	1.15E-15
ENSG00000165804	ZNF219	51222	-0.5238703	0.0008471
ENSG00000134265	NAPG	8774	-0.5238454	0.00265747
ENSG00000101751	POLI	11201	-0.5231404	0.02838736
ENSG00000131089	ARHGEF9	23229	-0.5227834	0.01230313
ENSG00000163517	HDAC11	79885	-0.5224305	0.0070849
ENSG00000105419	MEIS3	56917	-0.5222009	0.03052006
ENSG00000187634	SAMD11	148398	-0.5220767	0.00104548
ENSG00000111602	TIMELESS	8914	-0.5210527	4.06E-05
ENSG00000122565	CBX3	11335	-0.5207414	3.11E-11
ENSG00000107821	KAZALD1	81621	-0.5203102	0.00354328
ENSG00000122512	PMS2	5395	-0.5203088	0.00044746
ENSG00000040633	PHF23	79142	-0.5201168	0.00090845
ENSG00000027847	B4GALT7	11285	-0.5191974	0.01793302
ENSG00000124222	STX16	8675	-0.518943	3.19E-13
ENSG00000115738	ID2	3398	-0.5188767	0.0032731
ENSG00000104998	IL27RA	9466	-0.5187039	0.03443983
ENSG00000196943	NOP9	161424	-0.5185512	0.00142895
ENSG00000010404	IDS	3423	-0.5181021	1.26E-06
ENSG00000144749	LRIG1	26018	-0.5175542	0.00040102
ENSG00000160753	RUSC1	23623	-0.5175011	2.59E-07
ENSG00000123297	TSFM	10102	-0.5173523	0.0004805

ENSG00000114405	C3orf14	57415	-0.5171237	4.97E-09
ENSG00000163703	CRELD1	78987	-0.5166479	0.03543842
ENSG00000205133	TRIQK	286144	-0.5164801	4.90E-07
ENSG00000042062	RIPOR3	140876	-0.5163611	0.00227808
ENSG00000155366	RHOC	389	-0.5157087	1.69E-12
ENSG00000175826	CTDNEP1	23399	-0.5156642	9.61E-05
ENSG00000108559	NUP88	4927	-0.5154865	0.00704222
ENSG00000076248	UNG	7374	-0.5152513	2.18E-09
ENSG00000139629	GALNT6	11226	-0.5151701	2.20E-08
ENSG00000168234	TTC39C	125488	-0.5133674	0.02604941
ENSG00000157259	GATAD1	57798	-0.5130035	2.82E-06
ENSG00000160180	TFF3	7033	-0.5129854	0.00158888
ENSG00000106105	GARS1	2617	-0.5129361	4.17E-05
ENSG00000197157	SND1	27044	-0.5126984	2.66E-08
ENSG00000078687	TNRC6C	57690	-0.5126465	0.00089914
ENSG00000077150	NFKB2	4791	-0.512344	0.00338324
ENSG00000109586	GALNT7	51809	-0.5118907	0.00012215
ENSG00000107331	ABCA2	20	-0.5109272	3.04E-08
ENSG00000131187	F12	2161	-0.5105319	0.01139809
ENSG00000148356	LRSAM1	90678	-0.5101836	0.0061023
ENSG00000168938	PPIC	5480	-0.5099827	0.00014451
ENSG00000108506	INTS2	57508	-0.509964	2.95E-05
ENSG00000078177	N4BP2	55728	-0.5099294	0.01536884
ENSG00000175662	TOM1L2	146691	-0.5097334	0.00016666
ENSG00000158793	NIT1	4817	-0.5095735	0.03975562
ENSG00000178605	GTPBP6	8225	-0.5081283	0.00083597
ENSG00000174306	ZHX3	23051	-0.5056564	1.22E-07
ENSG00000100890	PRORP	9692	-0.5056538	0.00697317
ENSG00000146535	GNA12	2768	-0.504718	0.00010946
ENSG00000075407	ZNF37A	7587	-0.5044756	0.02694261
ENSG00000127980	PEX1	5189	-0.504374	0.00936061
ENSG00000100941	PNN	5411	-0.5035595	0.0006766
ENSG00000122687	MRM2	29960	-0.5035328	0.00158888
ENSG00000128989	ARPP19	10776	-0.5026174	4.22E-19
ENSG00000092036	HAUS4	54930	-0.5015726	0.0011466
ENSG00000102265	TIMP1	7076	-0.5015058	0.01342008
ENSG00000177508	IRX3	79191	-0.5012176	6.97E-05
ENSG00000138594	TMOD3	112268148	-0.5010266	5.48E-11
ENSG00000135924	DNAJB2	3300	-0.5004371	0.00164127
ENSG00000156467	UQCRB	7381	-0.5003387	6.41E-08
ENSG00000121851	POLR3GL	84265	-0.5002629	0.01300361
ENSG00000116128	BCL9	607	-0.5001058	0.00069319
