# Supplementary figures and images for: The Ephrin tyrosine kinase a3 (EphA3) is a novel mediator of RAGE-prompted motility of breast cancer cells
Source: J Exp Clin Cancer Res. 2023 Jul 12;42:164. doi: 10.1186/s13046-023-02747-5 (PMC10337103; doi:10.1186/s13046-023-02747-5)

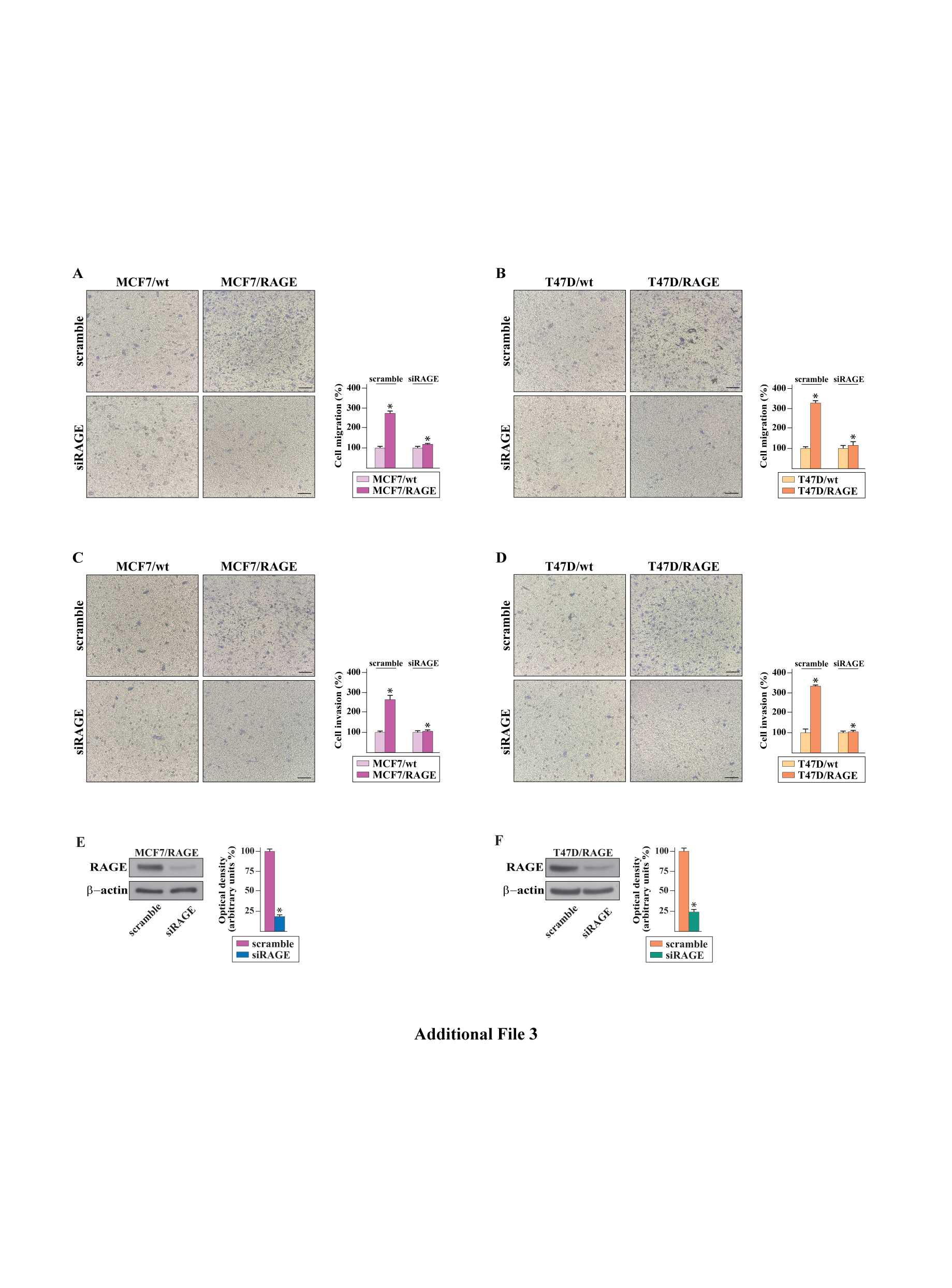

Supplement: Supplementary file 3 — Additional file 3: RAGE silencing impairs the migratory and invasivebehavior of RAGE-overexpressing BC cells. Transwell migration (A-B) andinvasion (C-D) assays in wild type and RAGE-overexpressing MCF7 and T47D cells.Cells were counted in at least 5 random fields in three independent experimentsperformed in triplicate, as quantified in the side panels. Scale bar 200 μm. Efficacy of RAGEsilencing in MCF7/RAGE (E) and T47D/RAGE (F) cells. Side panels showdensitometric analysis of the blots normalized to β-actin, which was used as aloading control. Results shown are representative of at least three independentexperiments. (*) indicates p < 0.05. [file 13046_2023_2747_MOESM3_ESM.tif]

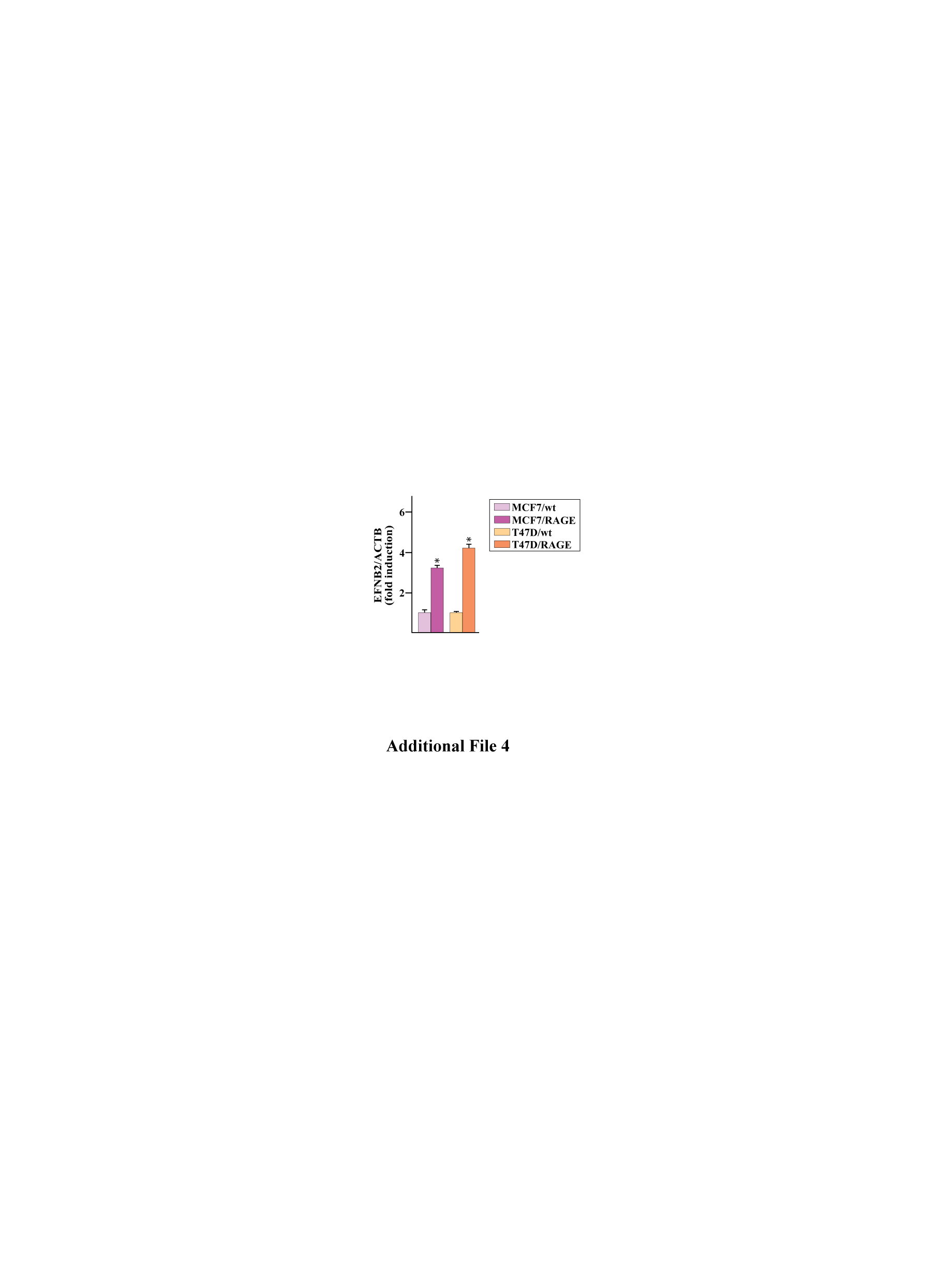

Supplement: Supplementary file 4 — Additonal file 4: Analysis of the expression of Ephrin B2 in wild type and RAGE-overexpressing MCF7 cells. mRNA expression of Ephrin B2 in RAGE-overexpressing (MCF7/RAGE) respect to wild type (MCF7/wt) cells, as ascertained by real-time PCR. Values are normalized to the actin beta (ACTB) expression and shown as fold changes of mRNA expression in RAGE-overexpressing respect to wild type cells. Values represent the mean ± SD of three independent experiments performed in triplicate. (*) indicates p < 0.05. [file 13046_2023_2747_MOESM4_ESM.tif]

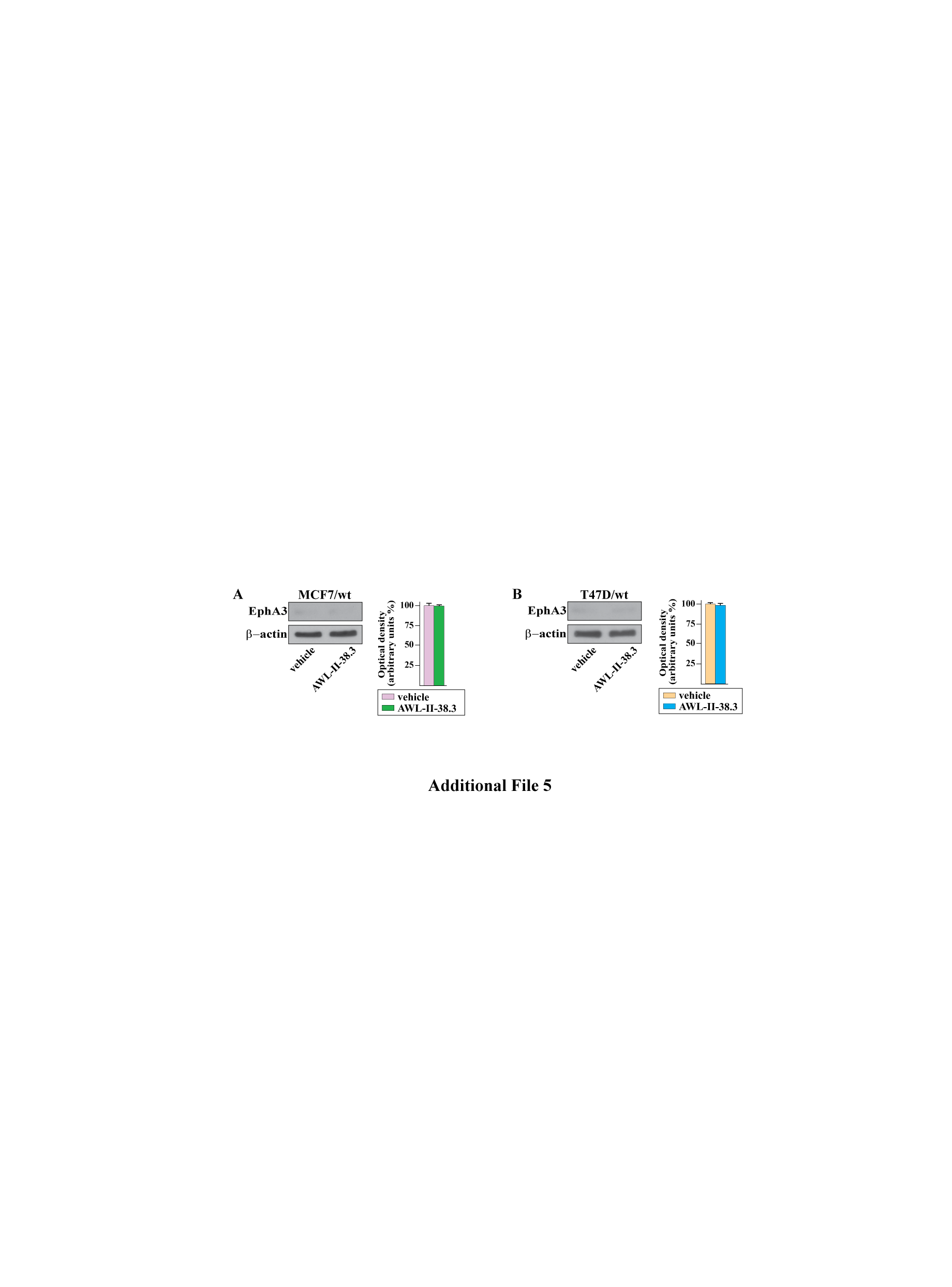

Supplement: Supplementary file 5 — Additional file 5: The EphA3 inhibitor AWL-II-38.3 does not modify the whole protein levels of EphA3. Immunoblots of EphA3 in MCF7/RAGE (A) and T47D/RAGE (B) cells in the presence or absence of the EphA3 inhibitor AWL-II-38.3. Side panels show densitometric analysis of the blots normalized to β-actin, which was used as a loading control. Values represent the mean ± SD of three independent experiments performed in triplicate. [file 13046_2023_2747_MOESM5_ESM.tif]
